# A multienzyme-mimicking nanoplatform induces disulfidptosis/cuproptosis/apoptosis for tumor therapy

**DOI:** 10.1093/nsr/nwag316

**Published:** 2026-05-27

**Authors:** Wei-Jie Sun, Jie Lin, Xiao-Kang Lu, Jin Fu, Jia-Wen Li, Hong-Min Zhu, Feng-Qi Zhou, Si-Ying Ma, Guo-Bao Ning, Bi-Lian Li, Hang Chen, Jie Li, Yu-Xun Lu, Xiang Lai, Lian Jin, Bai-Cheng Lu, Can-Peng Li, Ya-Ping Zhang, Hui Zhao

**Affiliations:** Bio-X Center for Interdisciplinary Innovation, Yunnan University, Kunming 650500, China; State Key Laboratory of Genetic Evolution & Animal Models, Kunming Institute of Zoology, Chinese Academy of Sciences, Kunming 650201, China; Kunming College of Life Science, University of the Chinese Academy of Sciences, Kunming 650204, China; Bio-X Center for Interdisciplinary Innovation, Yunnan University, Kunming 650500, China; School of Chemical Science and Technology, Yunnan University, Kunming 650500, China; Pathology Department, The Second People’s Hospital of Yunnan Province, Kunming 650021, China; Bio-X Center for Interdisciplinary Innovation, Yunnan University, Kunming 650500, China; School of Chemical Science and Technology, Yunnan University, Kunming 650500, China; School of Chemical Science and Technology, Yunnan University, Kunming 650500, China; Bio-X Center for Interdisciplinary Innovation, Yunnan University, Kunming 650500, China; School of Chemical Science and Technology, Yunnan University, Kunming 650500, China; School of Chemical Science and Technology, Yunnan University, Kunming 650500, China; School of Chemical Science and Technology, Yunnan University, Kunming 650500, China; State Key Laboratory of Genetic Evolution & Animal Models, Kunming Institute of Zoology, Chinese Academy of Sciences, Kunming 650201, China; Kunming College of Life Science, University of the Chinese Academy of Sciences, Kunming 650204, China; School of Chemical Science and Technology, Yunnan University, Kunming 650500, China; School of Chemical Science and Technology, Yunnan University, Kunming 650500, China; Bio-X Center for Interdisciplinary Innovation, Yunnan University, Kunming 650500, China; Bio-X Center for Interdisciplinary Innovation, Yunnan University, Kunming 650500, China; Bio-X Center for Interdisciplinary Innovation, Yunnan University, Kunming 650500, China; Bio-X Center for Interdisciplinary Innovation, Yunnan University, Kunming 650500, China; State Key Laboratory of Genetic Evolution & Animal Models, Kunming Institute of Zoology, Chinese Academy of Sciences, Kunming 650201, China; Bio-X Center for Interdisciplinary Innovation, Yunnan University, Kunming 650500, China

**Keywords:** nanozymes, tumor microenvironment, cancer enzyodynamic therapy, metal-organic frameworks, acetaminophen, cell death pathways

## Abstract

The distinct characteristics of the tumor microenvironment (TME) provide great potential for valuable tumor-targeting therapies using enzymes. Herein, MOF-818 with a diameter of 139 nm is synthesized and exhibits multienzyme-mimicking activities, including peroxidase, catalase, glutathione oxidase, glucose oxidase, superoxide dismutase, and tyrosinase (TYR)-like activities. Based on these enzymatic activities, MOF-818 catalyzes an entire process that progresses through self-generated substrates and cyclic catalytic reactions, and notably induces TME remodeling. Synchronously, for the first time reported in nanozymes, MOF-818 activates the prodrug acetaminophen (APAP) through its TYR-like activity, further increasing reactive oxygen species accumulation. These features contribute to MOF-818’s antitumor activity by activating multiple combined programmed cell death pathways: apoptosis, disulfidptosis, and cuproptosis. The MOF-818 nanoplatform, 3-methyladenine (3-MA), THP-1 cell membranes, and a fusion protein of Fc and TNF-related apoptosis-inducing ligand (Fc-TRAIL) were integrated to construct 3-MA@MOF-818@CM-Fc-TRAIL nanoparticles (3MCT NPs). These NPs target tumor cells and, when coupled with APAP, show efficient therapeutic effects with good biosafety. Our findings indicate that the MOF-818 could function as a multienzyme-mimetic scaffold for engineering targeted nanoplatforms that co-deliver combinatorial therapeutic agents, thereby enabling combined antitumor therapy.

## INTRODUCTION

Cancer poses a major global health threat [1], with abnormal tumor microenvironment (TME) causing traditional chemotherapy resistance [2]. The TME is characterized by hypoxia, mild acidity, and excessive accumulation of glutathione (GSH) and hydrogen peroxide (H_2_O_2_) [3]. These conditions enhance cancer cell antioxidant defenses while also providing valuable and specific targets for cancer therapy [4].

‘Enzyodynamic therapy’ has drawn wide attention as an effective tumor treatment by remodeling the TME [5]. This therapy utilizes enzymes to generate harmful reactive oxygen species (ROS), thereby killing cancer cells. Nanozymes are nanomaterials with enzyme-like characteristics [6]. Characterized by adjustable catalytic activity, high stability, low cost, high biocompatibility, and mass-producibility, nanozymes with glutathione oxidase (GSHOx), glucose oxidase (GOx), catalase (CAT), and peroxidase (POD) have emerged as a promising alternative to natural enzymes for cancer enzyodynamic therapy [7,8]. Therefore, a single nanozyme system with multiple enzymes should be developed for tumor cell elimination.

As a component of cancer enzyme therapeutics, the extensively used, non-toxic prodrug acetaminophen (APAP) can be activated in the presence of O_2_ by tyrosinase (TYR) to form cytotoxic 4-acetamido-*o*-ben zoquinone (AOBQ), accompanied by GSH depletion and ROS generation [9,10]. However, the hypoxic environment of the tumor and the low expression levels of TYR in most cancer cells hinder using this approach in anti-neoplastic treatment.

Metal-organic framework (MOF) nanomaterials are promising for biomedical applications. Recently, a Zr-Cu bimetallic MOF (MOF-818) was synthesized with an octahedral crystal morphology and an average particle size of 400 nm [11]. MOF-818 has demonstrated remarkable stability in aqueous dispersions, excellent biocompatibility, and enzyme-like catalytic activity similar to that of catechol oxidase, superoxide dismutase (SOD), CAT, and POD [12–15]. Leveraging these catalytic properties, MOF-818 has exhibited functionality in biosensing [15–18], and as a therapeutic for diabetic chronic wounds and infected burn wounds [12,19]. However, MOF-818 has not yet been used in cancer treatment despite its tremendous potential for remodeling the TME.

In this study, we modified MOF-818 to obtain nanomaterials with an average particle size of 139 nm, and for the first time revealed its TYR, GOx, and GSHOx mimetic activities. The nanosized MOF-818 mimicked the activity of six enzymes, POD, CAT, GOx, GSHOx, SOD, and TYR, and reinforced the TME remodeling in human breast cancer cell line HCC1806 through a circle reaction. The entire process proceeded through self-generated substrates and cyclic catalytic reactions. Consequently, this cascade catalytic reaction is described as a ‘rolling circle catalysis’ (RCC) reaction. Additionally, relieving the intracellular hypoxia and increasing levels of H_2_O_2_ accelerated the conversion of APAP into the highly toxic AOBQ and *N*-acetyl-*p*-benzoquinone imine (NAPQI) molecules, which further promoted GSH consumption and ROS accumulation. Based on the nanoplatform of MOF-818, we used the anti-autophagy drug 3-methyladenine (3-MA), THP-1 cell membranes (CMs), and a fusion protein of Fc and TNF-related apoptosis-inducing ligand (Fc-TRAIL) to successfully assemble to form 3-MA@MOF-818@CM-Fc-TRAIL (3MCT) nanoparticles (NPs). As a proof-of-concept, Fc-TRAIL significantly increased the 3MCT NPs accumulation in tumors in mice after tail vein injection, and the nanomedicine combination of 3MCT NPs and APAP exhibited efficient tumor catalytic treatment with minimal side effects in HCC1806 tumor-bearing mice. The treatment was confirmed to induce apoptosis, disulfidptosis, and cuproptosis through a set of comprehensive *in vitro* and *in vivo* experiments.

## RESULTS

### Preparation and characterization of the MOF-818 nanozyme

**Figure 1.**
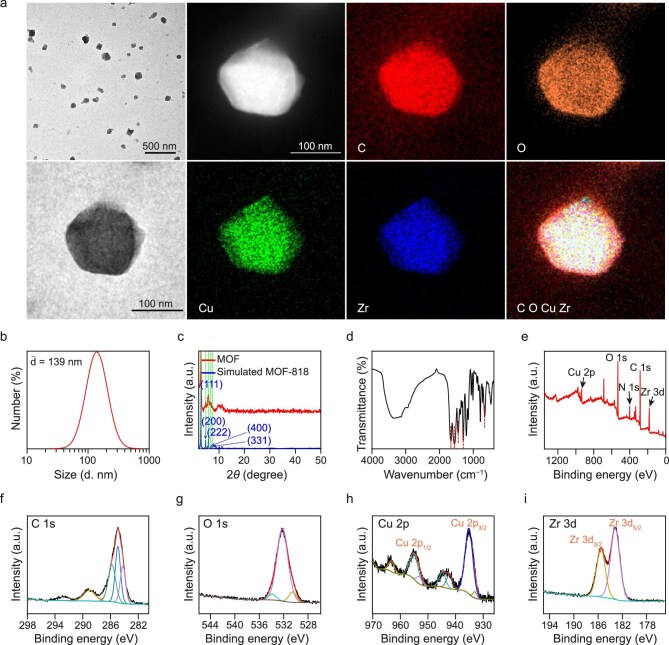
Characterization of MOF-818. (a) Transmission electron microscopy (TEM) images and elemental mapping results. (b) Dynamic light scattering (DLS) results. (c) X-ray diffraction (XRD) patterns. (d) Fourier transform infrared (FT-IR) spectroscopy. (e–i) X-ray photoelectron spectroscopy (XPS) wide-scan, C 1s, O 1s, Cu 2p, and Zr 3d results.

To facilitate cancer cell endocytosis, we prepared MOF-818 in a smaller size. Transmission electron microscopy (TEM) images revealed the octahedral morphology of MOF-818 (Fig. 1a) with an average diameter of 139 nm (Fig. 1b). This size is smaller than that previously reported MOF-818 [11]. The difference may be attributed to changes in the formation and growth rates of MOF crystal nuclei, caused by metal ion concentration variation during the stepwise synthesis [20]. Elemental mapping revealed that C, O, Cu, and Zr elements were distributed across the MOF-818 sample (Fig. 1a). The X-ray diffraction (XRD) pattern indicated that the crystal structure of MOF-818 matched well with that of simulated MOF-818 (Fig. 1c). As shown in Fig. 1d, Fourier transform infrared (FT-IR) spectroscopy of MOF-818 showed a peak at 663 cm^−1^ representing the stretching mode of O–Zr–O, and the absorption at 1656 cm^−1^ was attributed to the symmetric stretching of C=O [15]. The spectral band at 1553 cm^−1^ was attributed to the C=N stretching vibration of a pyrazole ring, and the peaks at 1448 and 1296 cm^−1^ were assigned to the skeleton vibration of a pyrazole ring and the C–N/C–O stretching vibration, respectively [21]. The chemical composition of MOF-818 was verified by X-ray photoelectron spectroscopy (XPS) (Fig. 1e–i). The Cu 2p spectrum showed binding energy peaks at 934.9 and 954.3 eV that were attributed to Cu 2p_3/2_ and Cu 2p_1/2_, respectively, which are characteristic of Cu^2+^. Furthermore, the peak located at 932.7 eV and 952.5 eV was assigned to Cu^+^ (Fig. 1h). The Zr 3d spectrum revealed that the 3d_3/2_ and 3d_5/2_ states corresponding to 182.7 and 185.2 eV, respectively, indicating the presence of Zr^4+^ in MOF-818 (Fig. 1i).

The N_2_ sorption isotherm analysis showed that our synthesized MOF-818 exhibits a Brunauer-Emmett-Teller surface area of was 876.8 m^2^ g^−^^1^ (Fig. S1), which is larger than the values previously reported for MOF-818 (ranging from 440.39 m^2^ g^−^^1^ [18] to 743.5 m^2^ g^−^^1^ [13]). This high surface area is likely conducive to substrate interaction and may enhance its catalytic activity.

### Multienzyme-like activities of MOF-818

**Figure 2.**
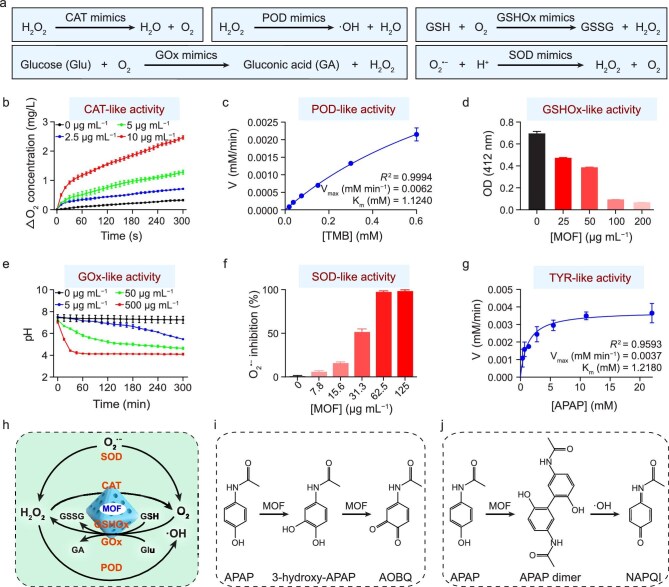
Multienzyme-mimicking activities of the MOF-818 nanozyme. (a) Schematic illustration of the catalytic reactions. CAT, catalase; POD, peroxidase; GSHOx, glutathione oxidase; GOx, glucose oxidase; SOD, superoxide dismutase. (b) CAT-like activity of MOF-818 presenting time-dependent O_2_ production in the presence of different concentrations of MOF-818. (c) Michaelis-Menten curves of MOF-818 as a POD mimic in the presence of different 3,3′,5,5′-tetramethylbenzidine (TMB) concentrations and 100 mM H_2_O_2_. (d) GSHOx-like activity of MOF-818. (e) GOx-like activity of MOF-818 with pH changes in the presence of different concentrations of MOF-818 and 1 mg mL^−1^ of glucose. (f) SOD-like activity of MOF-818 by O_2_^·−^ detection using a WST-8 assay in the presence of different concentrations of MOF-818. (g) Michaelis-Menten curves of MOF-818 as a TYR mimic using acetaminophen (APAP) as a substrate. (h) Schematic illustration of the rolling circle catalysis reaction induced by MOF-818. Schematic diagrams of catalytic process from APAP to 4-acetamido-*o*-ben zoquinone (AOBQ) (i) and *N*-acetyl-*p*-benzoquinone imine (NAPQI) (j) by MOF-818. Data are given as mean ± SD (*n* = 3).

Given the high level of H_2_O_2_ in tumor tissue, the CAT-like activity of MOF-818, which catalyzed the conversion of H_2_O_2_ into O_2_ (Fig. 2a), was initially confirmed using a dissolved oxygen meter (Fig. 2b). H_2_O_2_ can also be consumed by POD, producing highly oxidizing ·OH (Fig. 2a). As shown in Fig. 2c and Fig. S2 and Table S1, the MOF-818 exhibits a performance POD-like activity, demonstrating a lower Michaelis-Menten constant (*K*_m_) and a comparable maximal reaction velocity (*V*_max_) relative to natural horseradish peroxidase and other nanozymes, including reported MOF-818, Pd-Fe_3_O_4__middle, Pt hollow, and Fe-MIL-88B-NH_2_.

Another prominent feature of tumor tissue is the high concentration of GSH, which scavenges ROS and weakens the anti-neoplastic effect [5]. GSHOx catalyzes the conversion of GSH into oxidized glutathione (GSSG), followed by consumption of O_2_ and the production of H_2_O_2_ (Fig. 2a). As depicted in Fig. 2d, MOF-818 effectively consumed GSH in a concentration-dependent manner.

As tumor cells require substantial glucose to produce energy for proliferation, cutting off the glucose supply is an effective anti-neoplastic strategy [22]. We observed that MOF-818 catalyzed the conversion of glucose into gluconic acid, exhibiting a significant reaction at pH 5.5 (Fig. S3). Since GOx mimics can produce gluconic acid, the decrease in pH can be used as an indicator of the GOx-like activity of MOF-818. As expected, the pH of the reaction system decreased with the increase of MOF-818 concentration (Fig. 2e), demonstrating the excellent GOx-like activity of MOF-818.

The O_2_^·−^ generated by the mitochondrial respiratory chain can be catalyzed by SOD-like enzymes to produce O_2_ and H_2_O_2_, improving the hypoxic microenvironment of tumors and providing substrates for subsequent multienzyme cascade catalysis [23]. As presented in Fig. 2f, the inhibition rate increased rapidly with the increase of MOF-818 concentration using SOD assay kit WST-8, confirming the SOD-like activity of MOF-818.

MOF-818 has a high-specificity catechol oxidase [13], which catalyzes the oxidation of *o*-diphenol to the corresponding *o*-quinone derivatives (Fig. S4a). We first confirmed the catechol oxidase-like activity of MOF-818 using *o*-diphenol (L-dopa) as a substrate (Fig. S4b). Remarkably, using monophenols as substrates (APAP, L-tyrosine, and phenol), we found that MOF-818 exhibited TYR-like activity by catalyzing the oxidation of monophenol to the product *o*-quinone (Fig. 2g and Fig. S4c and d), which has not been reported in other nanomaterials.

In summary, MOF-818 exhibited activities analogous to CAT, POD, GSHOx, GOx, SOD, and TYR that may trigger cascade catalysis in tumor cells, demonstrating the potential to remodel the TME and activate the prodrug APAP.

### TME remodeling induced by MOF-818 coupled with APAP

To determine whether the multienzyme-mimicking activities of MOF-818 can efficiently coexist under the same condition, we assessed six enzymatic activities under a series of conditions varied in pH (6.5 and 7.4) and H_2_O_2_ concentrations (10 nM, 1 μM, and 100 μM), corresponding to normal cellular, intracellular tumor, and TME, respectively [24–26]. The results demonstrated that the MOF-818 (5 μg mL^−1^) exhibited six coexisting enzyme-mimicking activities in both simulated tumor intracellular conditions (pH 7.4) and TME (pH 6.5) in the presence of 100 μM H_2_O_2_ (Fig. S5).

Next, we evaluated MOF-818’s catalytic ability and effect on TME remodeling in HCC1806 cancer cells. We found that the O_2_^·−^ level in HCC1806 tumor cells significantly decreased after being treated with MOF-818 (Fig. S6a and e), indicating that MOF-818 effectively exerted SOD-like activity to produce H_2_O_2_. Then, the excess H_2_O_2_ was consumed by MOF-818-induced CAT and POD-like activities (Fig. S6b and f), which produced a large amount of O_2_ (Fig. S6c and g) and highly toxic ·OH (Fig. S6d and h). Moreover, by using the produced O_2_, the GSHOx-like activity of MOF-818 consumed GSH (Fig. S6i). Simultaneously, the produced O_2_ further oxidized glucose to gluconic acid through the GOx-like activity of MOF-818 (Fig. S6j), which continuously generated H_2_O_2_. Ultimately, the resultant H_2_O_2_ as a substrate re-entered the next reactions catalyzed by the CAT-like and POD-like activities of MOF-818. Therefore, the entire process proceeded through self-generated substrates and cyclic catalytic reactions, which was proposed as rolling circle catalysis, RCC (Fig. 2h).

We systemically analyzed the reaction products of APAP activated by the TYR-like activity of MOF-818 using high performance liquid chromatography-mass spectrometer (HPLC-MS). As shown in Fig. S7a–g, after HCC1806 cells were incubated with APAP and MOF-818, APAP was activated by MOF-818 to produce 3-hydroxy-APAP, followed by oxidization to AOBQ. The same result was also verified in tubes after incubation of MOF-818 with APAP (Figs S7h–k and S8). Therefore, MOF-818 exhibits monophenol oxidase activity to convert APAP into the intermediate product 3-hydroxy-APAP, and performs polyphenol oxidase activity to generate AOBQ (Fig. 2i). Additionally, the ·OH generated *via* a Fenton reaction can activate APAP and produce the toxic metabolite NAPQI to cause oxidative damage [27]. Given that massive amounts of highly toxic ·OH are generated by the POD-like activity of MOF-818, the production of NAPQI catalyzed by MOF-818 was further analyzed. HPLC-MS results revealed that the abundant peak at *m/z* 301.12 corresponded to the deprotonated APAP dimer in a mixture of MOF-818 and APAP (Fig. S9a and b). Reportedly, the APAP dimer can be attacked by ·OH to produce NAPQI and reflected by generation of APAP-GSH additive product [27,28]. Consequently, a typical peak at *m/z* 457.14 corresponding to APAP-GSH was detected in the presence of MOF-818, APAP, and GSH (Fig. S9c–e), indicating the production of NAPQI (Figs 2j and S10). Encouragingly, the GSH levels of HCC1806 cells decreased more with APAP + MOF-818 treatment compared with MOF-818 treatment alone at 6 h (Fig. S6i), which was attributed to the multienzyme-mimicking activities and the generation of the APAP-GSH adduct by consuming GSH (Fig. S9e). Additionally, HCC1806 cells treated with APAP + MOF-818 displayed strong green fluorescence and significantly promoted ROS generation (Fig. S11), providing direct evidence of ROS accumulation.

In summary, we demonstrated that MOF-818 remodeled the TME by exerting multiple enzyme-like activities and triggered APAP to produce toxic products, thereby disrupting the balance between ROS and GSH in cancer cells.

### *In vitro* anti-cancer activity of MOF-818 by activating APAP

To visually monitor the endocytosis of MOF-818, AS1411 aptamer-modified fluorescent Ag nanoclusters (AgNCs-AS1411) with blue fluorescence [29] were loaded onto MOF-818 to construct AgNCs-AS1411@MOF-818 NPs. As shown in Fig. S12, the AgNCs-AS1411@MOF-818 NPs with a stable blue fluorescence signal were internalized by the HCC1806 CM, which was stained by DiO green fluorescence and reached a peak uptake at about 12 h, indicating the time-dependent internalization of MOF-818.

Viability assays revealed no significant toxicity in HCC1806 cells treated with 1 mM of APAP or 5 μg mL^−1^ of MOF-818 (Fig. S13). However, the concentration combination of APAP and MOF-818 dramatically suppressed cell proliferation in various cancer cell lines, including HCC1806, human cervical cancer cell line HeLa, human prostate cancer 22RV1 and PC3 cells, human neuroblastoma SH-SY5Y cells, human melanoma MNT1 cells, and rat pheochromocytoma PC12 cells (Fig. S14). Interestingly, no inhibitory influence of the APAP and MOF-818 combination treatment was observed in the human embryonic kidney cell line 293T (non-cancerous cells) (Fig. S14h).

To compare the anti-cancer efficacy of MOF-818 and the native tyrosinase-activated prodrug APAP, we cultured 293T and HCC1806 cells in phenol red-free medium and then evaluated cell proliferation. As shown in Fig. S15, under a reported tyrosinase dose of 30 μg mL^−1^ [30], 293T cells were completely killed, indicating its obvious toxicity. However, incubation with 3 μg mL^−1^ tyrosinase and APAP did not steadily inhibit HCC1806 cancer cell proliferation for 72 h. In comparison, the activation of prodrug APAP by MOF-818 significantly suppressed HCC1806 proliferation but barely influenced the growth of 293T cells (Figs S14h and S15a), and thus exhibited anti-cancer specificity and activity. Therefore, MOF-818 demonstrates effective antitumor activity while exhibiting minimal toxicity toward normal cells during prodrug activation.

### RNA-sequencing

**Figure 3.**
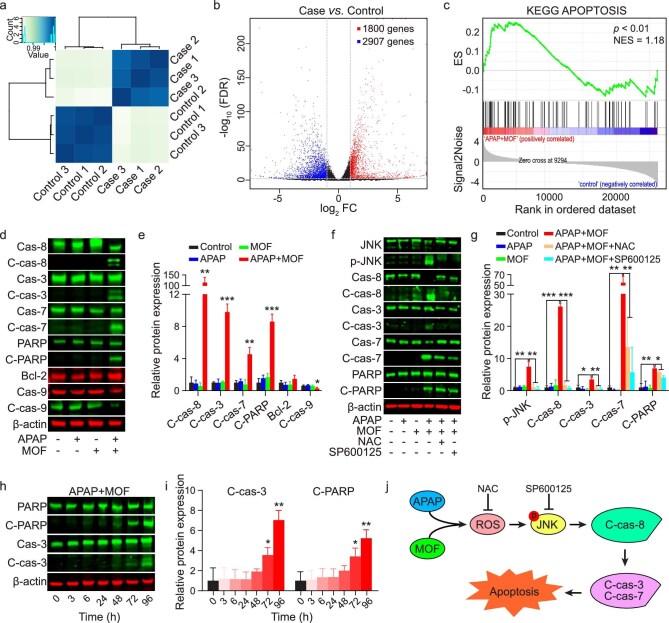
Molecular mechanism of cell death analysis and validation of apoptosis induced by MOF-818 and APAP. Pearson correlation analysis (a), volcano plot (b), and gene set enrichment analysis in apoptosis (c) from RNA-sequencing data of HCC1806 cells without treatment (Control) and with APAP + MOF-818 treatment (Case). (d, e) Western blot analysis of the apoptosis-associated proteins in HCC1806 cells after 48 h of treatments. (f, g) Western blot analysis of apoptosis- and JNK pathway-associated proteins in HCC1806 cells after 96 h of treatment. *N*-acetylcysteine (NAC) is a reactive oxygen species (ROS) scavenger and SP600125 is a JNK inhibitor. (h, i) Western blot analysis of the apoptosis-associated proteins in HCC1806 cells with MOF-818+APAP treatment for different times. (j) Schematic illustration of the possible mechanism of MOF-818 and APAP in promoting apoptosis. Cas-3/7/8/9, caspase 3/7/8/9; C-cas-3/7/8/9, cleaved caspase 3/7/8/9; C-PARP, cleaved PARP; FDR: false discovery rate; FC: fold change; KEGG: Kyoto encyclopedia of genes and genomes; ES: enrichment score; NES: normalized enrichment score; JNK: c-Jun N-terminal kinase; p-JNK: phosphorylated JNK. Data are given as mean ± SD (*n* = 3). *P < 0.05, **P  < 0.01, ***P  < 0.001.

To investigate the molecular mechanism of cell death induced by the MOF-818 activated prodrug APAP, RNA-sequencing analysis was used to reveal the transcriptome profile of HCC1806 cells with different treatments. A Pearson correlation analysis showed that the same treatment groups were significantly clustered, indicating high reliability of the data (Fig. 3a). There was a total of 4707 differentially expressed genes (DEGs) in the APAP + MOF-818 group, with 1800 upregulated and 2907 downregulated genes (Fig. 3b). Gene set enrichment analysis results showed that these DEGs were enriched in pathways such as apoptosis, glutathione metabolism, and cell cycle (Fig. 3c and Figs S16 and S17). Intriguingly, we also found that some disulfidptosis, cuproptosis, and autophagy-related key genes were significantly regulated in HCC1806 cells when treated with APAP and MOF-818 (Fig. S18).

### Co-triggering of apoptosis, disulfidptosis, and cuproptosis in cancer cells by MOF-818 and APAP

Apoptosis is divided into the death receptor pathway and the mitochondrial pathway, characteristically triggered by the pro-apoptosis proteins caspase 8 and caspase 9, respectively [31]. As shown in Fig. 3d and e and Fig. S19, several pivotal apoptosis proteins associated with the death receptor pathway, including cleaved caspase 3/7/8 and cleaved PARP, were significantly activated in HCC1806 cells. These marker proteins significantly increased over time in the presence of APAP and MOF-818 (Fig. 3h and i and Fig. S20). These results confirmed that apoptosis occurred *via* the death receptor pathway when stimulated by APAP and MOF-818. Moreover, inhibition of either ROS or p-JNK significantly inhibited apoptotic pathway activation using the ROS scavenger *N*-acetylcysteine (NAC) or the JNK inhibitor SP600125 (Fig. 3f and g and Fig. S21). Therefore, the activation of prodrug APAP by MOF-818 produced massive amounts of ROS, activated the phosphorylation-dependent JNK process, and finally induced apoptosis through the death receptor pathway in HCC1806 cells (Fig. 3j).

**Figure 4.**
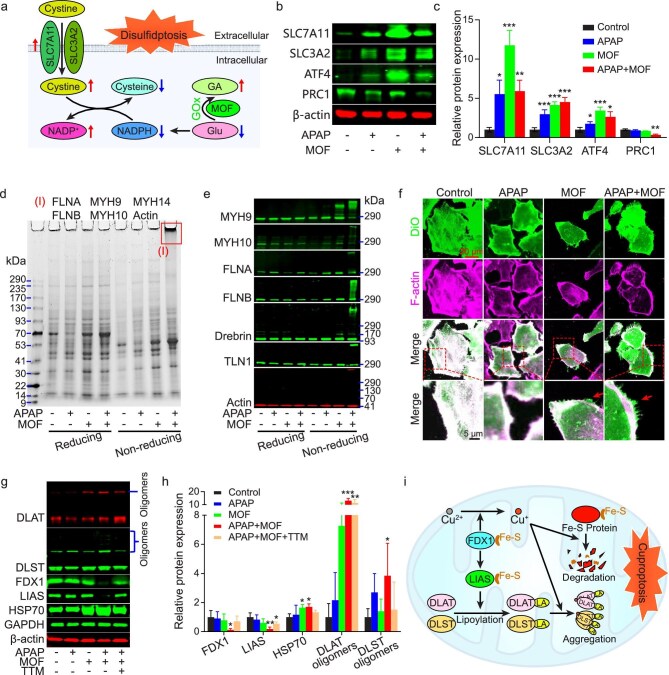
Verification of disulfidptosis and cuproptosis induced by the co-administration of MOF-818 and APAP in HCC1806 cells. (a) Schematic illustration of the possible mechanism of MOF-818 in promoting disulfidptosis. (b, c) Western blot analysis of the disulfidptosis-associated proteins. (d) Coomassie brilliant blue staining and the disulfidptosis-associated protein band identified by mass spectrometry (boxed region). (e) Reducing and non-reducing Western blot analysis of the disulfidptosis-associated proteins. (f) Fluorescent staining of F-actin and the cell membrane stained by Actin-Tracker Red-555 and DiO fluorescent probes, respectively. (g, h) Western blot analysis of the cuproptosis-associated proteins. (i) Schematic illustration of the possible mechanism promoting cuproptosis by MOF-818. Data are given as mean ± SD (c, h: *n* = 3). *P  < 0.05, **P  < 0.01, ***P  < 0.001.

Recently, a unique form of cell death, disulfidptosis, was reported [32]. Disulfidptosis relies on two essential factors in cancer cells [32]. One is SLC7A11 overexpression; the other is reduced NADPH in cells under glucose starvation (Fig. 4a). Western blot results showed that the combination treatment of APAP and MOF-818 increased SLC7A11 and SLC3A2 protein expression by 6.2- and 4.8-fold, respectively (Fig. 4b and c and Fig. S22), suggesting increased extracellular cystine and intracellular glutamate transport into and out of cells, respectively [33]. In addition, the expression of the disulfidptosis-positive and -negative factors ATF4 and PRC1 [34] was significantly upregulated and downregulated, respectively, in the APAP + MOF-818 group (Fig. 4b and c and Fig. S22). Next, we found that the intracellular glucose and NADPH levels in the cells treated with MOF-818 and APAP significantly decreased (Figs S6j and S23). Similar results were also obtained with the administration of MOF-818 alone, but the administration of APAP alone did not cause significant changes in glucose or NADPH levels (Figs S6j and S23). Therefore, intracellular glucose was consumed by MOF-818 through GOx-like activity, which depleted NADPH, hinting that MOF-818 and APAP treatment may promote disulfidptosis in cancer cells.

Then, we systematically investigated disulfidptosis. First, we found that the level of thiols significantly decreased in the MOF-818 group and the APAP + MOF-818 group compared with the control group, indicating the possibility of intracellular disulfide accumulation (Fig. S24). Next, Coomassie blue staining showed that the proteins extracted from the MOF-818 + APAP group migrated slowly and displayed extremely high molecular weight bands near the gel well under non-reducing conditions, but these proteins did not exhibit the same migration pattern under reducing conditions (Fig. 4d). The proteins near the well were identified by protein mass spectrometry analysis as actin cytoskeleton proteins, including myosin-9 (MYH9), MYH10, MYH14, actin, talin-1 (TLN1), Drebrin, and filamin-A and -B (FLNA/B). Moreover, Western blot analysis confirmed that the cytoskeletal proteins from the APAP + MOF-818 group formed aggregated proteins with large molecular weights under non-reducing conditions, but the bands disappeared under reducing conditions (Fig. 4e), suggesting that MOF-818 and APAP co-treatment induced mixed disulfide bonding among actin cytoskeleton proteins. Finally, as presented in Fig. 4f, striking changes in cell morphology were found in the MOF-818 and APAP + MOF-818 groups, characterized by cell shrinkage and F-actin contraction. Co-staining of F-actin (purple) and CMs (green) showed that MOF-818 caused F-actin to detach from the CM, and combined treatment with MOF-818 and APAP further strengthened this phenotype. Therefore, these results strongly suggested that combined MOF-818 and APAP treatment induced typical disulfidptosis in HCC1806 cells.

Cuproptosis is caused by copper-dependent mitochondrial dysfunction [35,36]. Therefore, JC-1 staining was performed to evaluate changes in the mitochondrial membrane potential. As shown in Fig. S25, the APAP + MOF-818 group showed stronger green fluorescence and weaker red fluorescence, leading to a significantly higher monomer/aggregate ratio. Importantly, the mitochondrial damage induced by APAP and MOF-818 was effectively attenuated by the copper chelator ammonium tetrathiomolybdate (TTM). This indicated that APAP coupled with MOF-818 induced mitochondrial damage associated with intracellular copper ion accumulation. Mechanistically, the mitochondrial damage caused by cuproptosis is closely related to the excessive oligomerization of lipoylated dihydrolipoamide S-acetyltransferase (DLAT) and dihydrolipoamide S-succinyltransferase (DLST), which are modified by ferredoxin 1 (FDX1) and lipoic acid synthetase (LIAS) to form aggregates in the presence of Cu^+^ [35]. Non-reducing Western blot analysis revealed that the combination of APAP and MOF-818 induced excessive oligomerization of DLAT and DLST proteins, increased the level of HSP70, and reduced the expression FDX1 and LIAS (Fig. 4g and h and Fig. S26), reflecting acute proteotoxic stress and mitochondrial functional damage. These suggested that significant proteotoxic stress and cell death occurred. Crucially, all changes in the cuproptosis-associated factors were partially rescued by TTM (Fig. 4g–i and Fig. S26).

To further determine which pathway predominantly contributes to cell death in cancer cells treated with APAP and MOF-818, HCC1806 cells were pretreated with each individual inhibitor (Z-VAD-FMK, Tris(2-carboxyethyl)phosphine hydrochloride (TCEP), and TTM for apoptosis, disulfidptosis, and cuproptosis, respectively) [32,35,37] or with a combination of all three, followed by co-incubation with the MOF-818 + APAP system for 24, 48, and 72 h. The results demonstrated that apoptosis played an important role throughout the entire process of cell death, disulfidptosis contributed substantially during the intermediate stage, and cuproptosis exerted its cytotoxic effect mainly in the late stage (Fig. S27). Notably, co-treatment of all three inhibitors resulted in the greatest recovery of cell viability, further confirming that apoptosis, disulfidptosis, and cuproptosis act in a cooperative rather than independent manner.

Altogether, our results demonstrated that MOF-818 combined with APAP promoted apoptosis, disulfidptosis, and cuproptosis in cancer cells.

### Design and preparation of 3MCT NPs

In view of the need for precise tumor treatment, we then designed a strategy for *in vivo* tumor targeting treatment. Interestingly, we found that MOF-818 and APAP upregulated expression of the cell death receptors DR4 and DR5, and this upregulation was inhibited by inhibitors of ROS and JNK (Fig. S28), suggesting that intracellular ROS levels play a role in enhancing DR4/5 cluster. Reportedly, by binding to DR4/5, a member of the TNF superfamily of ligands, TRAIL, was shown to selectively induce apoptosis in cancer cells [38]. Although TRAIL has been recognized as a promising anti-cancer drug, traditional soluble TRAIL (sTRAIL) has ineluctable shortcomings, such as low stability and short half-life [39]. Thus, we developed an sTRAIL mutant (msTRAIL, R121A) to significantly enhance its soluble expression efficiency in *Escherichia coli*, while preserving its classic activity (Fig. S29). Then, the fusion protein Fc-TRAIL (FT) was constructed with the Fc region of human IgG1 as the N-terminus, a linker of five flexible amino acids (GGGGS) in the middle, and msTRAIL in the C-terminus (Figs S29a and S30). As shown in Fig. S29a, a structure prediction based on deep learning indicated that the Fc fragment and msTRAIL in the fusion protein may maintain relative spatial and structural independence. Experimentally, the purified FT protein was recognized by anti-TRAIL and anti-human IgG antibodies (Fig. S31), suggesting that FT was successfully recombined. As expected, the FT protein retained the classical biological function of TRAIL, effectively inducing apoptotic cell death in HCC1806 cells and in human large cell lung cancer NCI-H460 cells, but did not affect activity in normal cells, including mammary epithelial cells MCF10A, 293T cells, and prostate epithelial cells RWPE-1 (Figs S29b and S32). Considering that the CH2 and CH3 domains of the Fc portion can recognize and bind to Fc receptors (FcγRI and FcγRII) on the membrane of THP-1 cells [40], we encapsulated MOF-818 using THP-1 CMs, and then effectively loaded FT protein onto the membrane through ligand-receptor binding, thereby establishing a ligand-displaying nanoplatform.

Autophagy is inevitable in cancer cells with sufficient ROS accumulation [41]. Similar to the results of gene expression (Fig. S18c), Western blot results confirmed that the expression levels of autophagy-related proteins were significantly upregulated in HCC1806 cells treated with APAP and MOF-818, including Beclin-1 (encoded by the *BECN1* gene), LC3B-II/LC3B-I (*MAP1LC3B*) ratio, Atg3, Atg5, Atg7, Atg12, Atg16L1, and P62 (*SQSTM1*) (Figs S33a and b and S34). Of note, NAC and SP600125 significantly reduced the expression of autophagy-related proteins (Figs S33a and b and S34), further confirming that autophagy was caused by the enhanced phosphorylation of JNK induced by ROS in this case. Moreover, incubation with APAP and MOF-818 prompted autolysosomal formation (free red dots) in HCC1806 cells, indicating that autophagy flow was significantly activated (Fig. S33c–e). We then confirmed that 3-MA (an autophagy inhibitor) significantly reduced the cellular activity of HCC1806 cells after activation of the prodrug APAP by MOF-818 (Fig. S35). This may be attributed to the inhibition by 3-MA of the protective mechanism generated through autophagy activation in cancer cells. Therefore, 3-MA of negative charges was loaded on MOF-818 of positive charges through electrostatic adsorption to create 3-MA@MOF-818 (Fig. S36) to inhibit protective autophagy and enhance the growth suppression of cancer cells.

**Figure 5.**
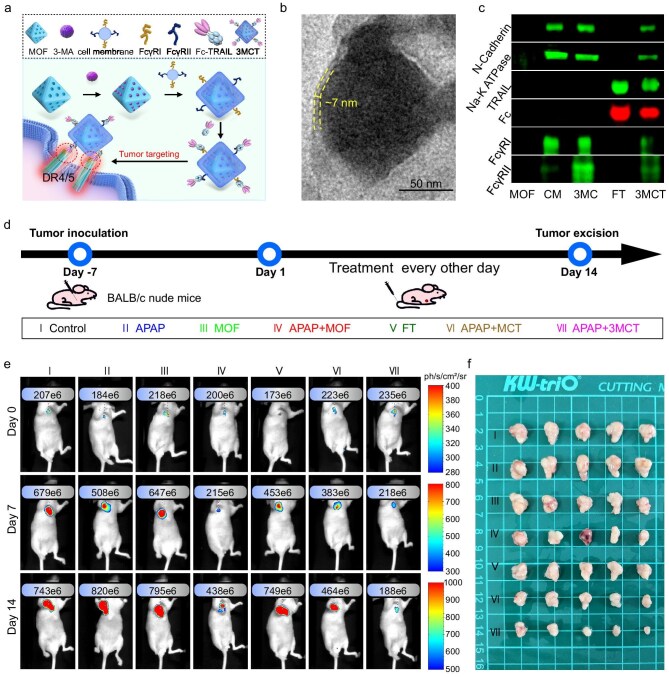
The 3-MA@MOF-818@CM-Fc-TRAIL nanoparticles (3MCT NPs) preparation and tumor-targeting therapy of 3MCT NPs with APAP *in vivo*. (a) Process diagram of the 3MCT NPs preparation and schematic illustration of 3MCT NPs targeting DR4 and DR5 receptors on tumor cell membrane surfaces. (b) TEM image of the 3MCT NP. The dashed line indicates the cell membrane coating the surface of MOF-818. (c) The successful preparation of 3MCT NPs verified by Western blot assay using different antibodies. (d) Treatment schedule for GFP-labeled HCC1806-bearing mice. (e) Changes in the fluorescence intensity in xenograft tumors on days 0, 7, and 14. (f) Image of isolated tumors at the end of treatment. Groups I to VII are control, APAP, MOF-818, APAP + MOF-818, FT, APAP + MCT, and APAP + 3MCT treatments, respectively.

Finally, we prepared 3-MA@MOF-818@CM-FT (3MCT) NPs using 3-MA@MOF-818, THP-1 CMs, and the fusion protein FT (Fig. 5a). The TEM image of 3MCT NPs showed that the THP-1 CM was successfully coated on the surface of 3-MA@MOF-818 (Fig. 5b and Fig. S37). In addition, the proteins of 3MCT NPs were specifically recognized by antibodies against membrane proteins (N-cadherin, Na-K ATPase, FcγRI, and FcγRII) and FT (Fig. 5c). These results suggested 3MCT NPs had been successfully prepared. As expected, HCC1806 cells treated with APAP and 3MCT showed a significantly increased intracellular ROS abundance and decreased GSH content compared with the control group (Fig. S38a–c). Interestingly, compared to APAP + MOF-818 treatment, APAP + 3MCT treatment further promoted apoptosis and inhibited cell proliferation in cancer cells (Fig. S38d–f).

To evaluate the stability of 3MCT NPs under physiological conditions, we performed *in vitro* dynamic simulations involving incubation at 0.83 s^−1^ and 37°C in normal saline or saline supplemented with 10% fetal bovine serum (FBS), following a reported method [42]. After 24 h of incubation, 3MCT NPs showed moderate changes in morphology (Fig. S39a–c), particle size (Fig. S39d–f), and structural integrity (Fig. S39g–i), while they maintain essential colloidal stability without aggregation (Fig. S39j). Furthermore, HPLC-MS analysis demonstrated a low release (<20%) of the 3-MA from 3MCT NPs (Fig. S39k). Notably, ELISA assays indicated a significantly slower release rate of the surface Fc-TRAIL from 3MCT in the presence of FBS (Fig. S39l), which may be ascribed to the steric stabilization provided by the protein corona [43]. Consequently, these results indicate that the 3MCT NPs exhibit notable stability under physiological conditions, along with favorable drug delivery capabilities.

To investigate the tumor-targeting capacity of 3MCT NPs *in vivo*, we used DiD dye to label the MOF-818 and 3MCT NPs and examined their biodistribution in HCC1806 tumor-bearing BALB/c nude mice at different time points after tail vein injection. The fluorescence signals of both MOF‑818 and 3MCT NPs peaked at the tumor site at 12 h post‑injection and remained strong even after 72 h (Fig. S40a and b). Compared to MOF-818, 3MCT NPs exhibited stronger and more sustained tumor accumulation (Fig. S40b), which was further supported by *ex vivo* imaging of harvested organs (Fig. S40c). These results suggested that the FT fusion protein on the 3MCT surface guided the nanoparticles to the tumor tissue *in vivo* and promoted their prolonged retention in the tumor.

### *In vivo* tumor-targeting therapy of 3MCT NPs with APAP

Given the effective tumor targeting and pronounced *in vitro* treatment effect of 3MCT NPs, we performed *in vivo* tumor-targeting treatment using HCC1806 cells with stable expression of green fluorescence protein (GFP-HCC1806) to generate GFP-HCC1806 tumor-bearing mice. A total of 35 tumor-bearing mice were randomly divided into seven groups: control, APAP, MOF-818, APAP + MOF-818, FT, APAP + MCT, and APAP + 3MCT to receive the corresponding drug treatments (Fig. 5d). As shown in Fig. 5e, live imaging showed that the cancer cells were minimally suppressed in mice administered APAP, MOF-818, or FT alone compared with the control group. The APAP + MOF-818 and APAP + MCT groups showed attenuated cancer cell growth inhibition. In contrast, the cancer cell growth in the APAP + 3MCT group was significantly inhibited. Similarly, all tumors harvested from the APAP + 3MCT group were remarkably smaller than those of the other groups, indicating synergistic effects from the TME reprogramming of APAP + MOF-818 treatment, tumor-targeting of FT, and autophagy inhibition of 3-MA (Fig. 5f and Fig. S41a and b). Importantly, the body weights of mice in all seven groups showed a similar increasing trend, confirming the safety of the treatments *in vivo* (Fig. S41c).

Immunohistochemistry staining of tumor tissue against HIF-1α, a typical hypoxia marker [44], revealed that the tumor tissue in the MOF-818 group exhibited only weak signals, due to MOF-818 relieving hypoxic stress in the TME, suppressing HIF-1α expression (Fig. S42a and b). Moreover, *in situ* hybridization with a ROS fluorescent probe revealed that the tumor tissue from the APAP + MOF-818, APAP + MCT, and APAP + 3MCT groups significantly accumulated ROS compared to tumor tissue from the control group (Fig. S42a and c). As expected, immunofluorescent staining results showed that DR4/5 proteins were significantly upregulated in these groups (Fig. S42a, d and e) due to ROS accumulation. Consequently, the APAP + 3MCT group showed the highest apoptotic index and the lowest level of cell proliferation, as evaluated by TUNEL and Ki67 immunofluorescence staining, respectively (Fig. S42a, f and g). Notably, a series of processes, including the endocytosis of MOF-818 into tumor cells (A–C); the formation of phagophores (D), autophagosomes (E, black arrows), and autolysosomes (E, yellow arrows); and apoptosis (F), was also verified by TEM in tumor tissue (Fig. S42h).

These results suggested that the 3MCT NPs in combination with APAP hold promise for effective anti-tumor strategies.

### *In vivo* biosafety and metabolism evaluation of 3MCT NPs

Drug biosafety and metabolism are prerequisites for drug applications. Considering that the usage dose of APAP in this study was within the *in vivo* safe range [45,46], we focused on evaluating the biosafety, bio-distribution, and metabolism of the nanomaterials. The hemolysis rates of MOF-818 and 3MCT NPs were less than 5% at 100 μg mL^−1^ and 200 μg mL^−1^ concentrations, respectively (Fig. S43). Moreover, the mice group injected with MOF-818 for 30 days did not show significant weight loss compared with the control group (Fig. S44), indicating that MOF-818 had no significant systemic toxicity to mice after long-term administration. In tumor-bearing mice for the different treatments (Fig. 5d), hematoxylin and eosin (H&E) staining showed that there were no obvious pathological changes in the main organs (Fig. S45). In addition, the liver and kidney function indexes and the routine blood indexes in the treatment group did not deviate from the normal range (Figs S46 and S47).

The *in vivo* distribution of MOF-818, quantified by inductively coupled plasma mass spectrometry technology (ICP-MS) for Cu and Zr elements, demonstrated that MOF-818 was primarily accumulated in the liver and spleen after daily subcutaneous administration for 30 days (Fig. S44c and d). Similarly, 3MCT NPs mainly concentrated in the liver and spleen in BALB/c nude mice following tail vein injection (Fig. S48).

The results of MOF-818 metabolism showed that Cu and Zr in the blood, feces, and urine were almost completely degraded by 72 h (Fig. S49), suggesting that MOF-818 was sufficiently metabolized *in vivo*. Additionally, we also compared the FT stability in blood between FT and 3MCT NPs administered to BALB/c mice *via* tail vein injection. The results showed that free FT remained stable in the blood for 3 h, then slowly decreased, and was approximately half of the original concentration at 24 h (Fig. S50). Encouragingly, the concentration of FT loaded in 3MCT NPs only decreased slightly at 24 h (Fig. S50), indicating that the 3MCT NP delivery system effectively maintained FT activity.

In conclusion, the excellent biocompatibility and metabolism of 3MCT NPs provide assurance for *in vivo* applications.

## DISCUSSION

Exploring a single nanozyme with multienzyme-mimicking activities for enzymatic-boosted tumor therapy is imperative [8]. MOF-based cascade catalytic tumor therapy has shown huge potential [47]. Therefore, we developed the single nanozyme MOF-818 with bimetallic Cu-Zr exhibiting six enzyme-mimicking activities. For the first time, we proposed that MOF-818 showed efficient GOx, GSHOx, and TYR-like activities, offering the possibility for cancer therapy by starving cancer cells, disrupting cellular redox homeostasis, and incorporating prodrug adjuvant treatment, respectively. To the best of our knowledge, this is the first finding of a nanozyme with TYR-like activity. Using its TYR-like activity, the MOF-818 catalyzed the prodrug APAP to produce highly toxic AOBQ, and together with NAPQI produced by ·OH attacking the APAP dimer, significantly promoted GSH consumption and ROS accumulation, further disrupting redox homeostasis. Here, we revealed that the high SOD-mimicking activity of MOF-818 was important for promoting the cyclic catalytic reaction process by sustaining H_2_O_2_ production. Considering that MOF-818 catalyzed the entire process, from self-generated substrates and cyclic catalytic reactions (Fig. 2h), we named the process as a novel RCC reaction, distinct from a cascade catalytic reaction. Therefore, through the RCC reaction and prodrug activation, MOF-818 remodeled the TME, effectively disrupted the balance between ROS and GSH in cancer cells and produced toxic products.

Co-triggering various cell death pathways has emerged as an innovative approach in tumor therapy. In our case, we found that MOF-818 coupled with APAP triggered apoptosis, disulfidptosis, and cuproptosis to induce cell death, functioning as a ‘three-in-one’ effect. Disulfidptosis and cuproptosis have recently been reported as novel programmed cell death (PCD) therapies. Thus, most nanobiomedical drugs have been designed to trigger the onset and progression of these PCD pathways *via* loading natural enzymes or small molecules, such as GOx and digitonin [48,49]. Importantly, due to MOF-818 copper-bearing characteristics and CAT, GOx, GSHOx, and SOD-like activities, MOF-818 effectively induced a surge in copper ions, decreased glucose and GSH concentrations, and increased the oxygen level in cancer cells. These functions paved the way for cuproptosis and disulfidptosis to proceed smoothly.

**Figure 6.**
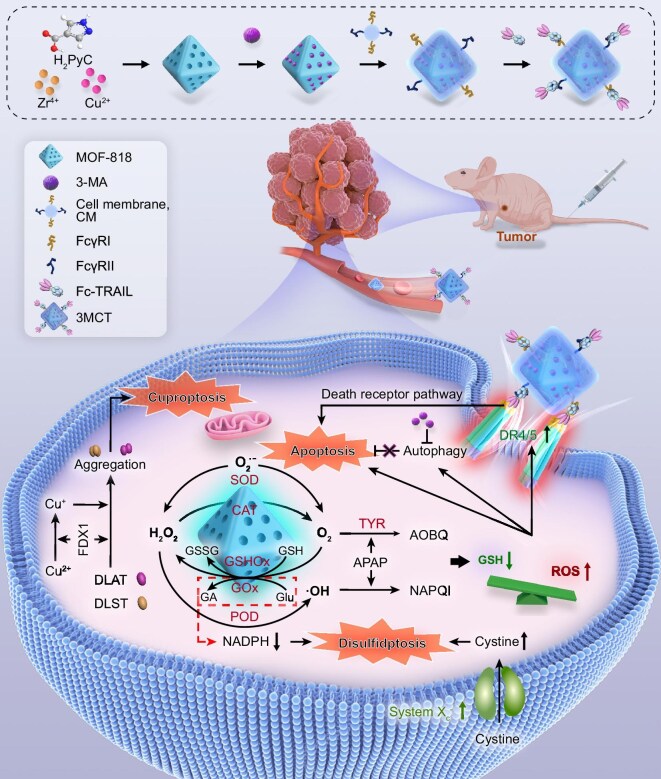
Schematic illustration of the anti-neoplastic function of 3MCT NPs coupled with APAP. 3MCT NPs are effectively taken up by targeting TRAIL receptors DR4 and DR5 on the surface of tumor cells. 3MCT NPs initiate rolling circle catalysis and activate the prodrug APAP, triggering apoptosis, disulfidptosis, and cuproptosis in cancer cells.

The remarkable high performance of nanoplatforms is fundamental for nanodrug engineering. We developed a novel and promising strategy for tumor-targeting 3MCT NPs based on the MOF-818 nanoplatform (Fig. 6). The superiority of this system can be described as follows: (1) FT killed two birds with one stone. The FT protein selectively suppressed growth in different cancer cell lines and specifically bound nanomaterials to the membrane of cancer cells as a bridge. (2) The membrane used as a nanomaterial coating protected the integrity of the nanodrug and maintained the catalytic activity of the nanozyme. (3) FT loaded on 3MCT NPs further prolonged the short half-life of TRAIL under physiological conditions without any significant side effects. FT effectively triggered proapoptotic death receptor-mediated apoptosis in cancer cells. Simultaneously, the nanozymes *per se* also reprogrammed the TME and activated diverse cell death pathways. As a result, the combination of 3MCT NPs and APAP induced multiple cell death mechanisms, significantly enhancing the efficacy of tumor treatment. Given the molecular composition (phospholipid bilayer), biological origin (homology), and administration route (intravenous injection) of THP-1 CMs [50–52], 3MCT NPs may possess low immunogenicity and favorable translational feasibility for human cancer therapy. Therefore, MOF-818 represents a promising nanoplatform with potential strategies for cancer therapy and related biotechnological fields.

## CONCLUSION

Taken together, we report an efficient MOF-818 with six enzyme-like activities. To the best of our knowledge, this is the first report of a nanozyme with TYR-like activity. Based on these broad enzymatic activities, MOF-818 significantly reshaped the TME by increasing ROS and O_2_, decreasing GSH, and consuming glucose by the RCC reaction process in cancer cells. The 3MCT NPs coupled with APAP achieved a good therapeutic efficacy by self-generating H_2_O_2_ in a cyclic manner, blocking the glucose supply, and relieving hypoxia, thereby causing a large amount of ROS production. Consequently, the co-administration of 3MCT NPs and APAP induced apoptosis, disulfidptosis, and cuproptosis to achieve an efficient tumor-targeting treatment. This versatile nanoplatform and tumor-targeting system holds promise for effective anti-tumor strategies.

## MATERIALS AND METHODS

### Cell culture and treatment

All cell lines were obtained from the Conservation Genetics CAS Kunming Cell Bank (Kunming, China). GFP-HCC1806 cells were obtained from Gene Pharma (Shanghai, China). HeLa cells, 293T cells, and PC12 cells were cultured in DMEM supplemented with 10% FBS, 100 U mL^−1^ penicillin and 0.1 mg mL^−1^ streptomycin. As cultured for 22RV1 cells and HCC1806 cells as reported in previous studies [53,54], PC3 cells, THP-1 cells and NCI-H460 cells were grown in RPMI 1640 supplemented with 10% FBS and 100 U mL^−1^ penicillin and 0.1 mg mL^−1^ streptomycin. MNT1 cells were cultured in DMEM supplemented with 10% FBS, 100 U mL^−1^ penicillin and 0.1 mg mL^−1^ streptomycin, and 1% non-essential amino acids. SH-SY5Y cells were cultured in DMEM/F12 supplemented with 15% FBS, 100 U mL^−1^ penicillin, and 0.1 mg mL^−1^ streptomycin. All these cells were cultured at 37°C in a humidified atmosphere with 5% CO_2_. Cells were treated with APAP (1 mM), MOF-818 (5 μg mL^−1^), 3-MA@MOF-818@CM-FT (3MCT, 5 mM 3-MA, 5 μg mL^−1^ MOF-818, and 100 ng mL^−1^ FT), NAC (5 mM), SP600125 (10 μM), Z-VAD-FMK (20 μM), TCEP (1 mM) or TTM (10 μM).

### Synthesis of MOF-818

MOF-818 was synthesized according to reported studies with some modifications [11,13,14]. ZrOCl_2_·8H_2_O (31.88 mg), Cu(NO_3_)_2_·3H_2_O (9 mg), and H_2_PyC (24.38 mg) were dissolved in DMF by ultrasonication. Then, 120 μL of trifluoroacetic acid (TFA) was added to the solution and dispersed by ultrasonication. The mixture was transferred into a Teflon-lined autoclave and heated at 100°C for 4 h. Next, additional Cu(NO_3_)_2_·3H_2_O (84 mg) was added to the solution and dissolved by ultrasonication. The mixture was heated to 100°C for another 10 h. After cooling, the blue crystals were soaked in DMF followed by acetone for three days (three times each day). Finally, MOF-818 was vacuum dried at 60°C for 12 h.

### 3MCT nanoparticle preparation

The CMs were extracted from THP-1 cells using a Minute™ plasma membrane protein isolation and cell fractionation kit (Invent Biotechnologies, Inc., MN, USA). Using electrostatic adsorption, 3-MA was first incubated with MOF-818 at a ratio of 1:12.5 at room temperature with stirring for 3 h to prepare 3-MA@MOF-818 (3M). Next, the CMs extracted from 1 × 10^7^ THP-1 cells were combined with 6.5 mg of 3M by ultrasonication in an ice bath for 3 min (on for 5 s, off for 5 s) to prepare 3-MA@MOF-818@CM (3MC). Then, the Fc-TRAIL (FT) protein was incubated with 3MC at 4°C for 6 h to prepare 3-MA@MOF-818@CM-Fc-TRAIL (3MCT). Finally, the mixture was centrifuged at 8000 g for 3 min, washed three times with PBS (pH 7.2), and stored at 4°C for *in vivo* experiments.

### Animal experiments

All animal experimental protocols were approved by the Institutional Animal Care and Use Committee, Yunnan University (approval No. YNU20230523).

## Supplementary Material

nwag316_Supplemental_Files

## Data Availability

The data that support the findings of this study are available from the corresponding author upon reasonable request. The sequencing data that support the findings of this study have been deposited in the Sequence Read Archive (SRA) (http://www.ncbi.nlm.nih.gov/sra) with the BioProject accession code PRJNA1199234.
