# Supplementary material for: A multienzyme-mimicking nanoplatform induces disulfidptosis/cuproptosis/apoptosis for tumor therapy
Source: Natl Sci Rev. 2026 May 27;13(12):nwag316. doi: 10.1093/nsr/nwag316 (PMC13317449; doi:10.1093/nsr/nwag316)
Supplement: nwag316_Supplemental_Files [file nwag316_supplemental_files.zip › Supplementary Material 1.pdf]

Supporting Information

A multienzyme-mimicking nanoplatform induces disulfidptosis/cuproptosis/apoptosis for tumor therapy

Wei-Jie Sun[1,2,3,†], Jie Lin[1,†], Xiao-Kang Lu[4,†], Jin Fu[5], Jia-Wen Li[1], Hong-Min Zhu[4], Feng-Qi Zhou[4], Si-Ying Ma[1], Guo-Bao Ning[4], Bi-Lian Li[4], Hang Chen[4], Jie Li[2,3], Yu-Xun Lu[4], Xiang Lai[4], Lian Jin[1], Bai-Cheng Lu[1], Can-Peng Li[1,*], Ya-Ping Zhang[1,2,*] & Hui Zhao[1,*]

[1] Bio-X Center for Interdisciplinary Innovation, Yunnan University, Kunming 650500, China

[2] State Key Laboratory of Genetic Evolution & Animal Models, Kunming Institute of Zoology, Chinese Academy of Sciences, Kunming 650201, China

[3] Kunming College of Life Science, University of the Chinese Academy of Sciences, Kunming 650204, China

[4] School of Chemical Science and Technology, Yunnan University, Kunming 650500, China

[5] Pathology department, the Second People's Hospital of Yunnan Province, Kunming, 650021, China

†Contributed equally to this work

*Corresponding author (Hui Zhao, email: zhaohui@ynu.edu.cn; Ya-Ping Zhang, email: zhangyp@mail.kiz.ac.cn; Can-Peng Li, email: lcppp1974@sina.com)

METHODS

Materials and Reagents

Zirconyl chloride octahydrate ($ZrOCl_2 \cdot 8H_2O$), copper nitrate trihydrate ($Cu(NO_3)_2 \cdot 3H_2O$), and 1H-pyrazole-4-carboxylic acid (H_2PyC) were purchased from Adamas-beta (Shanghai, China). Trifluoroacetic Acid (TFA), L-tyrosine, reduced glutathione (GSH), doxorubicin (DOX), silver nitrate ($AgNO_3$), sodium borohydride ($NaBH_4$), hydrogen peroxide (H_2O_2, 30%) and 3,3′,5,5′-Tetramethylbenzidine (TMB) were purchased from Aladdin (Shanghai, China). N,N-dimethylformamide (DMF) was purchased from Beijing Chemical Works (Beijing, China). Acetaminophen (APAP), N-Acetyl-L-cysteine (NAC), SP600125, HKSOX-1r, HKPerox-2, HKOH-1r, DiO, DiD, cisplatin, paclitaxel, camptothecin, Z-VAD-FMK (Z-VAD), Tris(2-carboxyethyl)phosphine hydrochloride (TCEP) and ammonium tetrathiomolybdate (TTM) were purchased from MedChem Express (NJ, USA). Phenol was purchased from Macklin Reagent (Shanghai, China). L-dopa and RPMI 1640 (phenol red-free) were purchased from Solarbio (Beijing, China). AS1411 aptamer was synthesized by Tsingke Biotechnology Co., Ltd. (Beijing, China). DCFDA/H2DCFDA-Cellular Reactive Oxygen Species (ROS) Assay Kit was purchased from Abcam (Cambridge, UK). Enhanced mitochondrial membrane potential assay kit, Actin-Tracker Red-555, cell counting kit-8 (CCK-8), AdPlus-mCherry-GFP-LC3B kit, and Annexin V-FITC/PI Apoptosis Kit were purchased from Beyotime Biotechnology (Shanghai, China). Antibodies against caspase 8, cleaved caspase 8, caspase 3, cleaved caspase 3, caspase 7, cleaved caspase 7, PARP, cleaved PARP, Bcl-2, caspase 9, cleaved caspase 9, JNK, p-JNK, LC3B, Beclin-1, Atg3, Atg3, Atg5, Atg7, Atg12, Atg16L1, p62, DR4, DR5, TRAIL, ATF4, and TLN1 for Western blot were purchased from Cell Signaling Technology (MA, USA). Antibodies against N-Cadherin, Na-K ATPase, FcγRI,

FcγRII, SLC7A11, SLC3A2, PRC1, and GAPDH for Western blot were purchased from Abcam (Cambridge, UK). Antibodies against MYH10, FLNA, FLNB, and Drebrin for Western blot were purchased from Affinity Biosciences (Changzhou, China). Antibodies against MYH9, DLAT, and DLST for Western blot were purchased from MedChem Express (NJ, USA). Antibodies against FDX1 and LIAS for Western blot were purchased from Proteintech (Wuhan, China). Antibody against HSP70 for Western blot was purchased from Boster (Wuhan, China). Antibody against actin for Western blot was purchased from Thermo Fisher Scientific (MA, USA). Labeling antibodies with fluorescence tags of IRDye® 800CW goat anti-rabbit IgG and IRDye® 680RD goat anti-mouse IgG were purchased from LI-COR (NE, USA). Antibody against HIF-1α for immunohistochemistry was purchased from Abcam (Cambridge, UK). Antibodies against DR4 and DR5 for immunohistochemical fluorescence were purchased from Cell Signaling Technology (MA, USA). Antibody against Ki67 for immunohistochemical fluorescence was purchased from Abcam (Cambridge, UK). TUNEL Apoptosis Detection Kit was purchased from Boster (Wuhan, China). Dihydroethidium (DHE) for ROS *in situ* detection was purchased from Sigma-Aldrich (Buchs, Switzerland). Human TRAIL/TNFSF10 Quantikine ELISA Kit was purchased from R&D Systems (MN, USA). Phosphate buffered saline (PBS, pH 7.2) and penicillin-streptomycin were purchased from Basal Media (Shanghai, China). The culture mediums of RPMI 1640, DMEM, and DMEM/F12, and fetal bovine serum (FBS) were purchased from Gibco (Grand Island, NY, USA).

Synthesis of MOF-818

MOF-818 was synthesized according to reported studies with some modifications [1,2,3]. $ZrOCl_2 \cdot 8H_2O$ (31.88 mg), $Cu(NO_3)_2 \cdot 3H_2O$ (9 mg), and H_2PyC (24.38 mg)

were dissolved in DMF by ultrasonication. Then, 120 μL of TFA was added to the solution and dispersed by ultrasonication. The mixture was transferred into a Teflon-lined autoclave and heated at 100°C for 4 h. Next, additional $Cu(NO_3)_2 \cdot 3H_2O$ (84 mg) was added to the solution and dissolved by ultrasonication. The mixture was heated to 100°C for another 10 h. After cooling, the blue crystals were soaked in DMF followed by acetone for three days (three times each day). Finally, MOF-818 was vacuum dried at 60°C for 12 h.

The preparation of Fc-TRAIL fusion protein

To obtain recombinant Fc-TRAIL (FT), a pET/Fc-msTRAIL plasmid was constructed. In detail, the human *IgG1* Fc gene (GenBank: MG920247.1) was cloned into pUC57 by Tsingke Biotechnolgy (Beijing, China). Then, a human *IgG1* Fc DNA fragment with the hinge, CH2, and CH3 domains was synthesized. The coding sequence of the G_4S linker was introduced at the 3'-terminus by PCR amplification, and the fused gene fragment (Fc-linker) was cloned into our previously constructed pET/msTRAIL vector at the N-terminus of msTRAIL to generate a pET/Fc-msTRAIL plasmid. pET/Fc-msTRAIL was then transformed into *E. coli* BL21(DE3) to obtain engineered bacteria that could express our designed Fc-TRAIL (FT) protein under the control of a T7 promoter. The sequences of primer pairs used in the plasmid construction process were as followed: 5'- AAC CAT GGG CGA ACC GAA ATC TTG TGA T-3' (forward) and 5'- GGA TCC ACC GCC ACC TTT ACC CGG GCT CAG GCT C-3' (reverse). To improve the soluble expression levels in prokaryotic cells, our target protein FT was expressed by inducing the cells using a low concentration of IPTG (0.1 mM) at a reduced temperature (14 °C). After cells were lysed under non-denaturing conditions, the target fusion protein was purified to homogeneity from the clarified extract using

nickel affinity chromatography (Histrap™ HP, Cytiva, MA, USA) and protein A affinity chromatography (Hitrap™ protein A HP, Cytiva, MA, USA).

3MCT nanoparticle preparation

The CMs were extracted from THP-1 cells using a Minute™ plasma membrane protein isolation and cell fractionation kit (Invent Biotechnologies, Inc., MN, USA). Using electrostatic adsorption, 3-MA was first incubated with MOF-818 at a ratio of 1:12.5 at room temperature with stirring for 3 h to prepare 3-MA@MOF-818 (3M). Next, the CMs extracted from 1×10^7 THP-1 cells were combined with 6.5 mg of 3M by ultrasonication in an ice bath for 3 min (on for 5 s, off for 5 s) to prepare 3-MA@MOF-818@CM (3MC). Then, the Fc-TRAIL (FT) protein was incubated with 3MC at 4°C for 6 h to prepare 3-MA@MOF-818@CM-Fc-TRAIL (3MCT). Finally, the mixture was centrifuged at 8000 g for 3 min, washed three times with PBS (pH 7.2), and stored at 4°C for *in vivo* experiments.

Characterizations

The morphology of nanoparticles (NPs) was measured using a JEM-F200 transmission electron microscope (TEM, JEOL, Tokyo, Japan). X-ray diffraction (XRD) curve was acquired by using a TTRIII-18KW diffractometer instrument (Rigaku, Tokyo, Japan) in the 2θ range from 1° to 50°. Fourier transform infrared (FT-IR) spectroscopy of MOF-818 was recorded on a Nicolet IS10 (Thermo Fisher Scientific, MA, USA). X-ray photoelectron spectroscopy (XPS) was conducted on ESCALAB Xi+ spectrometer (Thermo Fisher Scientific, MA, USA). N_2 adsorption-desorption isotherms were measured using ASAP 2020M automated sorption analyzer (Micromeritics, GA, USA). Absorption spectra were acquired by

using BioTek Epoch microplate spectrophotometer (VT, USA) or UNICO UV-2800A UV/VIS spectrophotometer (Shanghai, China). The concentrations of Cu and Zr were quantitatively investigated by inductively coupled plasma mass spectrometry (ICP-MS; 7500CE, Agilent, CA, USA). Dynamic light scattering (DLS) and zeta potential value measurements were performed using NanoBrook 90Plus PALS (Brookhaven Instruments Corporation, MD, USA). The structure of the fusion protein Fc-TRAIL (FT) was predicted using RoseTTaFold on Robetta service (https://robetta.bakerlab.org/submit.php), followed by visualization in PyMOL (v 3.1.0) to elucidate structural features and facilitate further analysis.

Enzyme-like activities determination of MOF-818

The SOD-mimic activity of the synthesized MOF-818 was assessed using an SOD assay kit (WST-8). This assay is designed to measure the ability of substances to scavenge $O_2^{\cdot-}$, which was generated during the oxidation of xanthine. These $O_2^{\cdot-}$ then reacted with WST-8 reagent, forming a formazan dye that shows a characteristic absorption peak at 450 nm. As SOD consumes $O_2^{\cdot-}$, the absorbance at 450 nm decreases, and the inhibition rate of $O_2^{\cdot-}$ generation correlates with the SOD activity. Different concentrations of nanozymes and substrate were incubated, and the absorbance changes of WST-8 at 450 nm were collected to calculate SOD-like activity based on inhibition rate.

The CAT-like activity of MOF-818 was determined by monitoring the amount of O_2 produced at a constant concentration of H_2O_2 (20mM). MOF-818 (0, 2.5, 5, and 10 μg mL^{-1}) was mixed with H_2O_2 in PBS and the dissolved O_2 concentration was monitored within 5 min using a dissolved oxygen meter.

The POD-like activity of MOF-818 was evaluated through a colorimetric assay

utilizing 3,3',5,5'-tetramethylbenzidine (TMB). Different concentrations of TMB, H_2O_2, along with 125 μg mL^{-1} MOF-818 were dispersed in HAc-NaAc buffer solution (pH 4.5). The UV visible absorption spectrum was subsequently recorded at the wavelength of 652 nm [4].

The tyrosinase (TYR)-like activity of MOF-818 was determined by measuring the generation of benzoquinone compounds. In brief, MOF-818 (1 mg mL^{-1}) were mixed with APAP (1 mM). Subsequently, the UV visible absorption spectra were recorded at the wavelength of 450 nm. The Michaelis-Menten constant (K_m) and maximum reaction rate (V_{max}) of the multiple enzyme-like activities exhibited by MOF-818 towards various substrates were calculated based on the Michaelis-Menten curves.

5,5'-dithiobis-(2-nitrobenzoic acid, DTNB) was used as a substrate to detect the GSHOx-like activity of MOF-818, which reacts with GSH to form a 5′-thio-2-nitrobenzoic acid (TNB) chromophore with a maximum absorption peak at 412 nm [5]. In brief, different concentrations of MOF-818 NPs were incubated with GSH solution (0.1 mM) at 37°C for 1 h, followed by centrifuged, 0.5 mL of supernatant was mixed with 25 mL of DTNB solution (1 mg mL^{-1}) for 15 min. Finally, the absorption spectrum of the mixture was detected at the wavelength of 412 nm by UV visible spectrophotometer.

For the detection of MOF-818's GOx-like activity, the reaction product, gluconic acid, was assayed. After different concentrations (0, 5, 50, and 500 μg mL^{-1}) of MOF-818 were mixed with 1 mg mL^{-1} glucose solution, pH values were recorded every 15 min using a pH meter (phs-3c, PUCHUN, Shanghai, China). In addition, the detection of MOF-818's GOx-like activity was also achieved by initiating a reaction between gluconic acid and hydroxylamine, followed by complexation with $FeCl_3$, which results in the formation of a red complex with a major absorbance at 505 nm

[6]. In detail, the MOF-818 NPs (1 mg mL^{-1}) were stirred with 5 mL glucose (100 mM) at 37°C to measure pH values using a pH meter. 250 μL of solution I (5 mM EDTA and 0.15 mM triethylamine) and 25 μL of solution II (3 M NH_2OH) were added to the catalytic reaction. After incubation for 25 min, 125 μL of solution III (1 M HCl, 0.1 M $FeCl_3$, and 0.25 M CCl_3COOH) was added to the above solution followed by 5-min reaction. The produced red complex was detected at the wavelength of 505 nm by UV visible spectrophotometer.

To determine the multienzyme-mimicking activities of MOF-818 (5 μg mL^{-1}) and the activation of APAP under varying pH and H_2O_2 concentrations, both PBS systems and double-distilled water (ddH_2O) systems were prepared with gradient settings of pH (6.5 and 7.4) and hydrogen peroxide concentrations (10 nM, 1 μM and 100 μM). PBS systems were designated for the assay of CAT, POD, GSHOx, SOD, and TYR-like activities, and ddH_2O systems for the exclusive assay for GOx-like activity.

Cellular uptake of MOF-818

AgNCs-AS1411@MOF-818 composites were obtained *via* reduction of $AgNO_3$ using $NaBH_4$. In detail, AS1411 (125 μL, 200 μM) with the sequence 5′-GGT GGT GGT GGT TGT GGT GGT GGT GGT TTC CCT AAC TCC CC-3′ was heated at 95°C for 5 min, then quickly cooled at 0°C for 20 min. MOF-818 (5 mg mL^{-1}, 250 μL) was added to the above solution together with ammonium acetate buffer (90 μL, 1 mM, pH 7.0) and $AgNO_3$ (25 μL, 1 mg mL^{-1}). Then, the mixture was cooled with ice. After 15 min, the mixture was reduced with pre-cooled $NaBH_4$ (410 μL, 13.4 μg mL^{-1}) and shaken intensively for 1 min to form AgNCs. Finally, AgNCs-AS1411@MOF-818 composites were obtained after 24 h at 0 °C.

HCC1806 cells were seeded on confocal dishes with a glass-bottom insert

(Biosharp, China) and incubated with AgNCs-AS1411@MOF-818 (5 μg mL^{-1}) for 3, 6, 12, and 24 h. After washed three times with PBS, cells were fixed with 4% paraformaldehyde for 30 min. DiO (5 μM) was then used to visualize the cytomembrane. Furthermore, cellular uptake was confirmed by using a confocal laser scanning microscope (LSM 980, Zeiss, Oberkochen, Germany). ImageJ was employed to quantify the internalization of MOF-818 within the target cells.

Cell viability determination

Cells were cultured in 96-well plates with different treatments. Following a 60-min incubation with pre-cooled 10% trichloroacetic acid at room temperature, the plates were rinsed 5 times with distilled water and subsequent air-dried. The wells were then stained with 50 μL of 0.4% sulforhodamine B (SRB) solution for 5 min on a shaker. After staining, the culture plates were rinsed 5 times with 1% acetic acid and allowed to dry again. Furthermore, to solubilize the SRB dye, 100 μL of 10 mM Tris base solution was added to each well, and the plates were mixed for 5 min. Finally, the absorbance at 515 nm was measured using a plate reader.

The cytotoxic effect of msTRAIL and FT on cells was evaluated using CCK-8 staining kit according to instructions. The absorbance values at 450 nm were measured. Finally, the half maximal inhibitory concentration (IC_{50}) value was calculated using nonlinear regression analysis.

Intracellular GSH measurement

The intracellular GSH levels were measured using a micro reduced glutathione assay kit (Solarbio, Beijing, China). Specifically, HCC1806 cells following treatments were washed twice with PBS. Then, the resuspended cells were subjected to a frozen-thaw

cycle in liquid nitrogen three times. After centrifugation of the lysate, the supernatant was mixed with DTNB. The absorbance at 412 nm was then measured.

Intracellular ROS monitoring

22RV1 and HCC1806 cells were seeded in 24-well plates and exposed to various treatments either for 48 h (for monitoring ROS) or 24 h (for assessing $O_2^{\cdot-}$, H_2O_2, and ·OH). Then, cells were incubated with the fluorescence probes of 2,7-dichlorodihydrofluorescein acetoacetic acid (DCFDA, 10 μM), HKSOX-1r (2 μM), HKPerox-2 (5 μM), or HKOH-1r (5 μM) for 30 min in a dark environment at 37 °C. The fluorescence intensity was detected using a fluorescence microscope. ImageJ software was used to calculate the change in total ROS, $O_2^{\cdot-}$, H_2O_2, and ·OH levels.

Intracellular O_2 monitoring

HCC1806 cells were seeded into 24-well plates and subjected to certain treatments for 24 h. Then, cells were incubated with tris(4,7-diphenyl-1,10-phenanthroline) ruthenium (II) dichloride (5 μM) for 6 h in a dark environment at 37 °C. The fluorescence intensity was detected with a fluorescence microscope. ImageJ was used to calculate the change in O_2 levels.

The determination of apoptotic status of cells

HCC1806 cells were seeded in six-well plates and exposed to different treatments. Subsequently, the cells were trypsinized and washed three times with cold PBS. Furthermore, the cells were incubated with 50 μL mL^{-1} annexin V-FITC and 25 μL mL^{-1} PI in binding buffer for 15 min at room temperature before being analyzed by

flow cytometry.

Fluorescent staining of actin filaments and cellular membranes

We modified the MOF-818 administration method by consuming intracellular glucose using 10 ng μL^{-1} of MOF-818 for 24 h, followed by incubation with APAP for an additional 24 h. Then, the treated cells were fixed with 4% paraformaldehyde. Therefore, cells were incubated in Actin-Tracker Red-555 diluted at a ratio of 1:100 in the dark for 30 min, followed by incubation with the green membrane-staining dye DiO (10 μM) for additional 10 min. The cells were then washed once with PBS, and fluorescence images were captured using a confocal microscope.

The determination of mitochondrial membrane potential

After HCC1806 cells were treated for 48 h, a JC-1 staining solution was used to incubate the cells for 20 min. Subsequently, a fluorescence microscope was employed to capture images of JC-1 monomers using the green channel and of JC-1 aggregates using the red channel. ImageJ was used to calculate the fluorescence intensity, representing a change of mitochondrial membrane potential.

Autophagic flux measurement

Cells were transfected with an AdPlus-mCherry-GFP-LC3B probe. After an 18 h incubation, the cells were grown on a glass slide and treated for another 48 h. Images were acquired with a confocal microscope. The red dots indicated autolysosomes and the yellow dots in the merged images indicated autophagosomes [7]. Autolysosomes and autophagosomes per cell were quantified from at least nine fields of view across three independent experiments. The autolysosome-to-autophagosome ratio was

calculated to represent the autophagic flux.

High performance liquid chromatography-mass spectrometer (HPLC-MS) analysis

HCC1806 cells were treated with MOF-818 and APAP for 48 h. The treated cells were washed twice with PBS, and repeatedly subjected to a frozen-thaw cycle in liquid nitrogen for three times. After evaporation, the samples were dissolved in methanol and analyzed by HPLC-MS (LTQ Orbitrap XL, Thermo Fisher Scientific, MA, USA).

RNA isolation

Total RNA was isolated using RNeasy Plus Mini Kit (Qiagen, Shanghai, China) according to the manufacturer's instructions. The integrity of the RNA was assessed by Agilent 2100 Bioanalyzer (Agilent CA, USA) and agarose gel electrophoresis. The purity and concentration of the RNA were then determined using a Nanodrop micro-spectrophotometer (Thermo Fisher Scientific, MA, USA). The extracted RNA was finally stored at −80 °C.

RNA sequencing

Total RNA was extracted from the treated cells and sequenced on an Illumina Novaseq platform. The RNA-sequencing analysis was conducted using Hisat2 (v2.0.5) [8] with the reference genome GRCh38 and featureCounts program [9]. Differentially expressed genes (DEGs) were identified using the DESeq2 [10] R package applying a filter of FDR < 0.05 and $\log_2|FC| > 1$. Subsequently, pathway analysis was performed using GSEA (v4.3.2) [11].

Western blot analysis

Cells were lysed by using RIPA buffer (Cell Signaling Technology, MA, USA) supplemented with Halt™ Protease and Phosphatase Inhibitor Single-Use Cocktail (Thermo Fisher Scientific, MA, USA). The concentration of total protein was determined using the bicinchoninic acid (BCA) protein assay kit (Thermo Fisher Scientific, MA, USA). The proteins in cell lysate were then separated using SDS-PAGE and transferred onto polyvinylidene fluoride (PVDF) membranes (Millipore, MA, USA). Subsequently, the membranes were incubated with primary antibodies and corresponding secondary antibodies conjugated with fluorescence tags. Near-infrared fluorescence signals were collected and analyzed using the Odyssey® DLx (LICOR, NE, USA) and ImageJ software.

Protein mass spectrometry

After treatment with HCC1806 cells, the proteins from cells were extracted and separated by SDS-PAGE. The gel was stained with Coomassie brilliant blue. The objected band was cut and performed in-gel tryptic digestion. Peptides were separated at EASY-nLC 1000 UPLC system (Thermo Fisher Scientific, MA, USA). The separated peptides were analyzed in Q Exactive Plus with a nano-electrospray ion source. Precursors and fragments were analyzed at the Orbitrap detector. Finally, the data were processed using PD search engine (v.2.4). Tandem mass spectra were searched against Homo_sapiens_9606_SP_20231220.fasta (20429 entries) database.

***In vivo* anti-tumor efficiency evaluation**

The animal experiments in this study were approved by the Institutional Animal Care and Use Committee, Yunnan University (approval No. YNU20230523). Female

BALB/c nude mice (5 weeks old) were purchased from Beijing Vital River Laboratory Animal Technology Co., Ltd. and placed in a controlled environment at 21–23°C with a 12-h light/dark cycle and 40–70% humidity. GFP-labeled HCC1806 (GFP-HCC1806) cells (1×10^6 cells) in Matrigel were subcutaneously injected into the right armpit of the nude mice. When the tumor volume reached approximately 100 mm^3, the mice were randomly divided into seven groups (n = 5). Different combinations of MOF-818 (25 mg kg^{-1}), APAP (200 mg kg^{-1}), FT (5 mg kg^{-1}), MCT (25 mg kg^{-1} MOF-818 and FT 5 mg kg^{-1}), and 3MCT NPs (2 mg kg^{-1} 3-MA, 25 mg kg^{-1} MOF-818, and 5 mg kg^{-1} FT) were performed tail vein injection every other day for 14 days. Body weights and tumor volumes were recorded every two days, and the tumor volumes were calculated as volume (mm^3) = length × $width^2/2$. At the end of treatment, the mice were sacrificed, and tumor tissue in all treatment groups was collected for histological analysis. The main organs (heart, liver, lung, spleen, and kidney) of the mice were also harvested for hematoxylin and eosin (H&E) staining. Briefly, tissue samples were fixed in 4% paraformaldehyde, embedded in paraffin, sectioned at a thickness of 4 μm, and stained with H&E to visualize nuclear and cytoplasmic/eosinophilic components. Blood was collected for liver and kidney function and blood routine testing. Briefly, blood routine parameters were assessed using a BC-5000vet hematology analyzer (Mindray, Shenzhen, China). Liver and kidney function indices were measured using a Chemray 420 fully automated biochemical analyzer (Rayto, Shenzhen, China).

In vivo biosafety and metabolism evaluation

The levels of Zr and Cu in blood, urine, feces, and organs were measured using ICP-MS to reflect *in vivo* distribution of MOF-818 NPs. For the collection of blood,

urine and feces samples, BALB/c mice (5 weeks old, $n = 3$) from Beijing Vital River Laboratory Animal Technology Co., Ltd. were subcutaneously injected with MOF-818 (50 mg kg^{-1}). To obtain samples from heart, liver, spleen, lung, and kidney, BALB/c mice (5 weeks old, $n = 4$) were administered daily subcutaneous injected for a period of 30 d with MOF-818 (50 mg kg^{-1}).

The concentration of FT in the blood was detected using a Human TRAIL/TNFSF10 Quantikine ELISA Kit according to the instructions. The biosafety of 3MCT was evaluated after *in vivo* anti-tumor treatments (See the Methods section for details). Using GFP-labeled HCC1806-bearing mice with different treatments, blood routine and the function indicators of liver and kidney were examined. The contents of Cu and Zr in organs from GFP-labeled HCC1806-bearing mice under different treatments were determined by ICP-MS.

Statistical Analysis

All experiments were repeated at least three times. Quantitative data were expressed as mean ± SD. Statistical comparisons were conducted using Student's two-tailed *t* test. *p* values less than 0.05 were considered statistically significant: $*p < 0.05$, $**p < 0.01$, and $***p < 0.001$.

Supplemental figures

Table S1. Kinetic parameters of MOF-818 and other enzymes for peroxidase-like activity.

Nanozyme	Substrates	K_m (mM)	V_{max} (mM min^{-1})	Ref.
MOF-818	H_2O_2	58.6	4.0×10^{-2}	[12]
	TMB	0.404	4.71×10^{-2}	
Pd-Fe_3O_4_middle	H_2O_2	126.73	4.0×10^{-2}	[13]
	TMB	0.017	1.51×10^{-2}	
Pt hollow	H_2O_2	6.9	5.94×10^{-3}	[14]
	TMB	0.81	7.2×10^{-3}	
Fe-MIL-88B-NH_2	H_2O_2	1.3	1.5×10^{-3}	[15]
	TMB	2.6	3.36×10^{-3}	
Horseradish peroxidase	H_2O_2	3.70	5.23×10^{-3}	[16]
	TMB	0.434	6×10^{-3}	
MOF-818	H_2O_2	0.3553	4.4×10^{-3}	This work
	TMB	1.1240	6.2×10^{-3}	

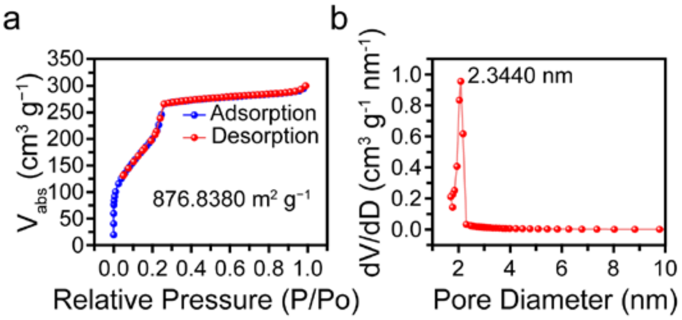

Figure S1. N_2 adsorption-desorption isotherm of our synthesized MOF-818 (a) and its pore distribution (b).

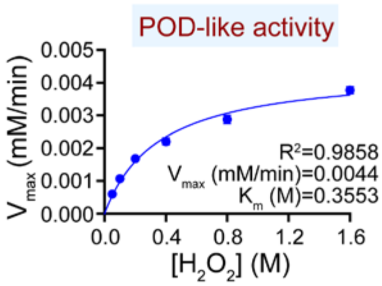

Figure S2. Michaelis-Menten curves of MOF-818 as a POD mimic in the presence of different H_2O_2 concentrations and 0.3 mM TMB.

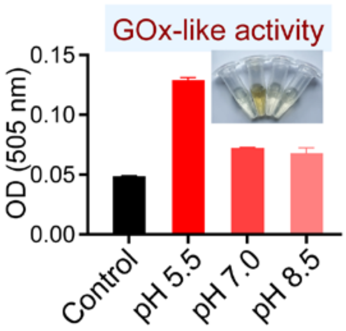

Figure S3. Glucose oxidase (GOx)-like activity of MOF-818 under various pH conditions. Data are given as mean ± SD (n = 3).

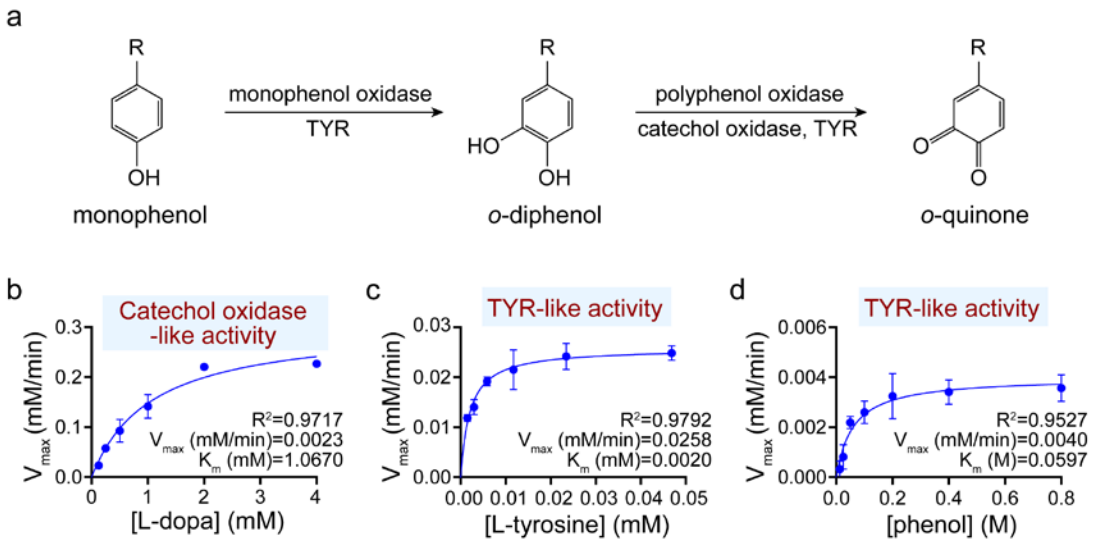

Figure S4. (a) Schematic illustration of tyrosinase (TYR) activity, including monophenol and polyphenol oxidase activities. (b) Michaelis-Menten curve of MOF-818 as a catechol oxidase mimic using *o*-diphenol (L-dopa) as a substrate. Michaelis-Menten curves of MOF-818 as a TYR mimic using L-tyrosine (c), and phenol (d) as substrates. Data are given as mean ± SD (n = 3).

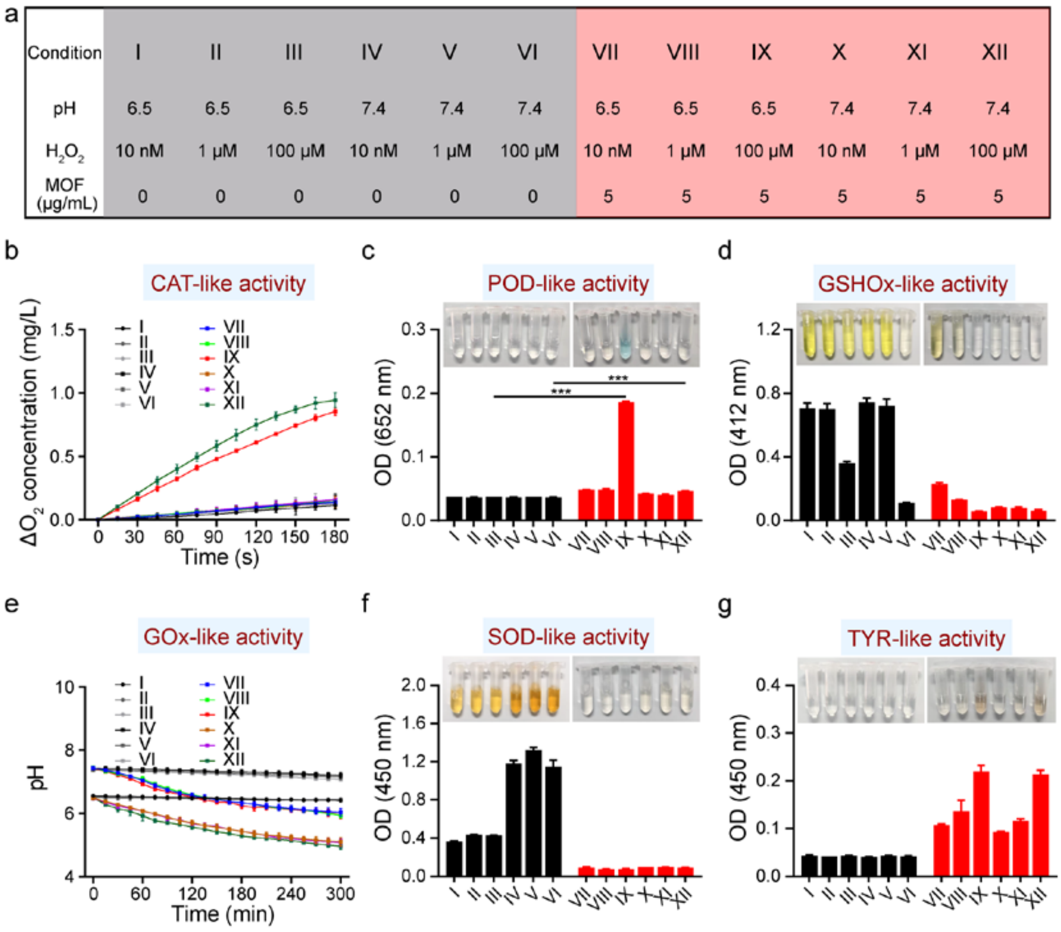

Figure S5. Multi-enzyme-mimicking activities of MOF-818 and its activation of APAP under varying pH (6.5 and 7.4) and H_2O_2 concentrations (10 nM, 1 μM, and 100 μM). (a) Schematic of the twelve experimental groups: conditions I to VI (without MOF-818) and VII-XII (with MOF-818), covering the indicated pH and H_2O_2 gradients. Evaluation of CAT-like (b), POD-like (c), GSHOx-like (d), GOx-like (e), SOD-like activities (f) under the corresponding conditions. (g) TYR-like activity in the absence of presence MOF-818 at a substrate concentration of 1 mM APAP. Data are given as mean ± SD (n = 3). ***$p < 0.001$.

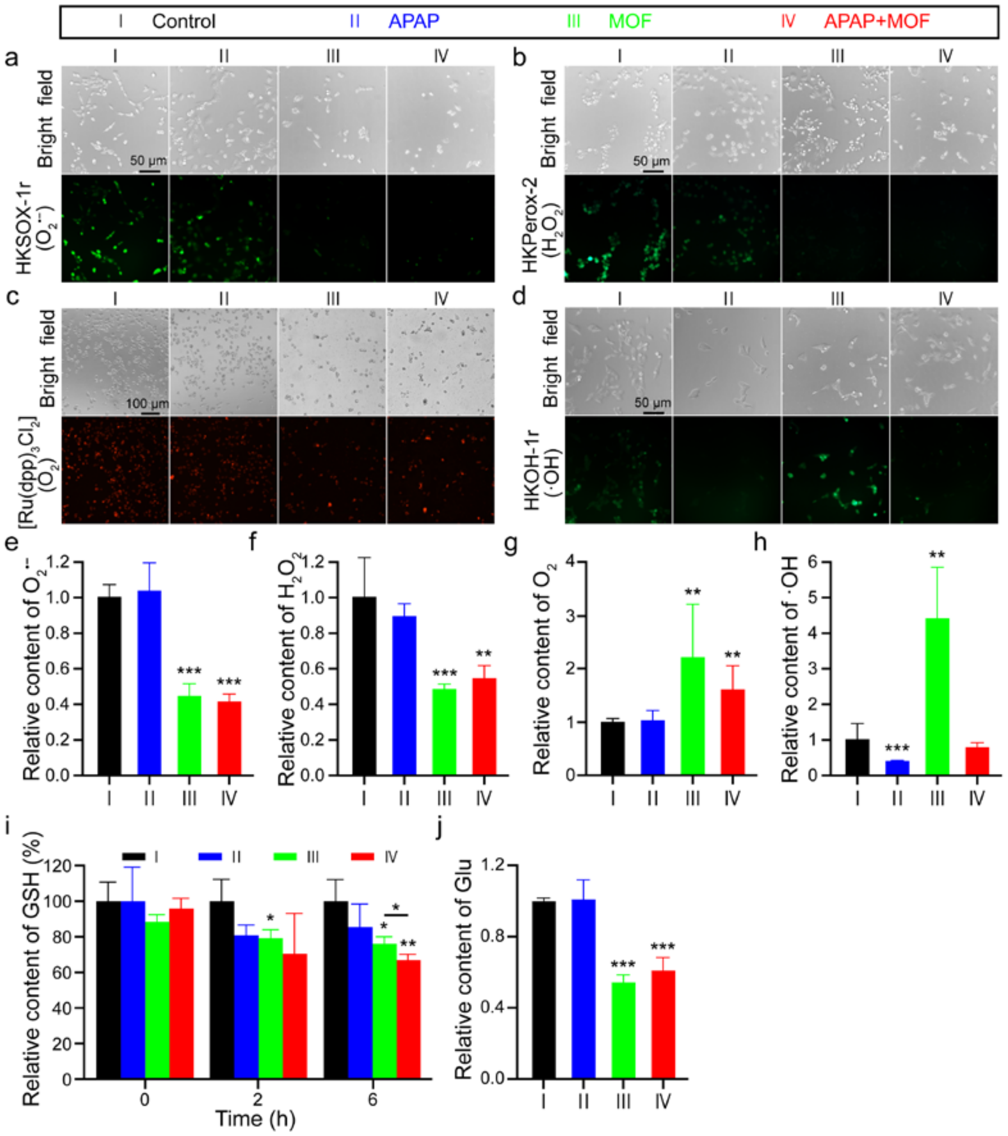

Figure S6. Tumor microenvironment (TME) remodeling induced by MOF-818 coupled with APAP. The bright field images of HCC1806 cells under different treatments for 24 h and the fluorescence images using $O_2^{\cdot-}$ probe (a), H_2O_2 probe (b), O_2 probe (c), and ·OH probe (d). Relative contents of $O_2^{\cdot-}$ (e), H_2O_2 (f), O_2 (g), and ·OH (h) in HCC1806 cells after different treatments for 24 h. (i) Intracellular GSH changes over time after different treatments in HCC1806 cells. (j) Relative content of glusocse (Glu) in HCC1806 cells after different treatments for 48 h. Groups I to IV are control, APAP, MOF-818, and APAP+MOF-818 treatments, respectively. Data are given as mean ± SD (e–h: n = 9; i,j: n = 3). $*p < 0.05$, $**p < 0.01$, $***p < 0.001$.

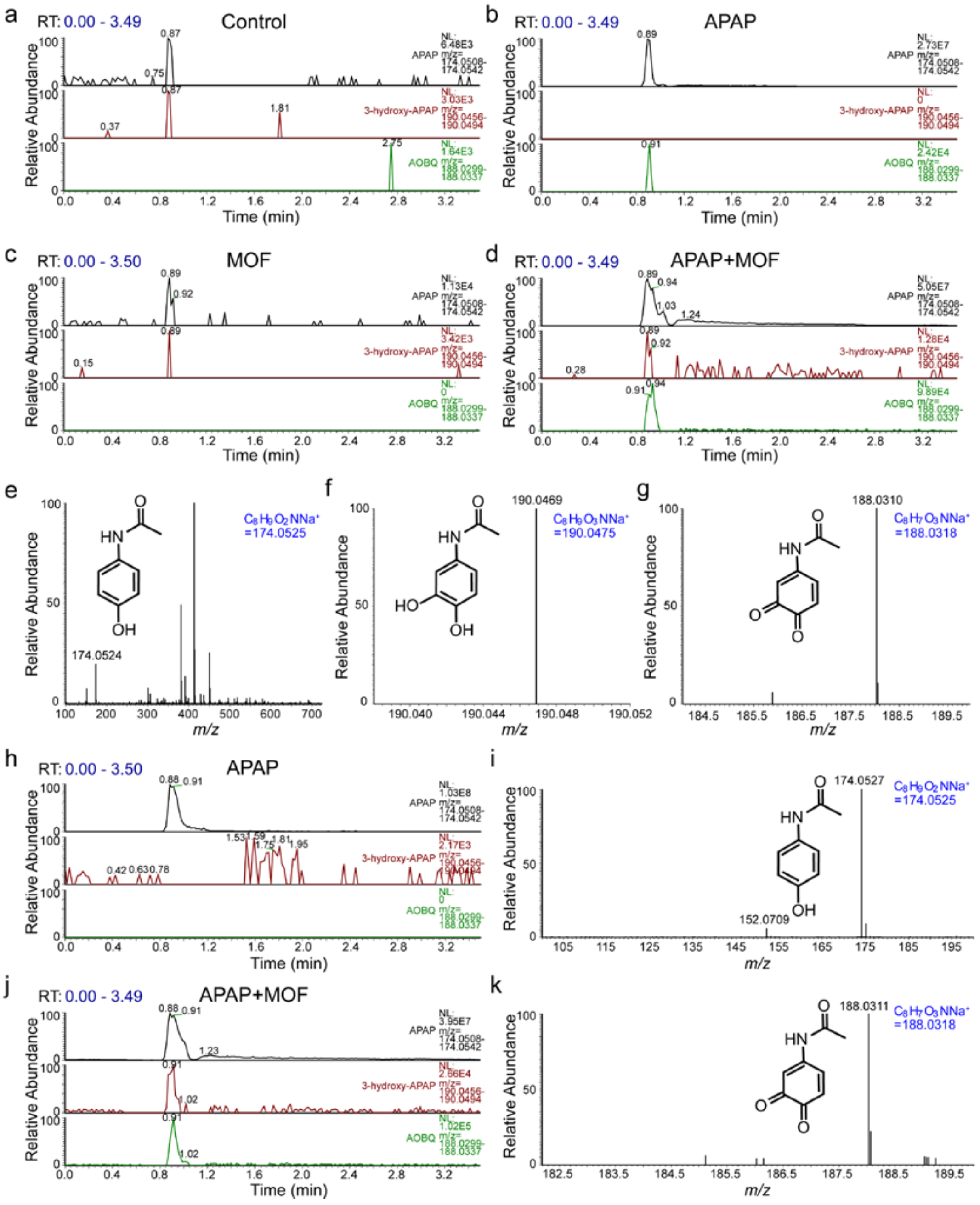

Figure S7. Tyrosinase (TYR)-mimicking activity of MOF-818 by high performance liquid chromatography-mass spectrometer (HPLC-MS) analysis. The HPLC results from HCC1806 cells without treatment (a), treated with acetaminophen (APAP) (b), MOF-818 (c), and APAP and MOF-818 (d). The MS results of APAP abundant peak (e), 3-hydroxy-APAP abundant peak (f), 4-acetamido-*o*-ben-zoquinone (AOBQ) abundant peak (g) from (d). HPLC results from APAP treatment (h) and the combination treatment of APAP and MOF-818 (J) in PBS solution in a tube. The MS results of APAP abundant peak (i) and AOBQ abundant peak (k) from (h) and (j), respectively.

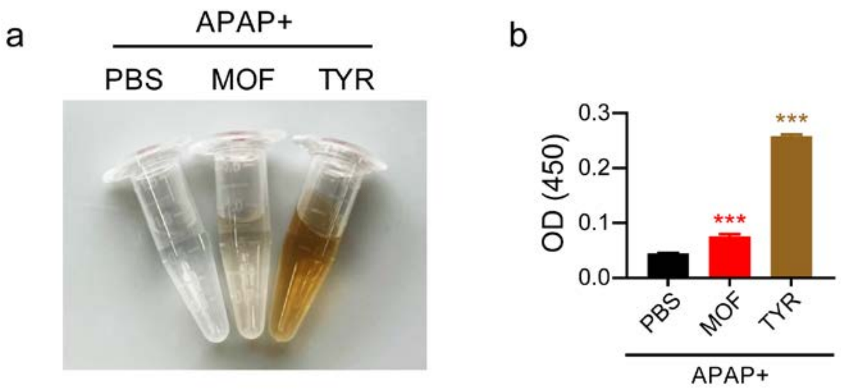

Figure S8. Image of the products of PBS, MOF-818 (1 mg mL^{-1}) or TYR (20 μg mL^{-1}) co-incubated with 1 mM APAP (a) and absorbance values of the products at 450 nm (b). Data are given as mean ± SD (n = 3). ***$p < 0.001$.

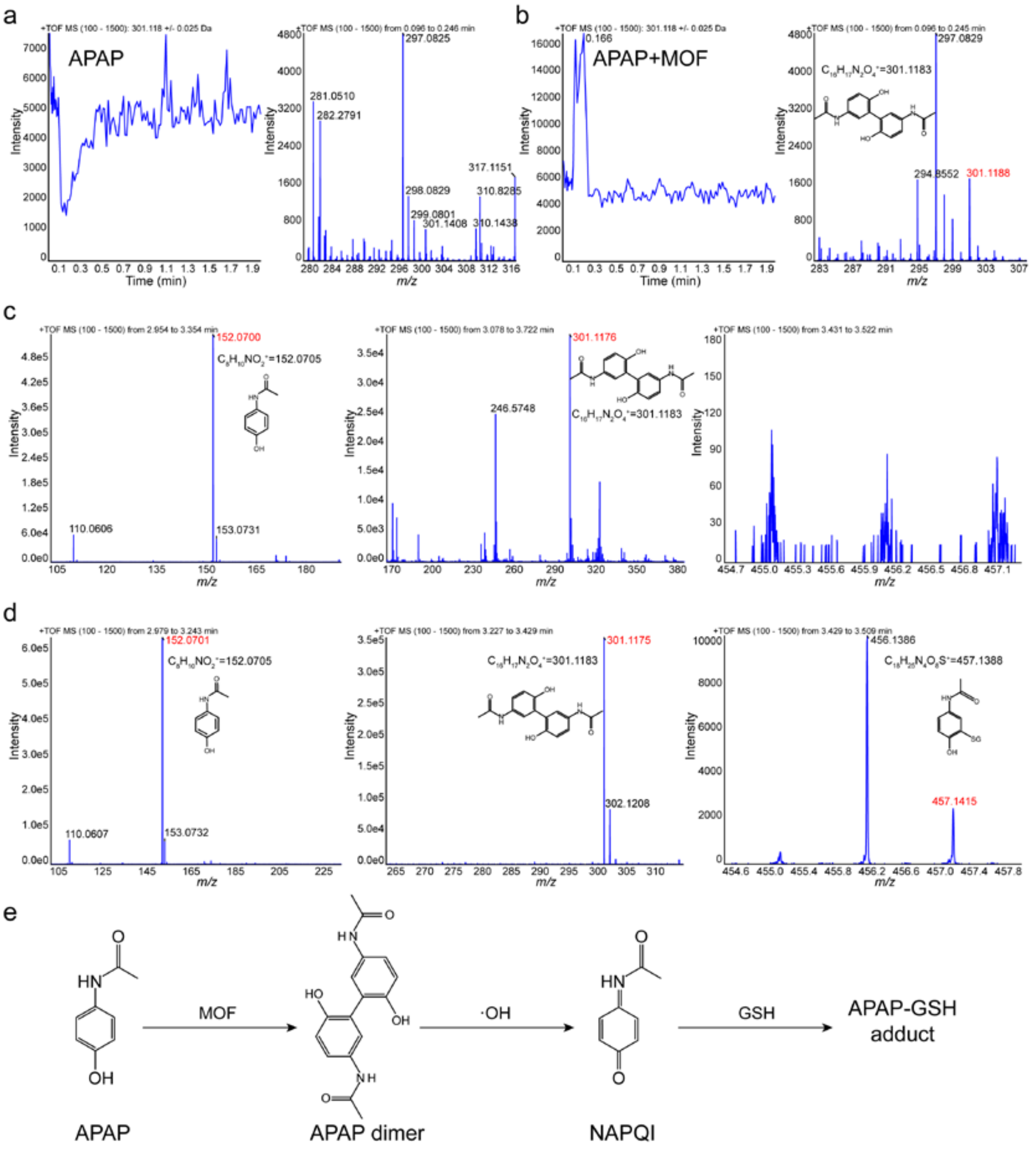

Figure S9. MOF-818 catalyzes APAP to generate *N*-acetyl-*p*-benzoquinone imine (NAPQI) in the presence of ·OH. HPLC-MS of APAP (a) and APAP and MOF-818 (b) in PBS solution in tubes. The abundant peak at *m/z* 301.12 corresponded to the APAP dimer. The MS results of APAP (c) and APAP and MOF-818 (d) in the presence of H_2O_2 and glutathione (GSH) in a tube. From left to right, abundant peaks of APAP, APAP dimer, and APAP-GSH, respectively. (e) Schematic diagram of catalytic process from APAP to APAP-GSH adduct by MOF-818.

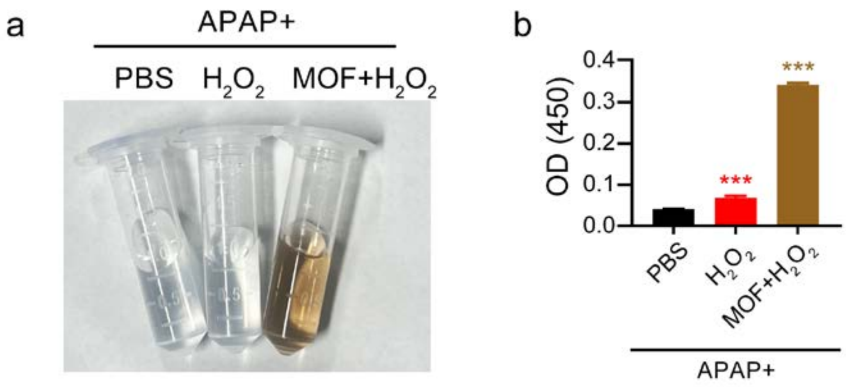

Figure S10. Image of the products of PBS, H_2O_2 or MOF-818+H_2O_2 co-incubated with APAP (a) and absorbance values of the products at 450 nm (b). Data are given as mean ± SD (n = 3). ***$p < 0.001$.

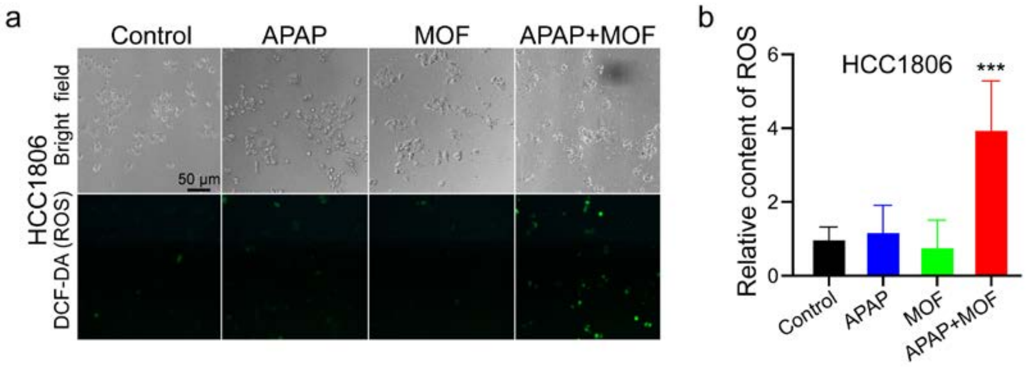

Figure S11. Changes in intracellular reactive oxygen species (ROS) levels under different treatments for 48 h. (a) The bright field images of HCC1806 cells and the fluorescence images of ROS measured by DCF-DA probe. (b) The changes of ROS content in HCC1806 cells. Data are given as mean ± SD (n = 9). ***$p < 0.001$.

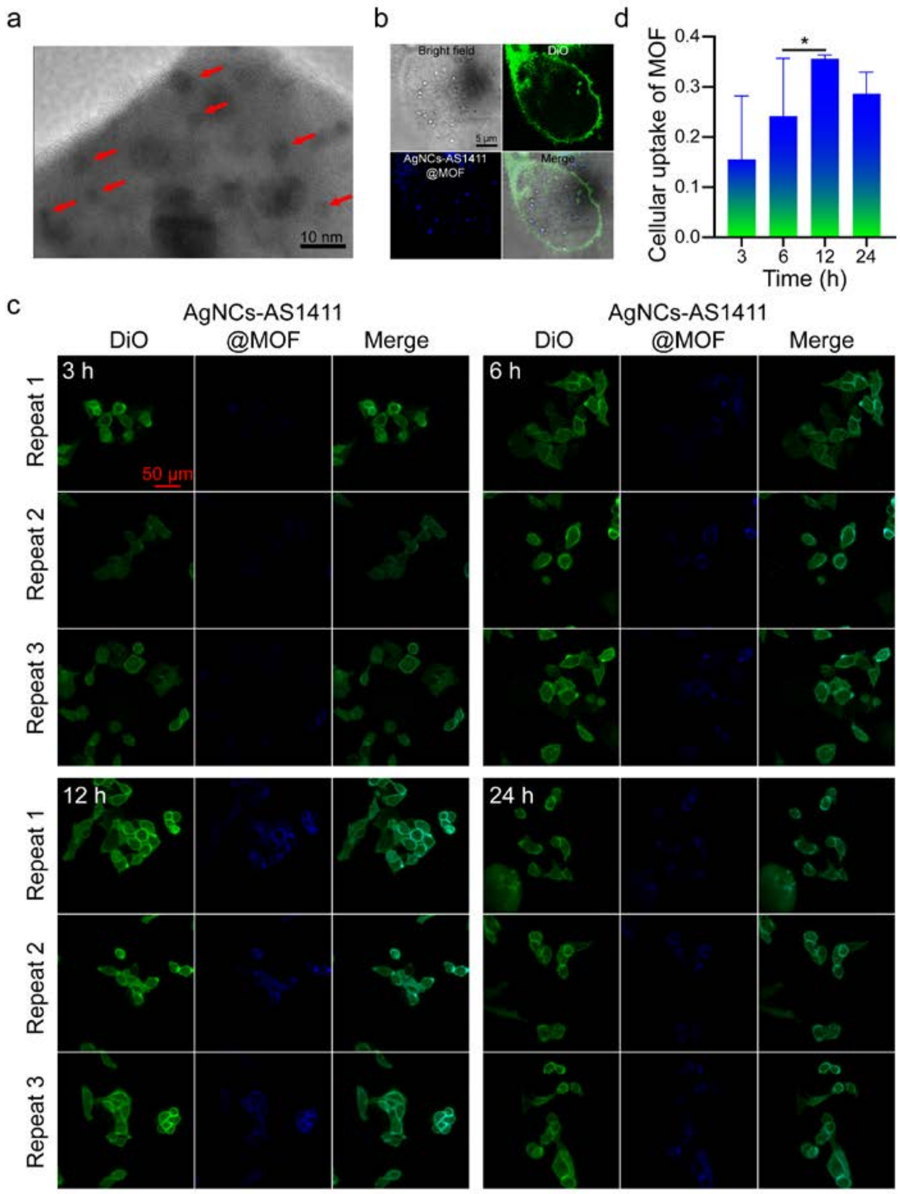

Figure S12. The uptake of AgNCs-AS1411@MOF-818 in HCC1806 cells. **(**a) Transmission electron microscope (TEM) image of AgNCs-AS1411@MOF-818. Red marked arrows indicate the silver nanoclusters of AgNCs-AS1411@MOF-818. Confocal microscopy images of AgNCs-AS1411@MOF-818 uptake by HCC1806 cells for a 12-h incubation (b) and in different incubation times (c). (d)The quantitative analysis of (c). Data are given as mean ± SD (n = 3). *$p < 0.05$.

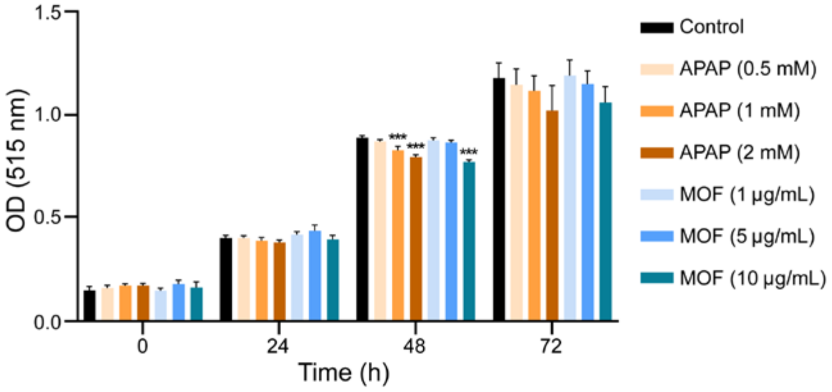

Figure S13. The proliferation of HCC1806 cells in the presence of different concentrations of APAP or MOF-818 alone measured by sulforhodamine B (SRB) colorimetric assay. Data are given as mean ± SD (n = 3). $*p < 0.05$, $**p < 0.01$, $***p < 0.001$.

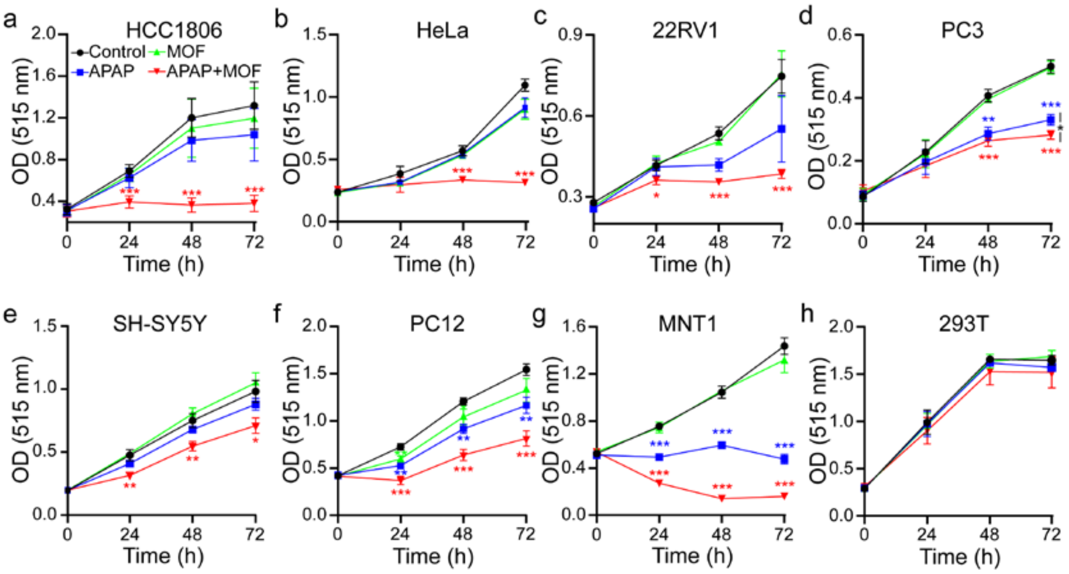

Figure S14. The proliferation assays of HCC1806 (a), HeLa (b), 22RV1 (c), PC3 (d), SH-SY5Y (e), PC12 (f), MNT1 (g), and 293T cells (h) with different treatments measured by SRB colorimetric assay. Data are given as mean ± SD (n = 3). *$p < 0.05$, **$p < 0.01$, ***$p < 0.001$.

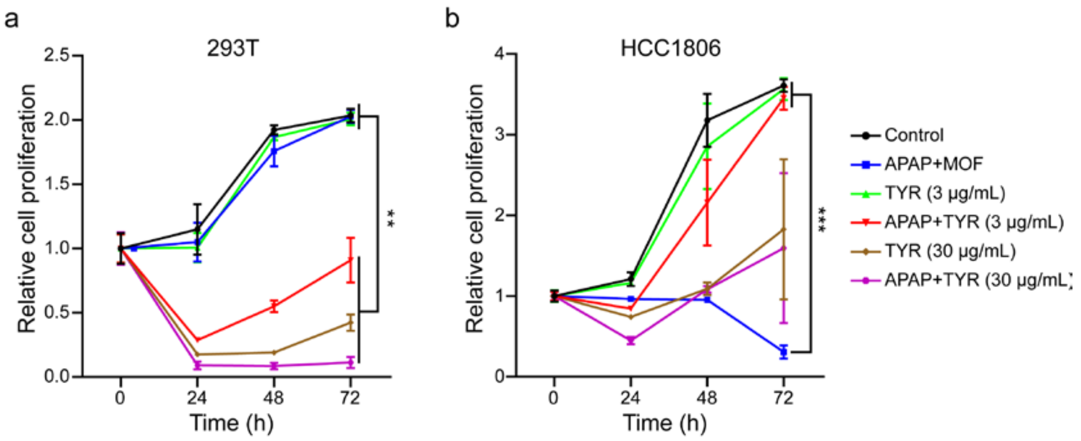

Figure S15. The proliferation assays in 293T (a) and HCC1806 (b) cells with different treatments by SRB colorimetric assay. Data are given as mean ± SD (n = 3). $^{**}p < 0.01$, $^{***}p < 0.001$.

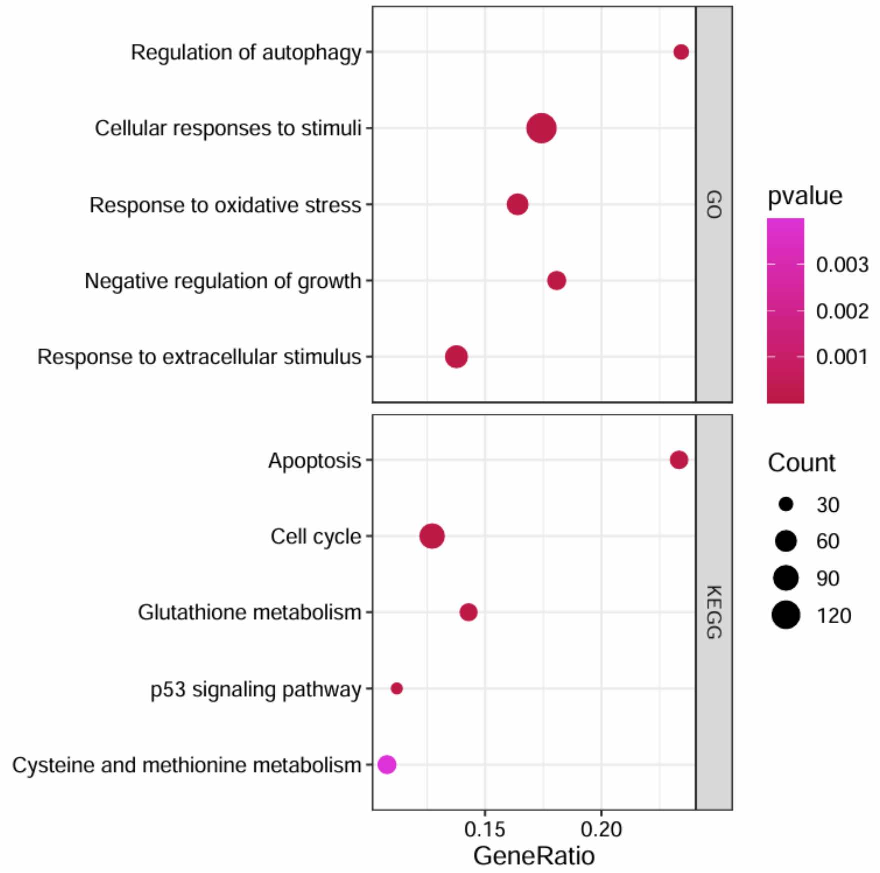

Figure S16. GO and KEGG enrichment analysis of differentially expressed genes between untreated control (*n* = 3) and APAP+MOF-818-treated HCC1806 cells (*n* = 3).

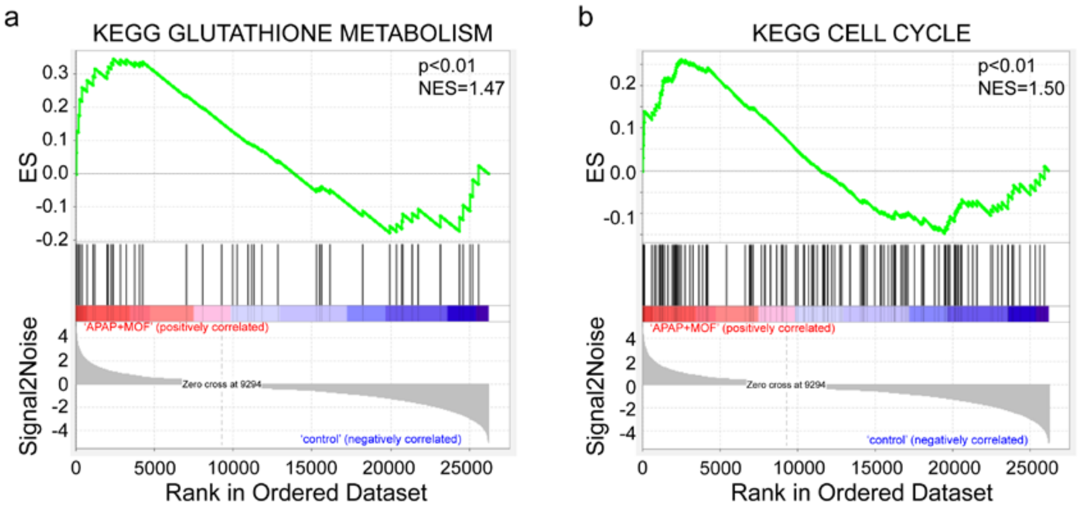

Figure S17. Gene set enrichment analysis (GSEA) in glutathione metabolism (a) and cell cycle (b) between untreated control (n = 3) and APAP+MOF-818-treated HCC1806 cells (n = 3).

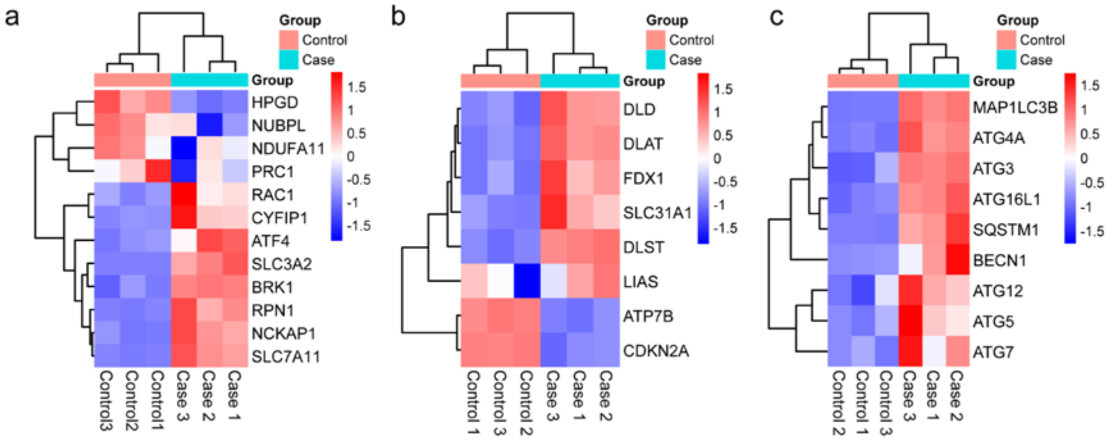

Figure S18. Heatmap showing significant regulations of disulfidptosis (a), cuproptosis (b), and autophagy-related key genes (c) between untreated control (n = 3) and APAP+MOF-818-treated HCC1806 cells (n = 3).

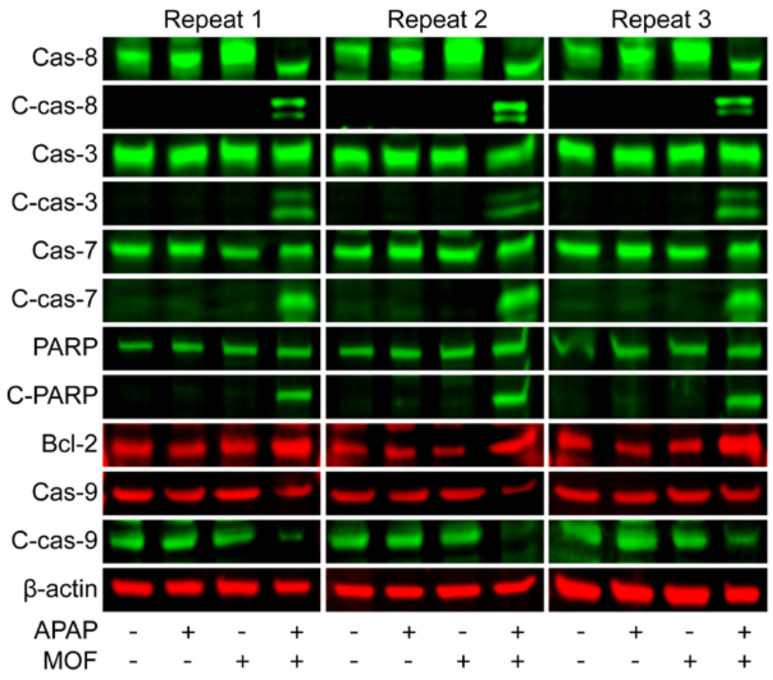

Figure S19. Different repeats of Western blot analysis of the apoptosis-associated proteins in HCC1806 cells following 48 h-treatment with MOF-818 and APAP. Cas-3/7/8/9, caspase 3/7/8/9; C-cas-3/7/8/9, cleaved caspase 3/7/8/9; C-PARP, cleaved PARP.

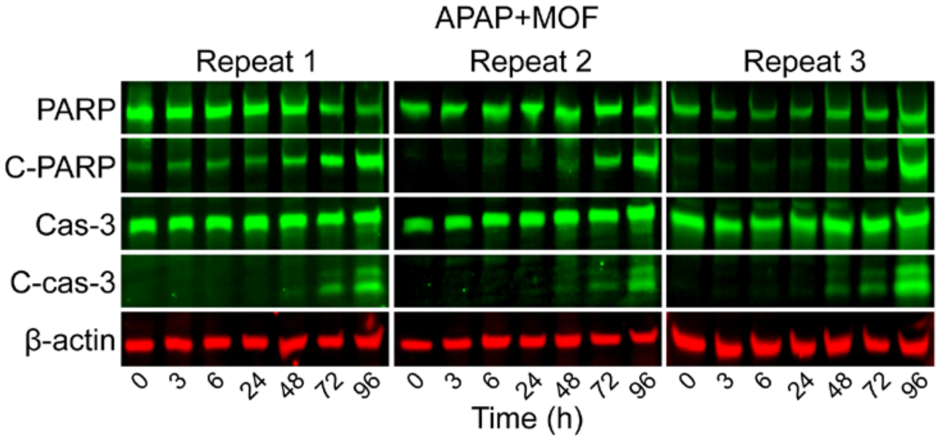

Figure S20. Different repeats of Western blot analysis of the apoptosis-associated proteins in HCC1806 cells with MOF-818+APAP treatment for different times. Cas-3, caspase 3; C-cas-3, cleaved caspase 3; C-PARP, cleaved PARP.

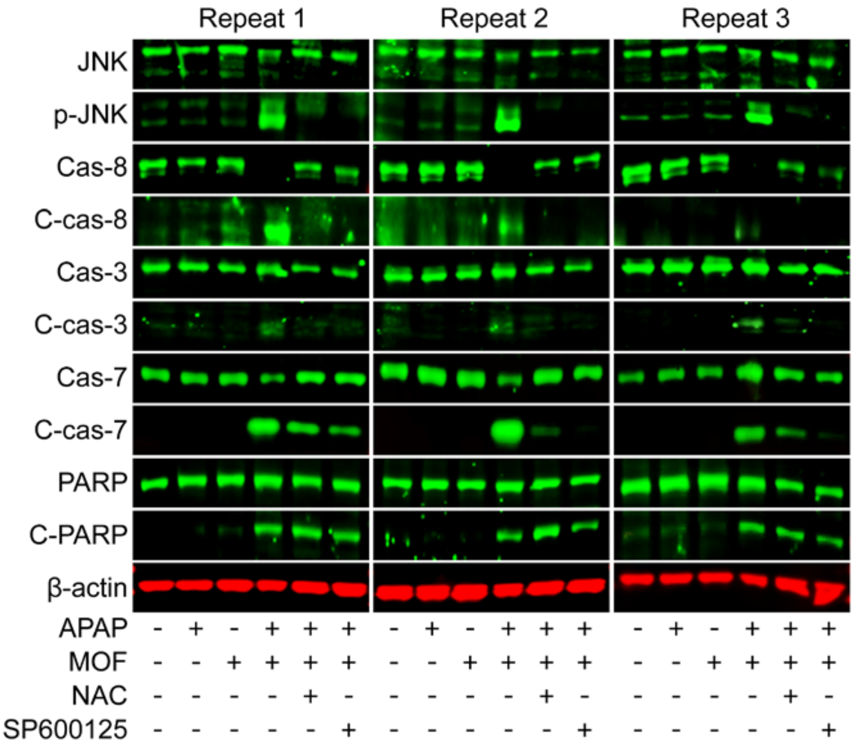

Figure S21. Different repeats of Western blot analysis of several proteins in HCC1806 cells after 96 h-treatments. *N*-acetylcysteine (NAC) is a ROS scavenger and SP600125 is a JNK inhibitor. Cas-3/7/8, caspase 3/7/8; C-cas-3/7/8, cleaved caspase 3/7/8; C-PARP, cleaved PARP.

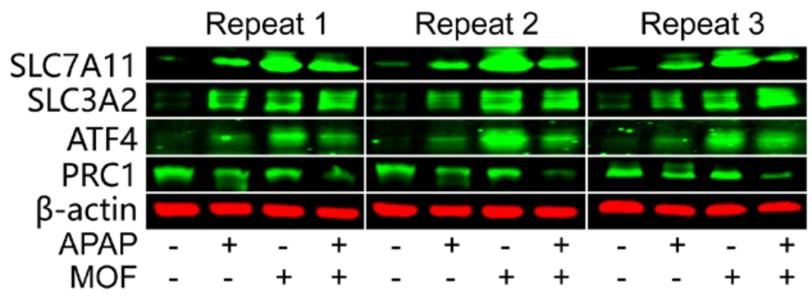

Figure S22. Different repeats of Western blot analysis of SLC7A11, SLC3A2, ATF4, and PRC1 in HCC1806 cells under different treatments.

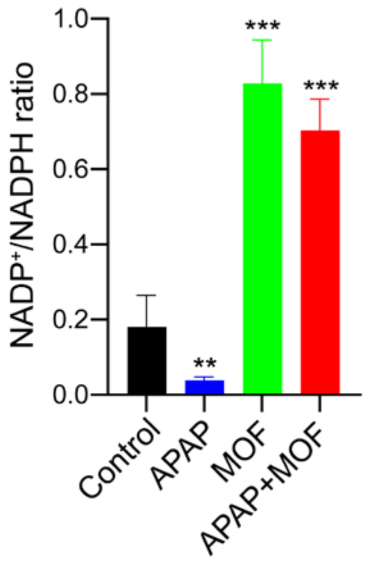

Figure S23. The $NADP^+$/NADPH ratio in HCC1806 cells with different treatments. Data are given as mean ± SD ($n = 3$). **$p < 0.01$, ***$p < 0.001$.

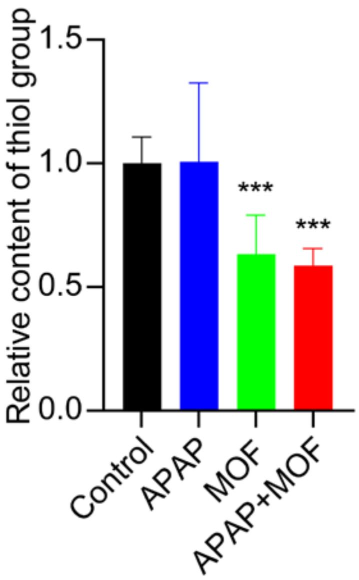

Figure S24. The relative contents of thiol group in HCC1806 cells with different treatments. Data are given as mean ± SD (n = 3). ***$p < 0.001$.

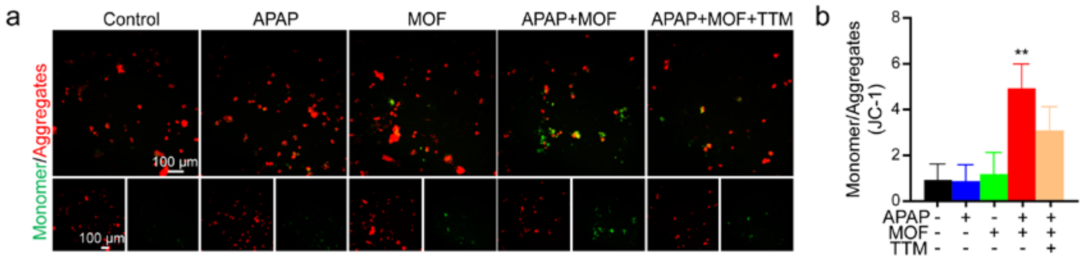

Figure S25. (a) The mitochondrial membrane potentials of HCC1806 cells with different treatments measured by JC-1 staining. (b) The quantitative analysis of (a). Data are given as mean ± SD (n = 3). **$p < 0.01$.

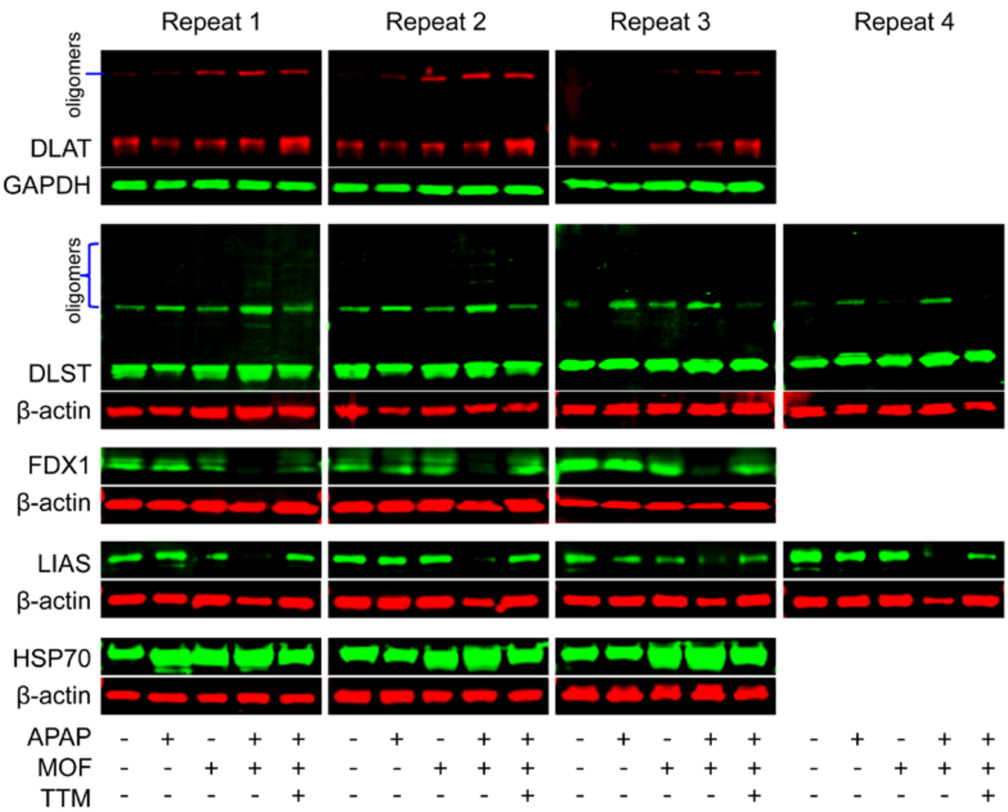

Figure S26. Different repeats of Western blot analysis of the cuproptosis-associated proteins in HCC1806 cells under different treatments. TTM, ammonium tetrathiomolybdate.

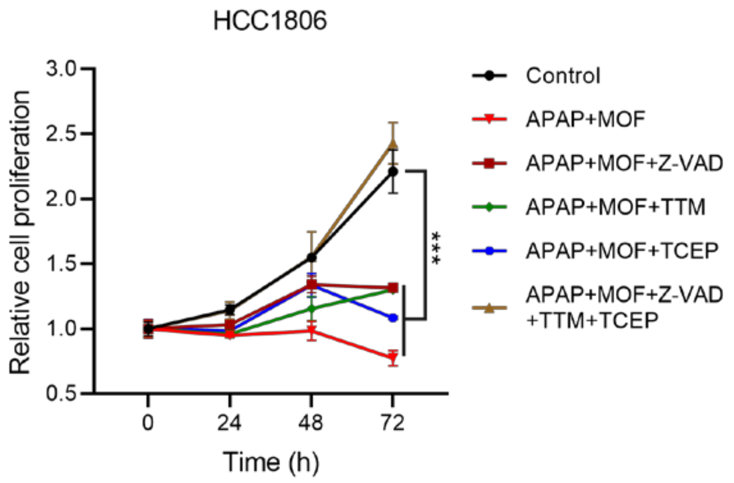

Figure S27. The proliferation of HCC1806 cells with different treatments measured by SRB colorimetric assay. Z-VAD, Z-VAD-FMK, apoptosis inhibitor. TCEP, Tris(2-carboxyethyl)phosphine hydrochloride, disulfidptosis inhibitor. TTM, ammonium tetrathiomolybdate, cuproptosis inhibitor. Data are given as mean ± SD (*n* = 3). ***$p < 0.001$.

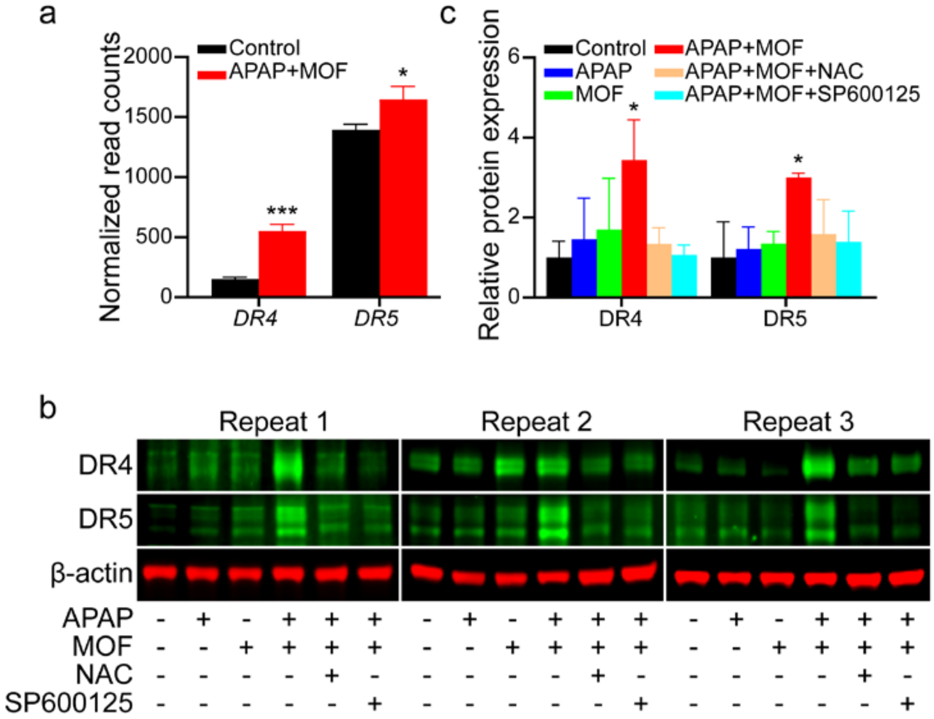

Figure S28. The up-regulation of death receptor 4 (DR4) and death receptor 5 (DR5) induced by the co-administration of MOF-818 and APAP. **(**a) Normalized read counts of *DR4* and *DR5* genes from RNA-sequencing data. **(**b,c) Western blot analysis of DR4 and DR5 in HCC1806 cells under different treatments. *N*-acetylcysteine (NAC) is a ROS scavenger and SP600125 is a JNK inhibitor. Data are given as mean ± SD (n = 3). *$p < 0.05$, ***$p < 0.001$.

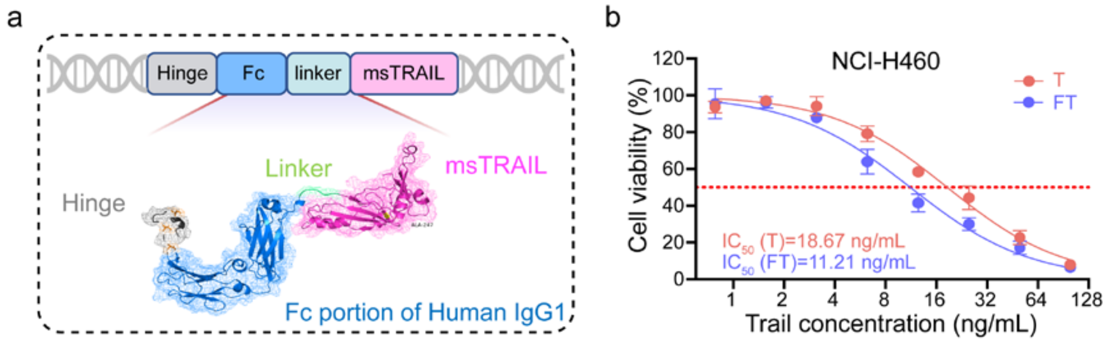

Figure S29. (a) Schematic diagram of the fusion protein Fc-TRAIL (FT) structure. (b) IC_{50} of msTRAIL (T) and FT in human large cell lung carcinoma cells, NCI-H460. Data are given as mean ± SD (n = 3).

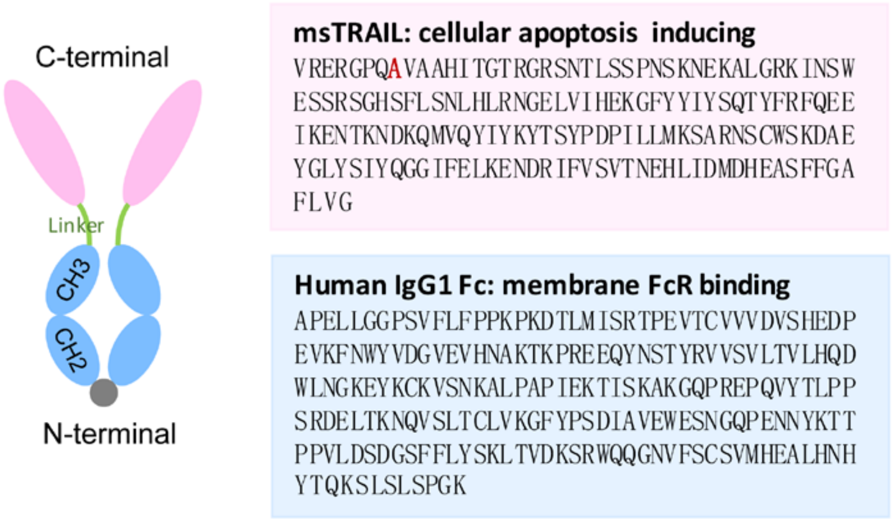

Figure S30. The structural diagram and amino acid sequence information of the fusion protein Fc-TRAIL.

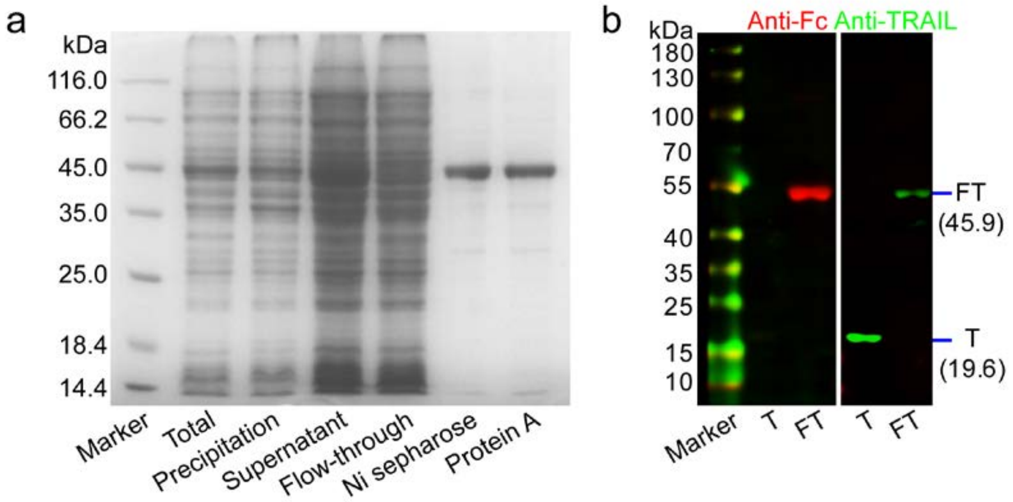

Figure S31. Preparation and characterization of msTRAIL (T) and Fc-TRAIL (FT). (a) Coomassie brilliant blue staining for protein from different purification processes. (b) Western blot analysis of T and FT proteins.

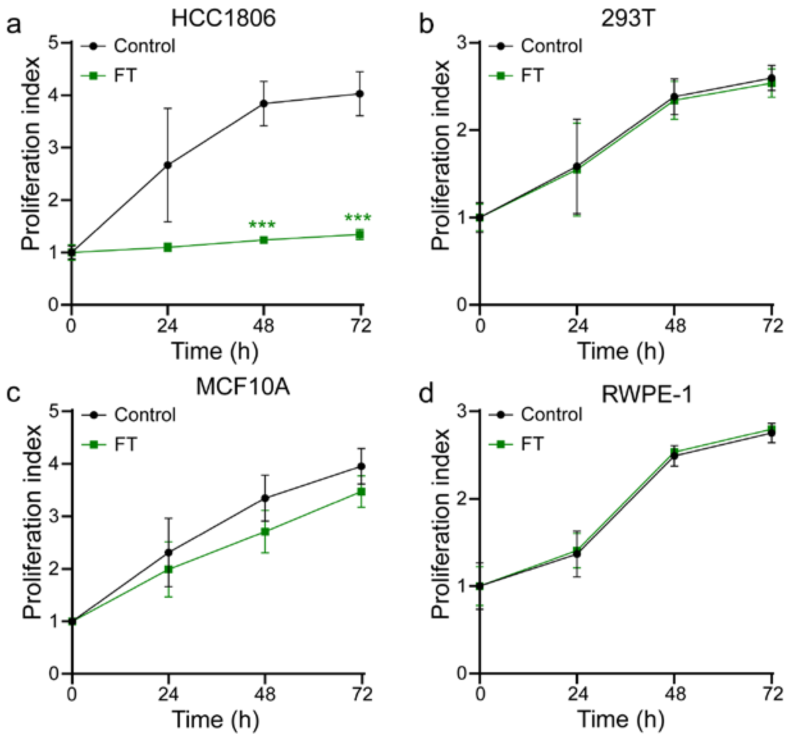

Figure S32. The effect of Fc-TRAIL (FT) on cell proliferation in HCC1806 (a), 293T (b), MCF10A (c), and RWPE-1 cells (d) measured by SRB colorimetric assay. Data are given as mean ± SD (n = 3). ***$p < 0.001$.

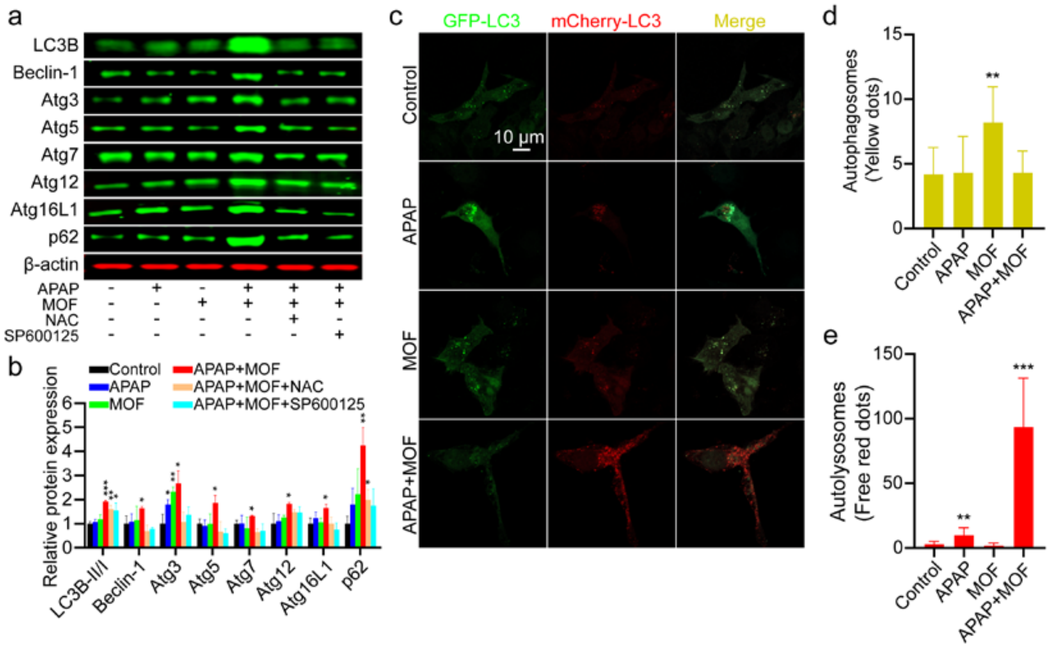

Figure S33. (a,b) Western blot analysis of the autophagy-associated proteins in HCC1806 cells after different treatments. (c) Autophagy flow detected by transfecting mCherry-EGFP-LC3B viruses into HCC1806 cells with different treatments. The colocalization of GFP and mCherry dots was examined and quantified. The autophagosomes (d) and autolysosomes (e) were determined by yellow dots and free red dots from (c), respectively. Data are given as mean ± SD (b: n = 3; d, e: n = 9). $*p < 0.05$, $**p < 0.01$, $***p < 0.001$.

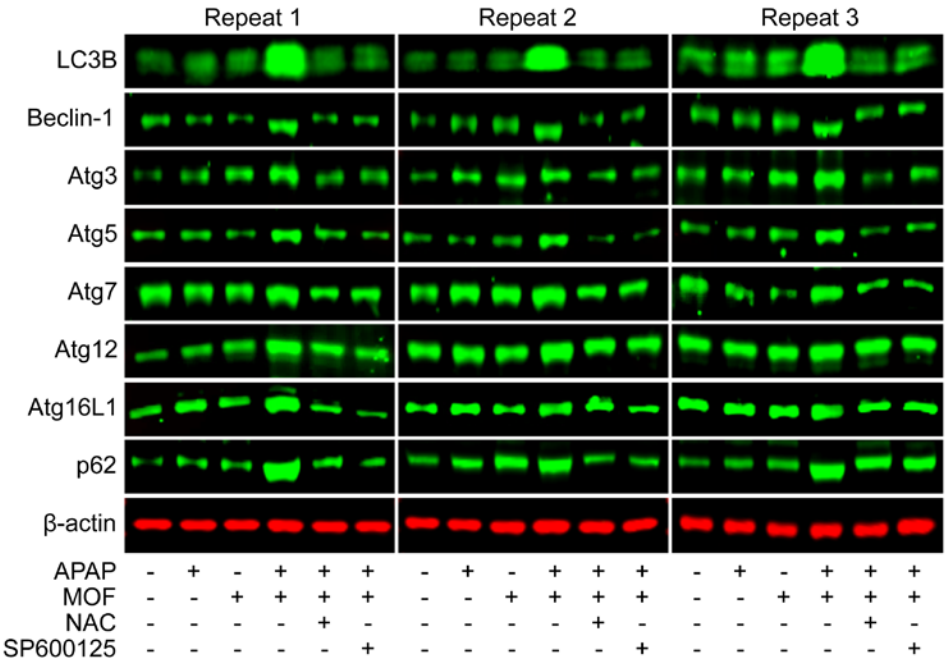

Figure S34. Different repeats of Western blot analysis of the autophagy-associated proteins in HCC1806 cells under different treatments. *N*-acetylcysteine (NAC) is a ROS scavenger and SP600125 is a JNK inhibitor.

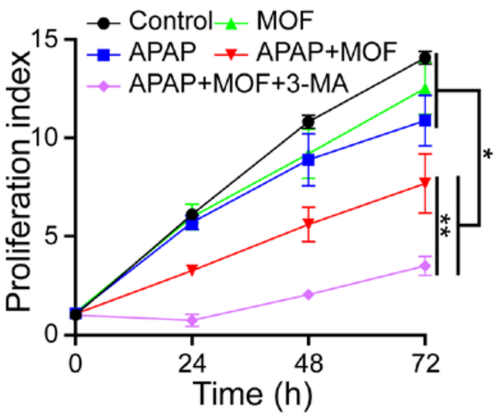

Figure S35. The proliferation of HCC1806 cells with different treatments measured by SRB colorimetric assay. 3-MA, 3-methyladenine. Data are given as mean ± SD (n = 3). *$p < 0.05$, **$p < 0.01$.

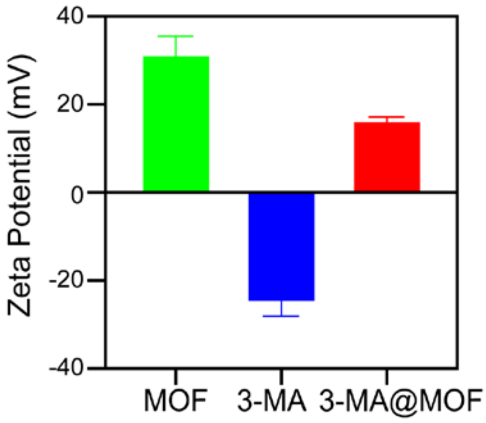

Figure S36. Zeta potentials of MOF-818, 3-MA, and 3-MA@MOF-818. 3-MA, 3-methyladenine. Data are given as mean ± SD (n = 3).

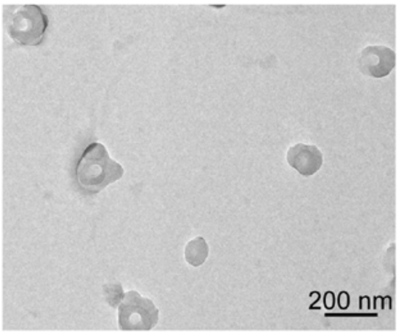

Figure S37. The TEM image of the extracted THP-1 cell membranes.

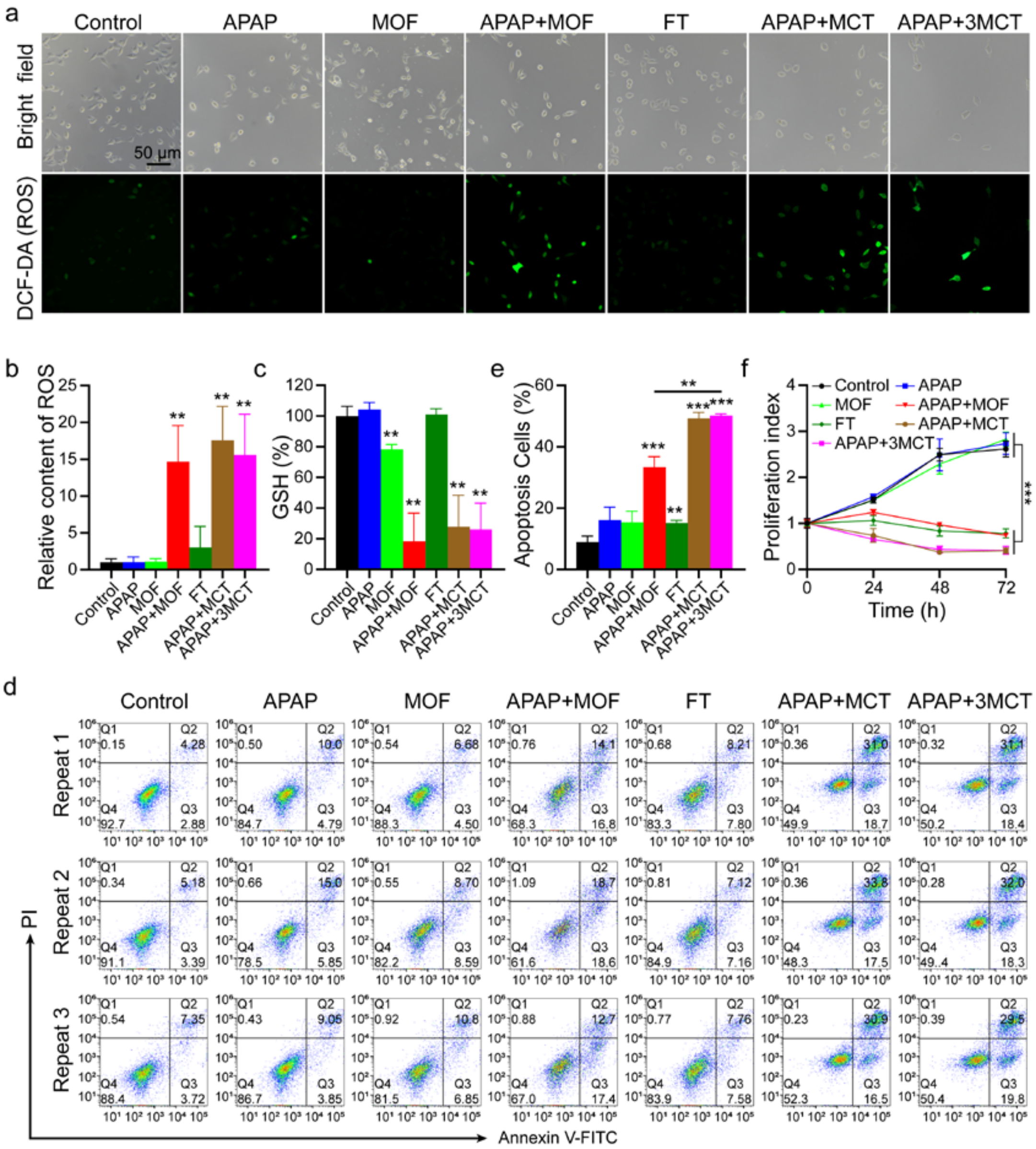

Figure S38. Evaluation of the anti-cancer ability of 3-MA@MOF-818@CM-Fc-TRAIL nanoparticles (3MCT NPs) in HCC1806 cells. The changes of ROS content (a,b) and GSH content (c) in HCC1806 cells with different treatments for 72 h. (d,e) Flow cytometry for apoptosis detection with different treatments for 72 h. (f) The proliferation of HCC1806 cells with different treatments measured by SRB colorimetric assay. Data are given as mean ± SD (b,c: *n* = 9; e,f: *n* = 3). $^{**}p < 0.01$, $^{***}p < 0.001$.

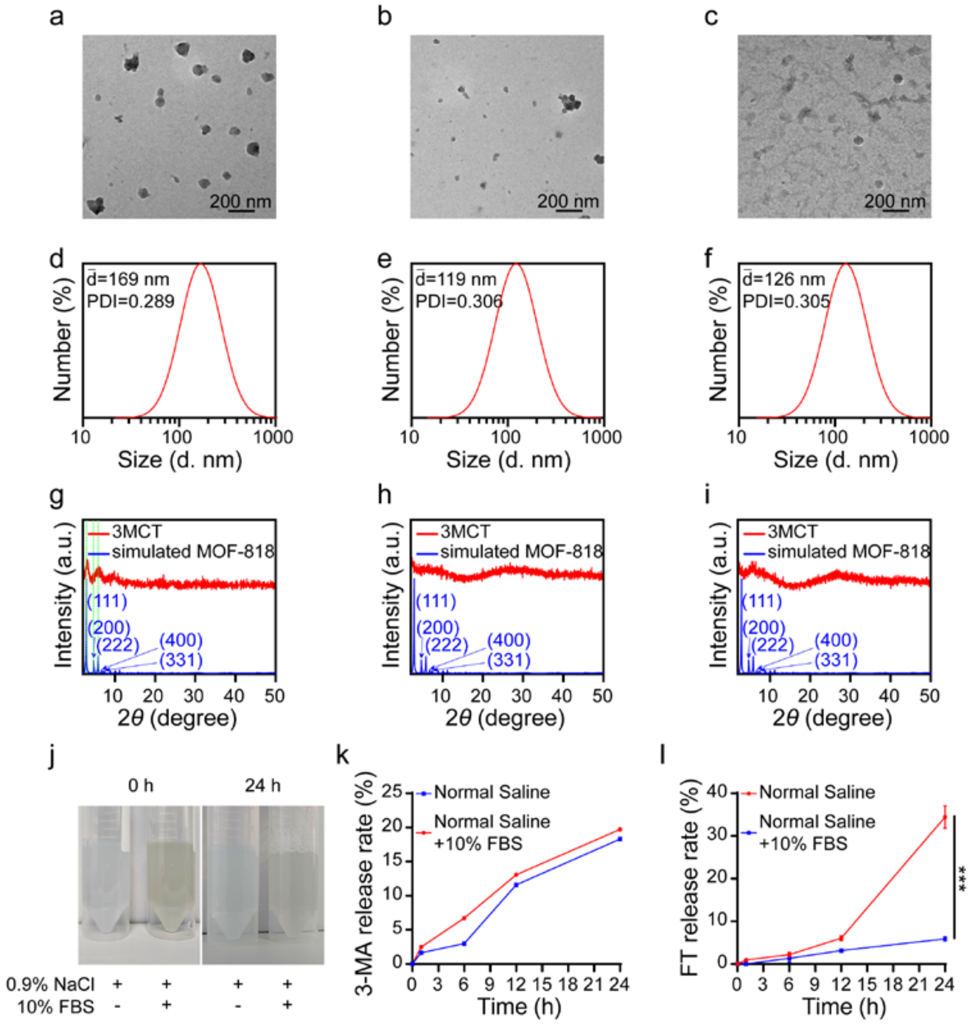

Figure S39. Stability assessment of 3MCT NPs. (a–c) TEM images: (a) as-prepared 3MCT NPs; (b) after incubation in normal saline at 37°C with 50 rpm shaking for 24 h; (c) after incubation in normal saline supplemented with 10% FBS under the same conditions. (d–f) Dynamic light scattering (DLS) profiles corresponding to the samples in (a–c). (g–i) X-ray diffraction (XRD) patterns of the same sample series. (j) Visual appearance of 3MCT NPs under the respective conditions. Drug release rates of 3-MA (k) and FT (l). FT, Fc-TRAIL. Data are given as mean ± SD (i: n = 3). ***$p < 0.001$.

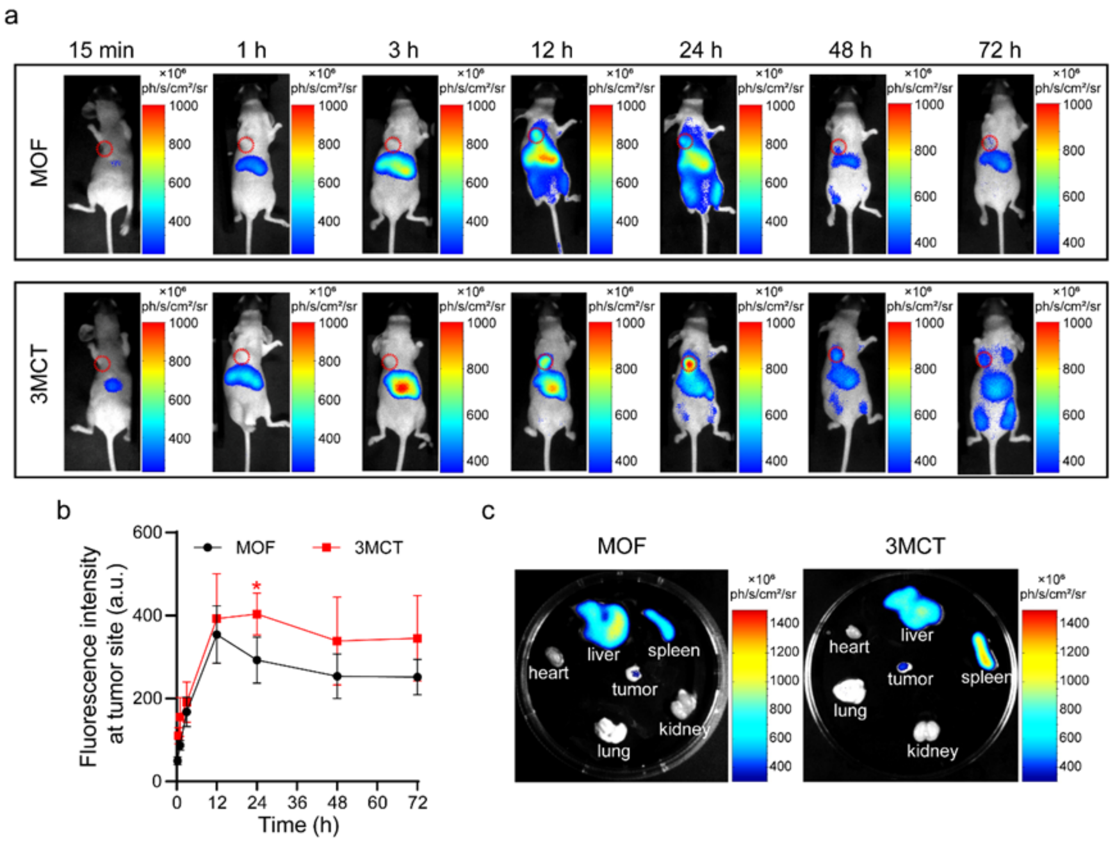

Figure S40. *In vivo* tumor targeting of 3MCT NPs. (a) The variations of fluorescence signals over time. The red dotted circle indicates the location of the tumor. (b) Statistical analysis of fluorescence intensity at tumor site over time. (c) Fluorescence signals of the *ex vivo* organs and tumors. Data are given as mean ± SD (n = 4).

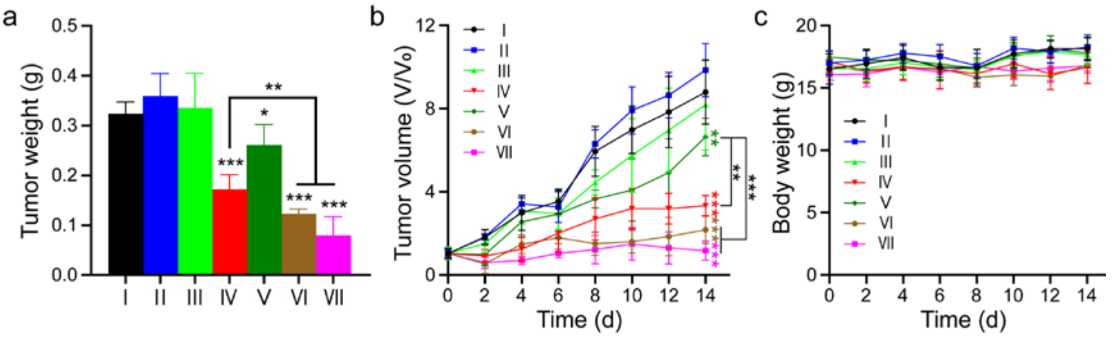

Figure S41. Tumor-targeting therapy of 3MCT NPs with APAP *in vivo*. (a) Mean weight of isolated tumors. Tumor growth curves (b) and body weight changes (c) from days 0 to 14. Groups I to VII are control, APAP, MOF-818, APAP+MOF-818, FT, APAP+MCT, and APAP+3MCT treatments, respectively. Data are given as mean ± SD (n = 5). *$p < 0.05$, **$p < 0.01$, ***$p < 0.001$.

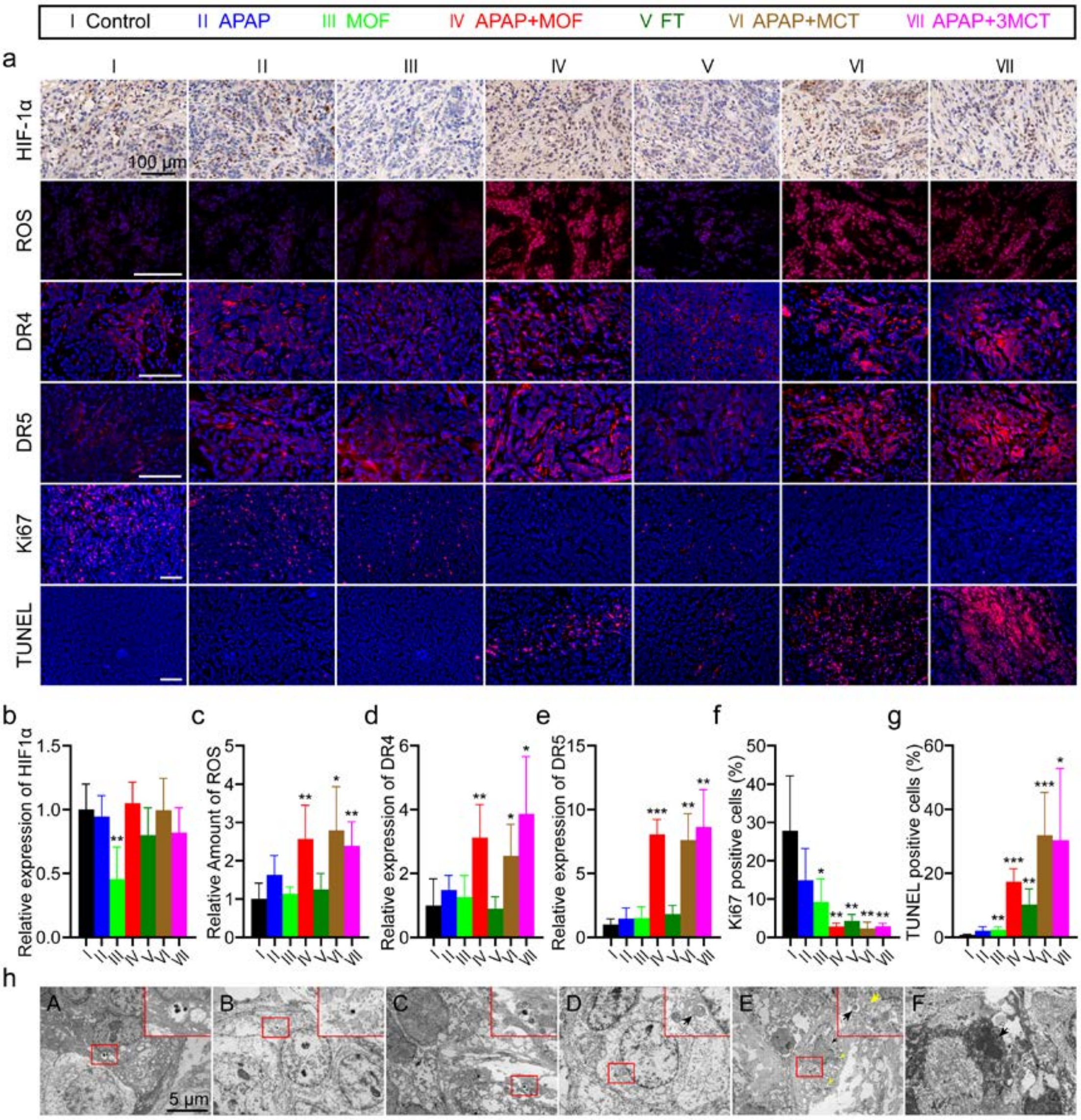

Figure S42. TME reshaping and anti-tumor activity induced by 3MCT and APAP *in vivo*. (a) The immunohistochemical staining of HIF-1α, fluorescent staining of ROS and TUNEL, and immunofluorescent staining of DR4, DR5 and Ki67 in the tumors of GFP-labeled HCC1806-bearing mice. The scale bar is 100 μm. Quantitative analyses of the signals changes of HIF-1α (b), ROS (c), DR4 (d), DR5 (e), Ki67 (f), and TUNEL (g). (h) TEM images of xenograft tumors. Endocytosis of MOF-818 NPs into tumor cells (A–C); the formation of phagophores (D, black arrows), autophagosomes (E, black arrows), and autolysosomes (E, yellow arrows); and apoptosis (F). The scale bar is 5 μm. Groups I to VII are control, APAP, MOF-818, APAP+MOF-818, FT, APAP+MCT, and APAP+3MCT treatments, respectively. Data are given as mean ± SD (n = 5). *$p < 0.05$, **$p < 0.01$, ***$p < 0.001$.

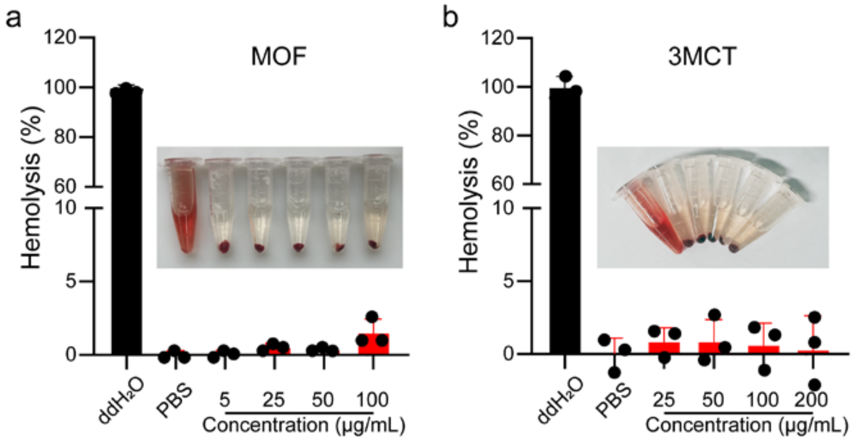

Figure S43. Hemolysis tests of MOF-818 (a) and 3MCT NPs (b). Data are given as mean ± SD (n = 3).

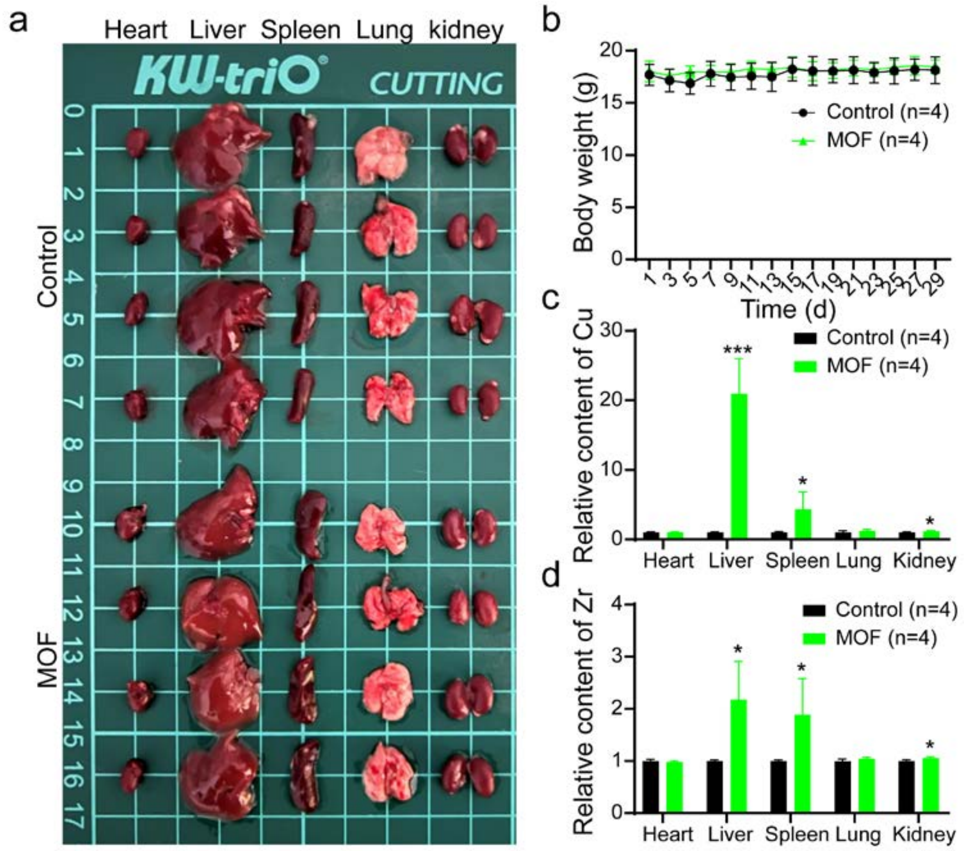

Figure S44. Biosafety and organ distribution assessments of MOF-818. BALB/c mice were subcutaneously injected once a day for 30 d. (a) Image of the *ex vivo* organs. (b) Body weight change from 0 d to 30 d. The contents of Cu (c) and Zr (d) in organs measured by inductively coupled plasma mass spectrometry (ICP-MS). Data are given as mean ± SD ($n = 4$). *$p < 0.05$, ***$p < 0.001$.

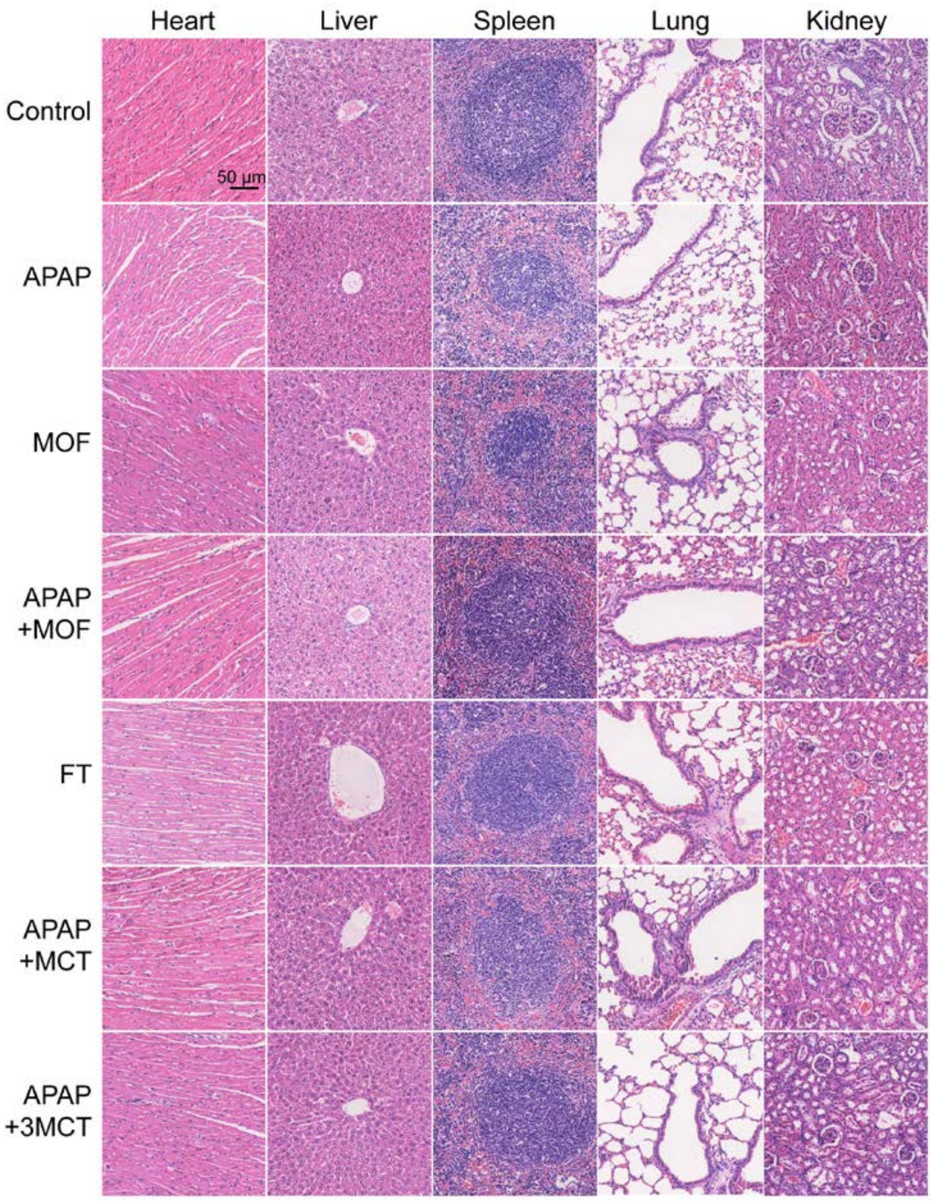

Figure S45. H&E staining images of the major organs from tumor-bearing mice with different treatments.

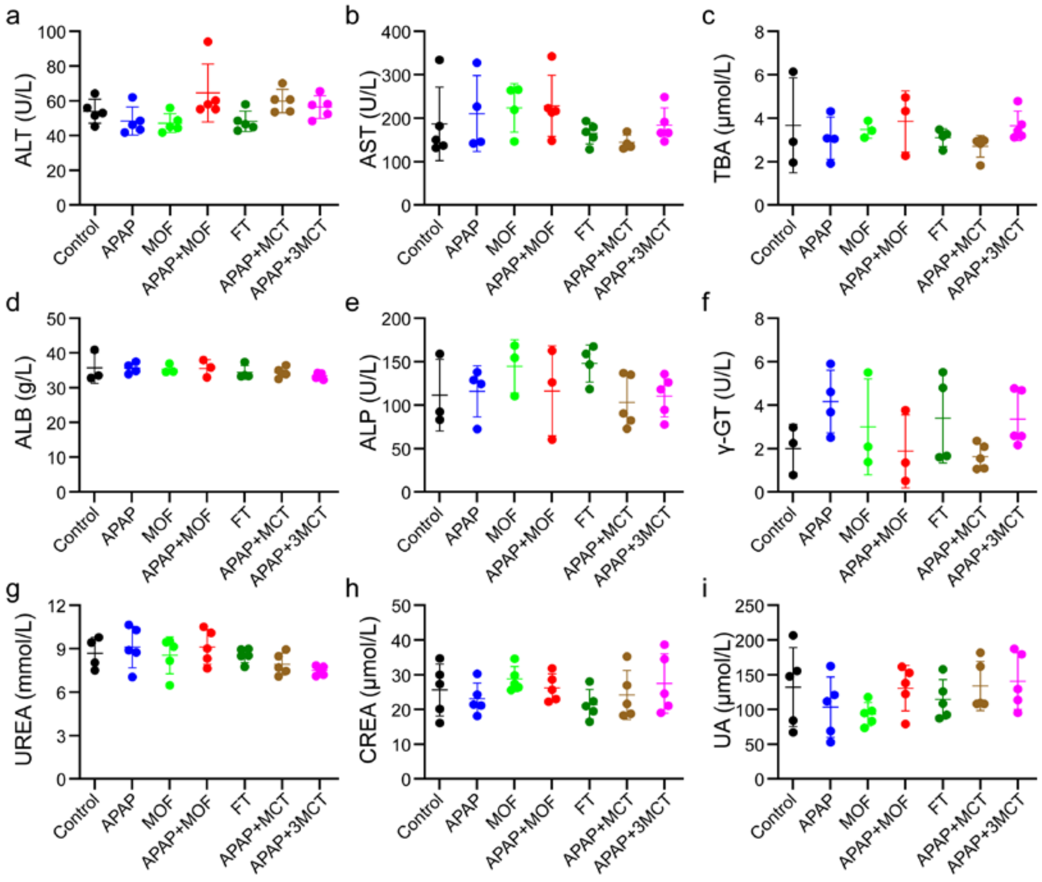

Figure S46. Determination of liver and kidney function indicators of GFP-labeled HCC1806-bearing mice under different treatments. The indicators included alanine transaminase (ALT, a), aspartate Aminotransferase (AST, b), total bile acid (TBA, c), albumin (ALB, d), alkaline phosphatase (ALP, e), γ-glutamyl transferase (γ-GT, f), Urea (UREA, g), creatinine (CREA, h), and uric acid (UA, i). Data are given as mean ± SD (n = 3~5).

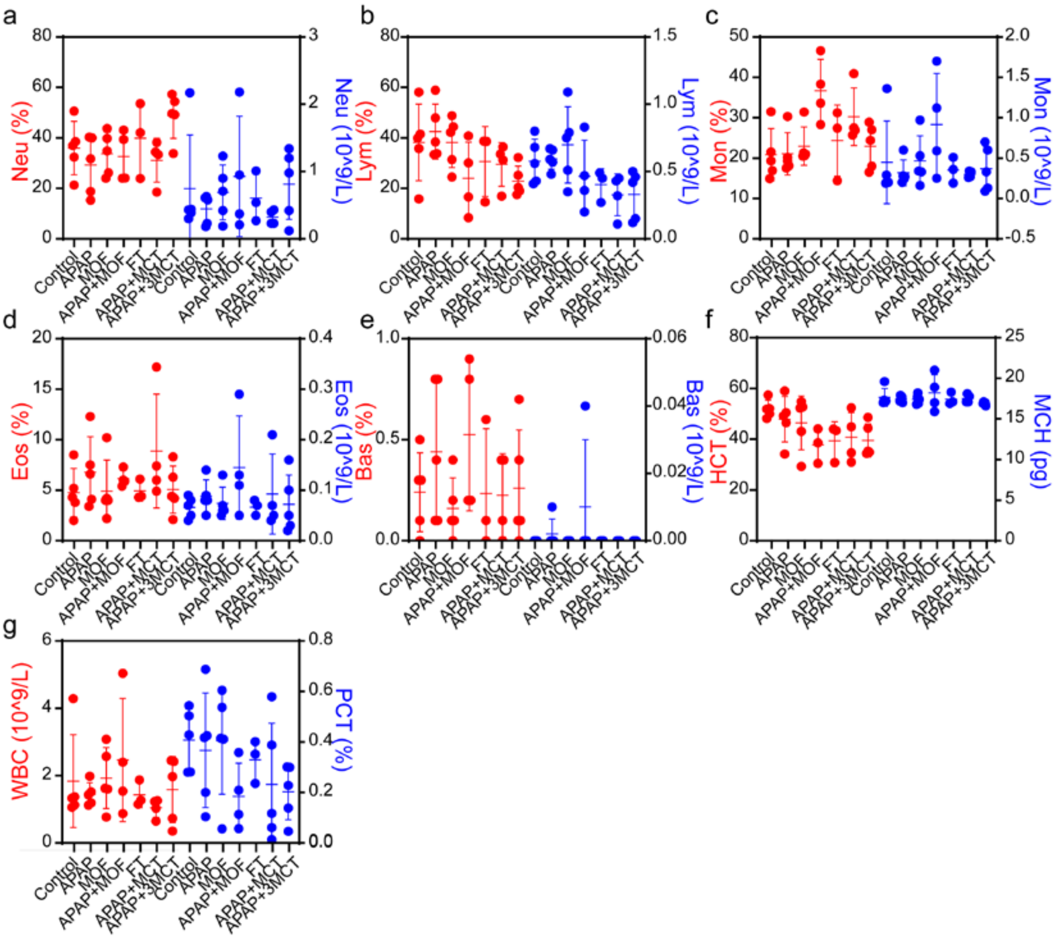

Figure S47. Blood routine examination of GFP-labeled HCC1806-bearing mice under different treatments. Data are given as mean ± SD (n = 3~5).

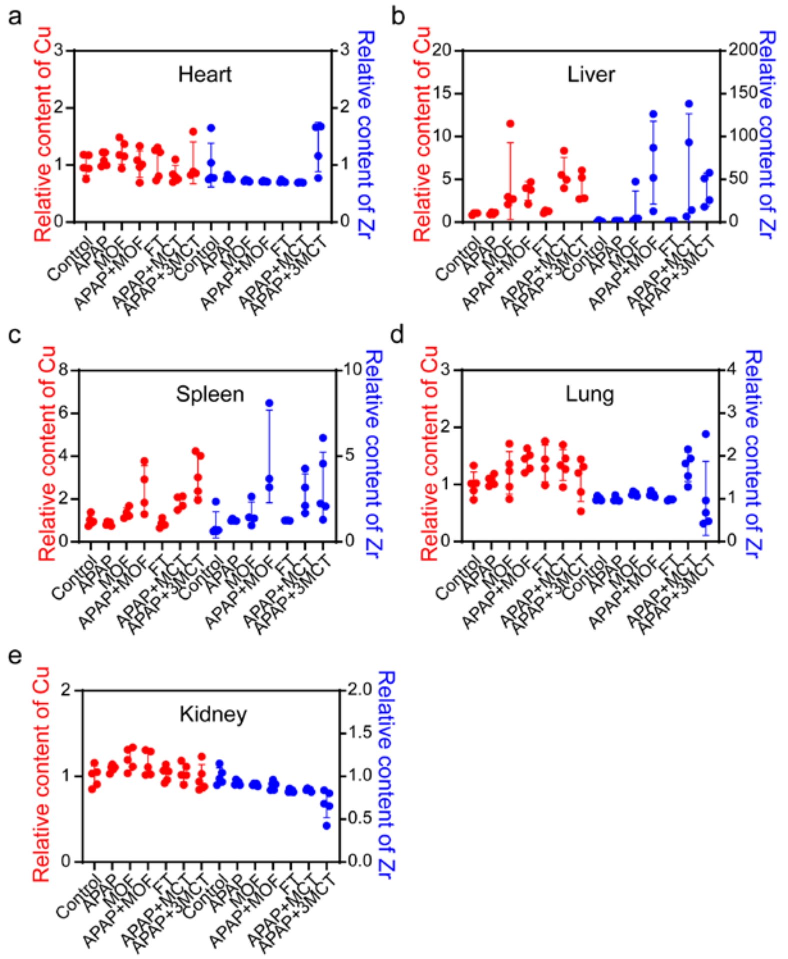

Figure S48. The contents of Cu and Zr in heart (a), liver (b), spleen (c), lung (d) and kidney (e) from GFP-labeled HCC1806-bearing mice under different treatments determined by ICP-MS. Data are given as mean ± SD (n = 3~5).

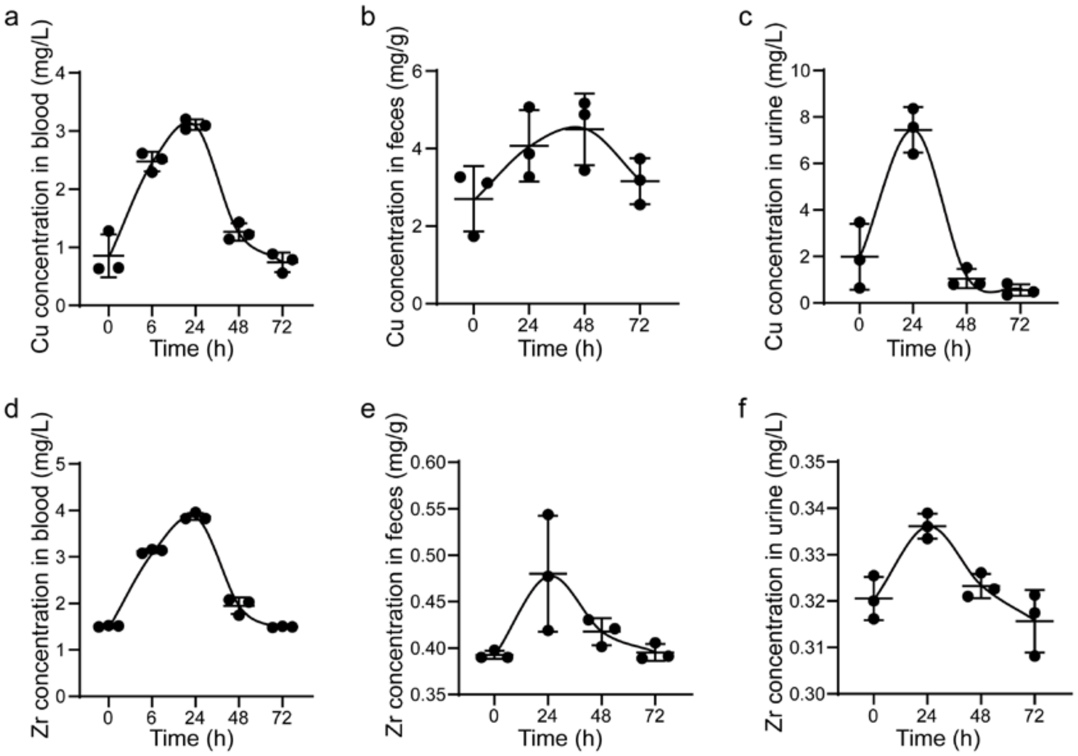

Figure S49. The contents of Cu and Zr determined by ICP-MS. The contents of Cu in blood (a), feces (b), and urine (c), and Zr in blood (d), feces (e), and urine (f) from BALB/c mice after subcutaneous injection of MOF-818. Data are given as mean ± SD (n = 3).

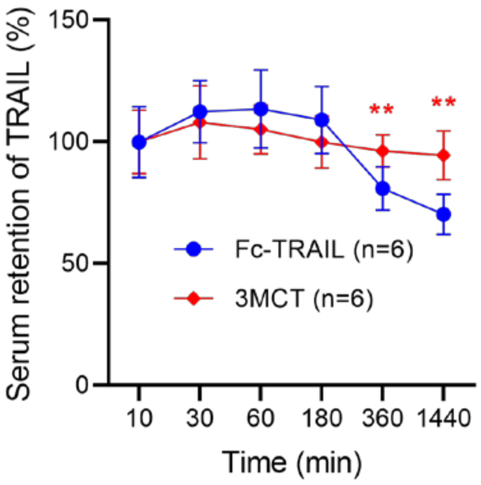

Figure S50. Changes in serum retention ratio of TRAIL following tail vein injection of Fc-TRAIL (FT) and 3MCT NPs to BALB/c mice. Data are given as mean ± SD (n = 6). **$p < 0.01$.

Supplemental references

[1] Liu Q, Song Y, Ma Y *et al*. Mesoporous cages in chemically robust MOFs created by a large number of vertices with reduced connectivity. *J Am Chem Soc* 2019; **141**: 488-96.

[2] Li M, Chen J, Wu W *et al*. Oxidase-like MOF-818 nanozyme with high specificity for catalysis of catechol oxidation. *J Am Chem Soc* 2020; **142**: 15569-74.

[3] Wu T, Huang S, Yang H *et al*. Bimetal biomimetic engineering utilizing metal–organic frameworks for superoxide dismutase mimic. *ACS Materials Lett* 2022; **4**: 751-7.

[4] Fan K, Xi J, Fan L *et al*. In vivo guiding nitrogen-doped carbon nanozyme for tumor catalytic therapy. *Nat Commun* 2018; **9**: 1440.

[5] Rahman I, Kode A, Biswas SK. Assay for quantitative determination of glutathione and glutathione disulfide levels using enzymatic recycling method. *Nat Protoc* 2006; **1**: 3159-65.

[6] Zhang LU, Wang Z, Zhang Y *et al*. Erythrocyte membrane cloaked metal–organic framework nanoparticle as biomimetic nanoreactor for starvation-activated colon cancer therapy. *ACS nano* 2018; **12**: 10201-11.

[7] Hansen TE and Johansen T. Following autophagy step by step. *BMC biol* 2011; **9**: 1-4.

[8] Kim D, Paggi JM, Park C *et al*. Graph-based genome alignment and genotyping with HISAT2 and HISAT-genotype. *Nat biotechnol* 2019; **37**: 907-15.

[9] Liao Y, Smyth GK, Shi W. featureCounts: an efficient general purpose program for assigning sequence reads to genomic features. *Bioinformatics* 2014; **30**: 923-30.

[10] Love MI, Huber W, Anders S. Moderated estimation of fold change and dispersion for RNA-seq data with DESeq2. *Genome biol* 2014; **15**: 1-21.

[11] Subramanian A, Tamayo P, Mootha VK *et al*. Gene set enrichment analysis: a knowledge-based approach for interpreting genome-wide expression profiles. *P*

Natl Acad Sci USA 2005; **102**: 15545-50.

[12] Yu K, Li M, Chai H *et al*. MOF-818 nanozyme-based colorimetric and electrochemical dual-mode smartphone sensing platform for in situ detection of H_2O_2 and H_2S released from living cells. *Chem Eng J* 2023; **451**: 138321.

[13] Duan W, Qiu Z, Cao S *et al*. Pd–Fe3O4 Janus nanozyme with rational design for ultrasensitive colorimetric detection of biothiols. *Biosens Bioelectron* 2022; **196**: 113724.

[14] Ge C, Wu R, Chong Y *et al*. Synthesis of Pt hollow nanodendrites with enhanced peroxidase‐like activity against bacterial infections: implication for wound healing. *Adv Funct Mater* 2018; **28**: 1801484.

[15] Xu W, Jiao L, Yan H *et al*. Glucose oxidase-integrated metal–organic framework hybrids as biomimetic cascade nanozymes for ultrasensitive glucose biosensing. *ACS Appl Mater Interfaces* 2019; **11**: 22096-101.

[16] Gao L, Zhuang J, Nie L *et al*. Intrinsic peroxidase-like activity of ferromagnetic nanoparticles. *Nat Nanotechnol* 2007; **2**: 577-83.
