# Supplementary figures and images for: A multienzyme-mimicking nanoplatform induces disulfidptosis/cuproptosis/apoptosis for tumor therapy
Source: Natl Sci Rev. 2026 May 27;13(12):nwag316. doi: 10.1093/nsr/nwag316 (PMC13317449; doi:10.1093/nsr/nwag316)

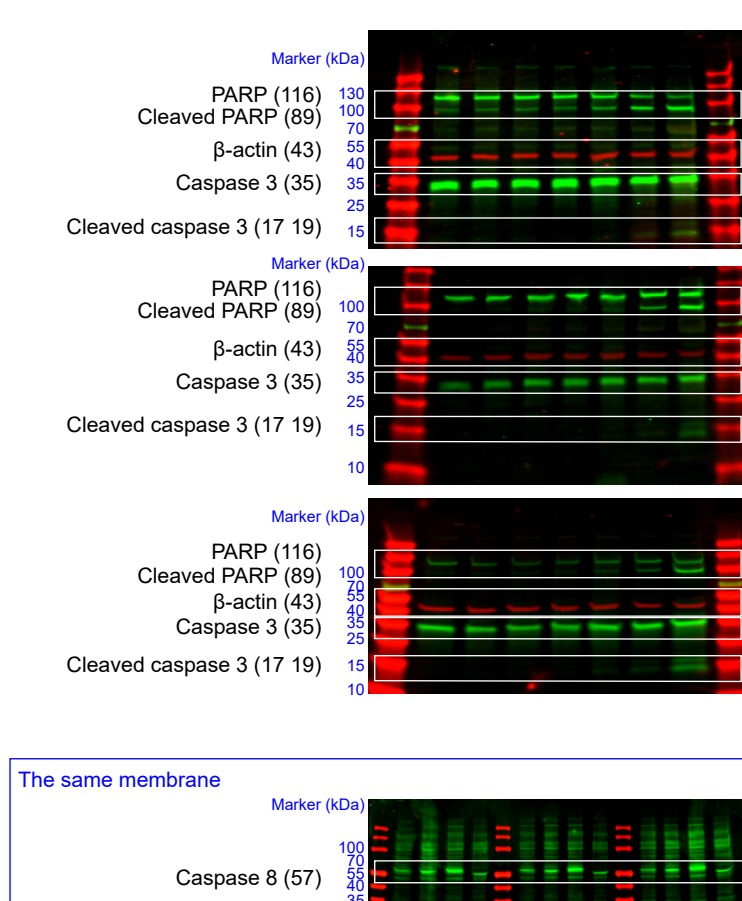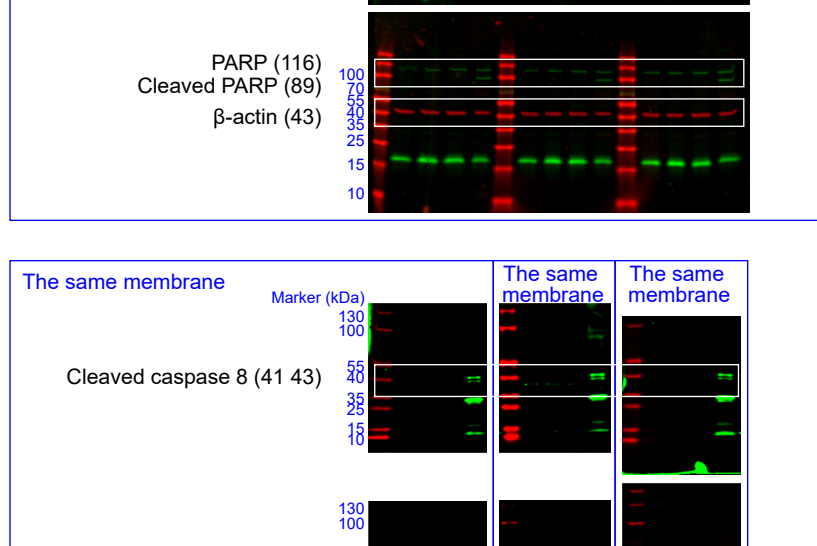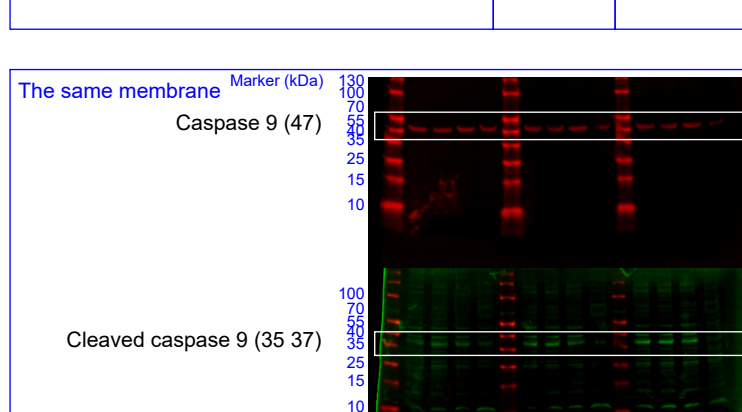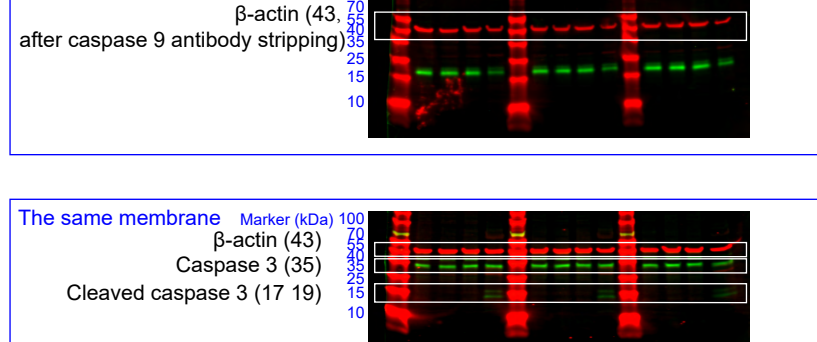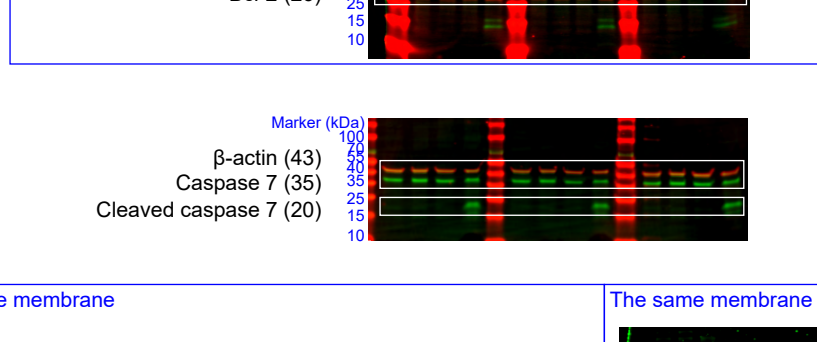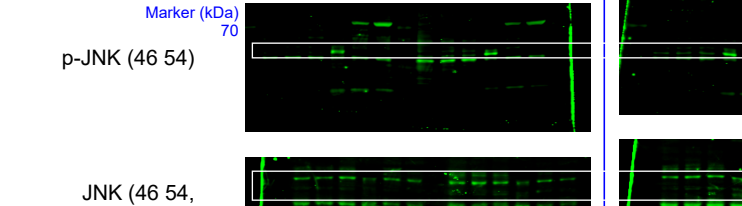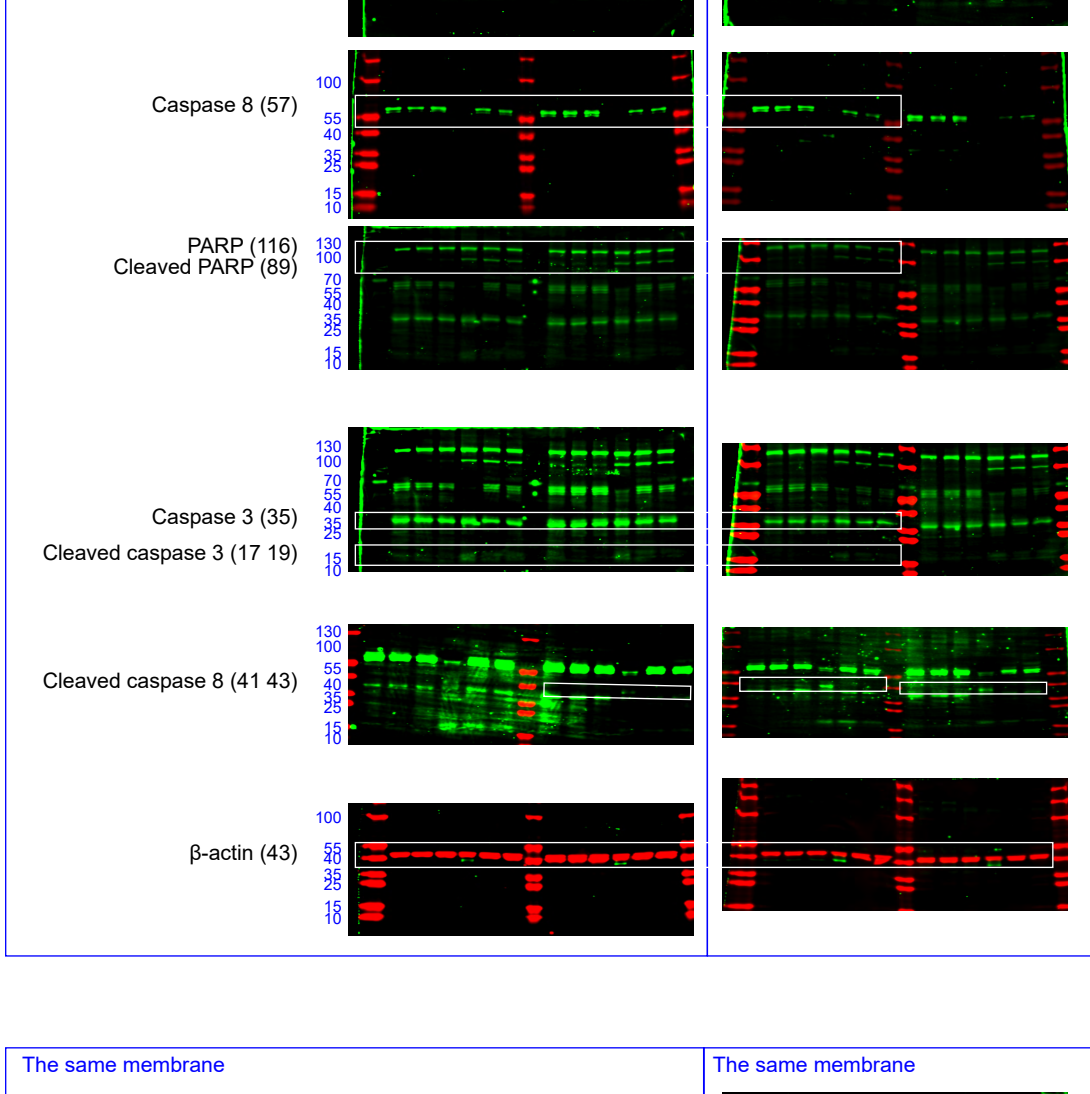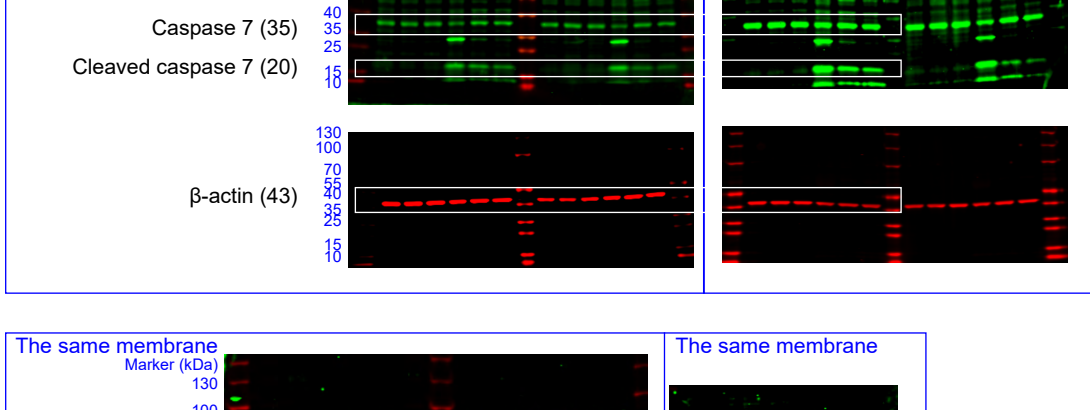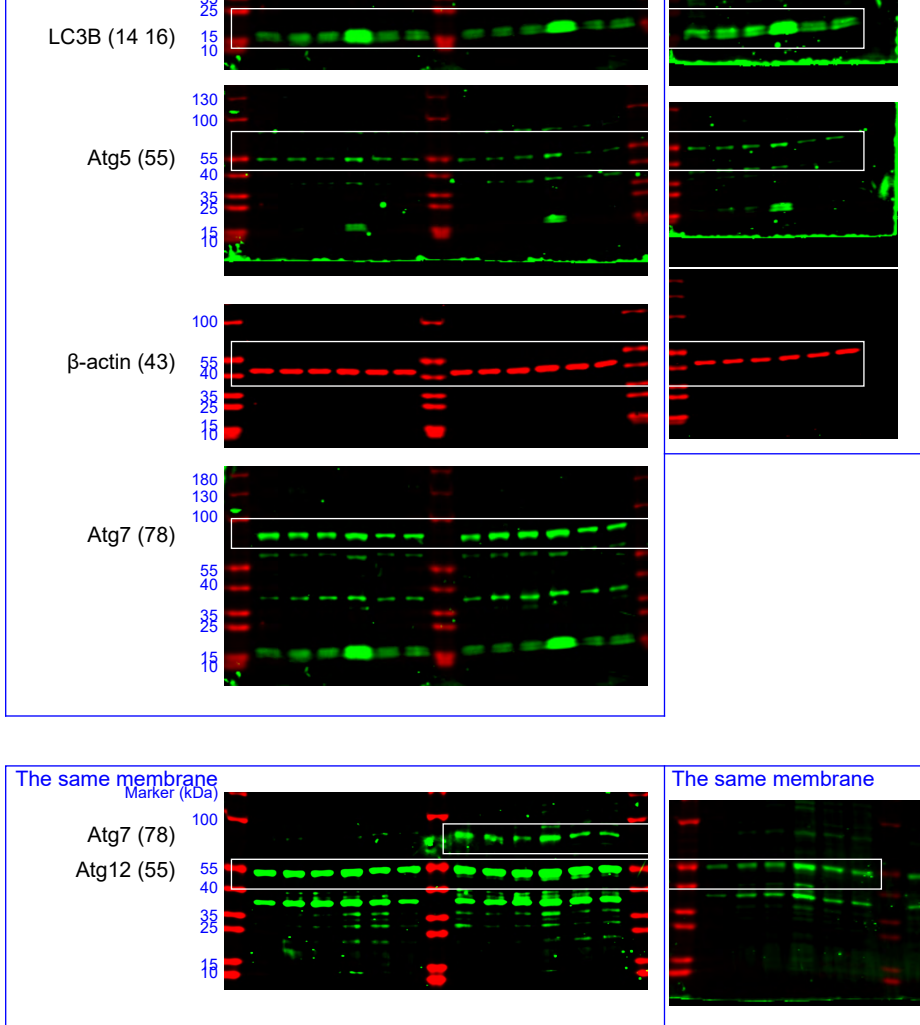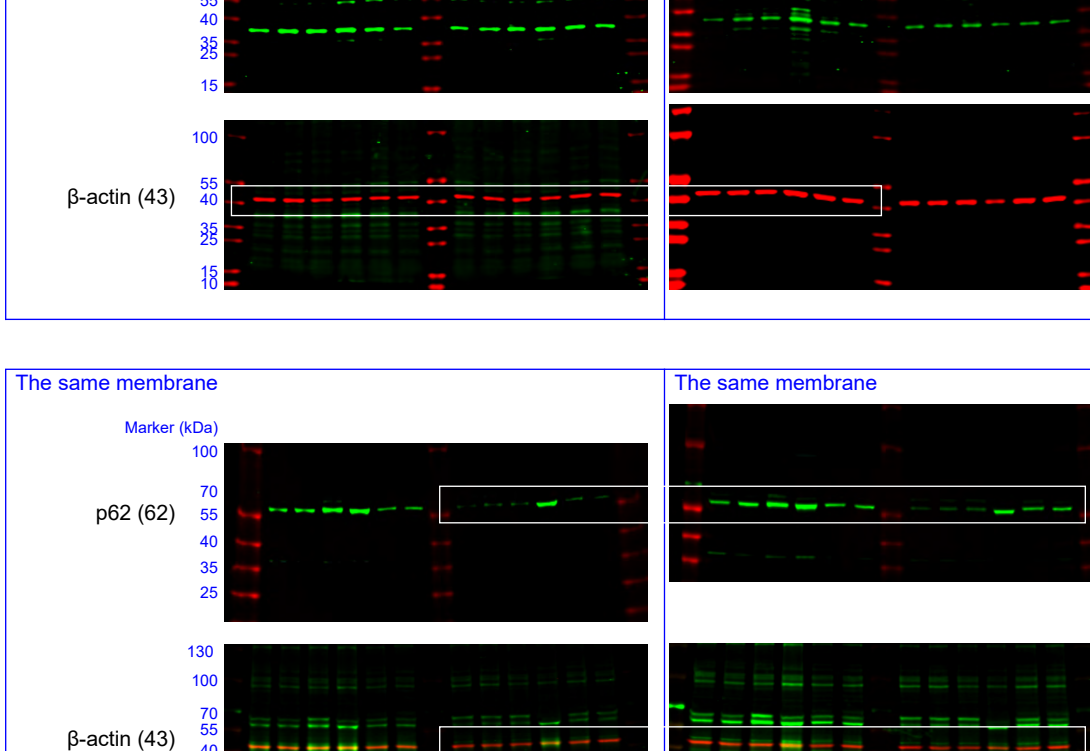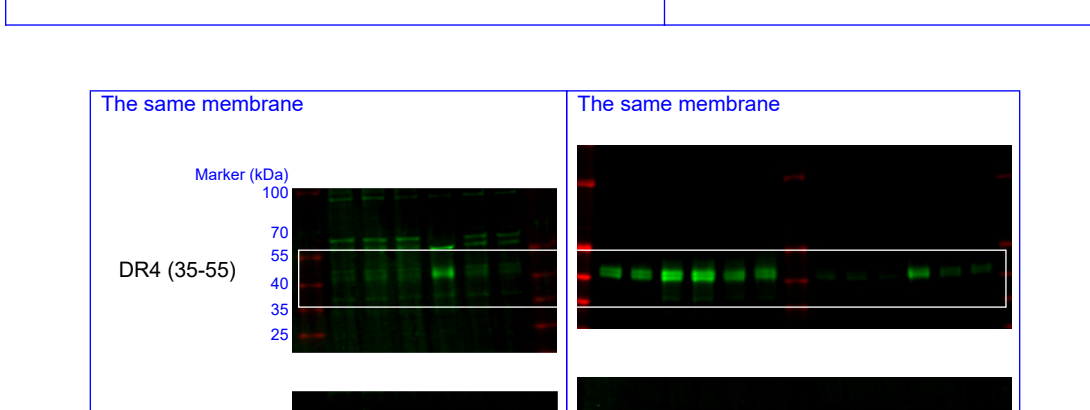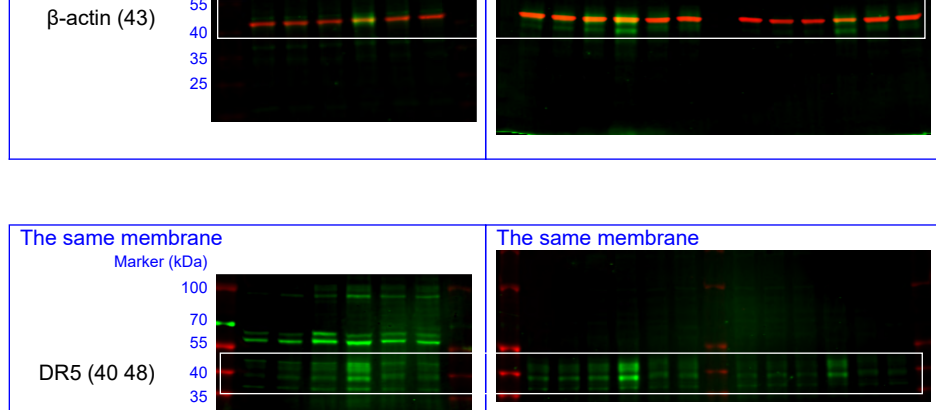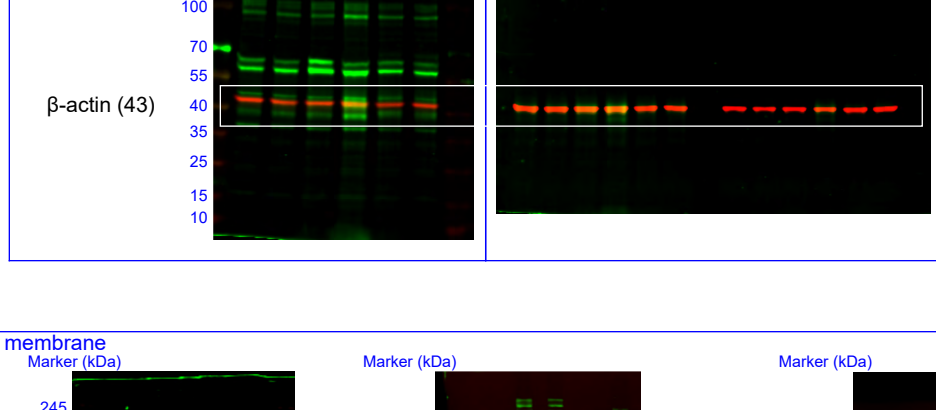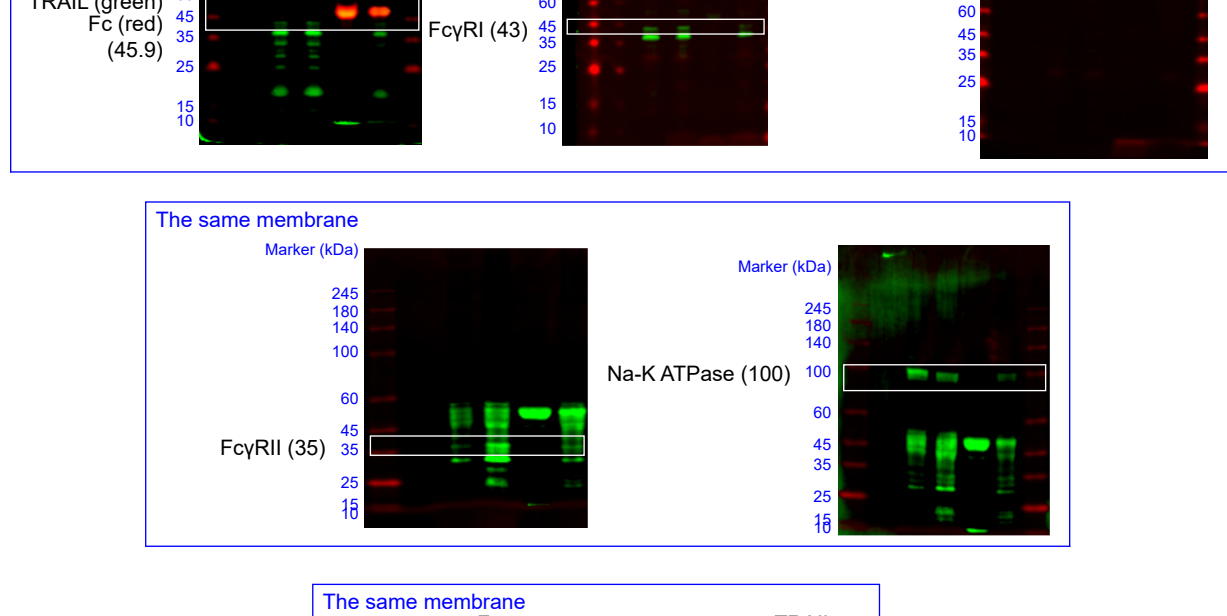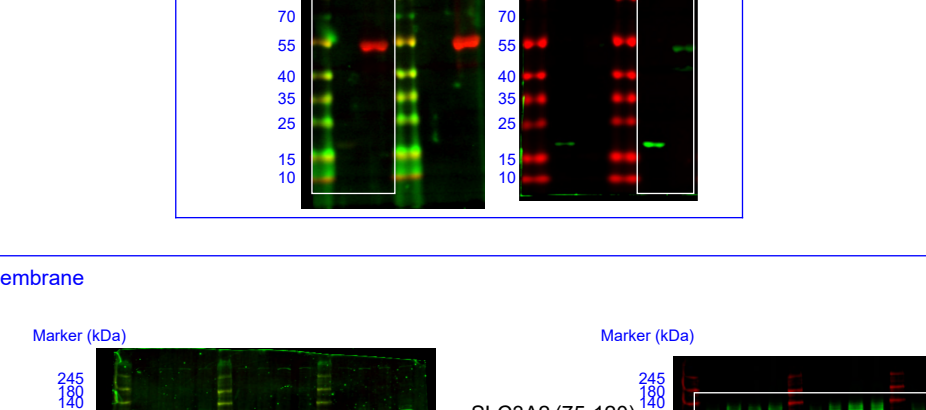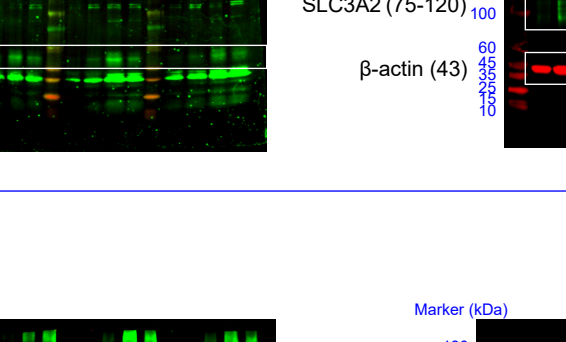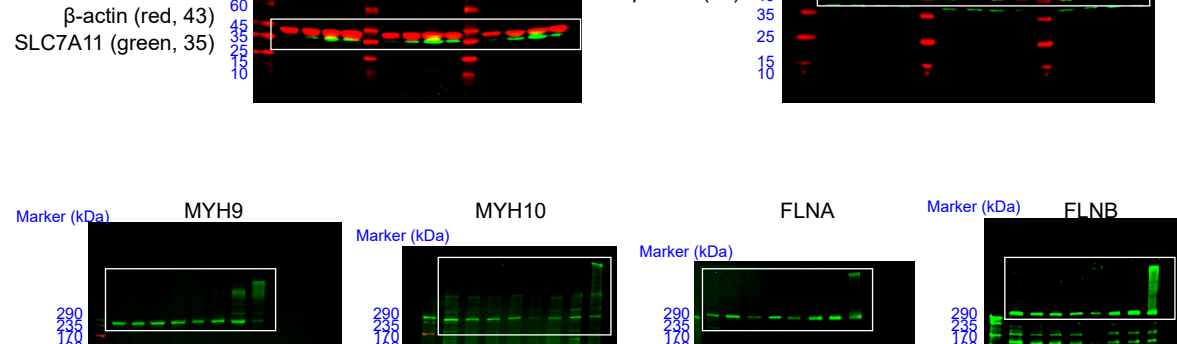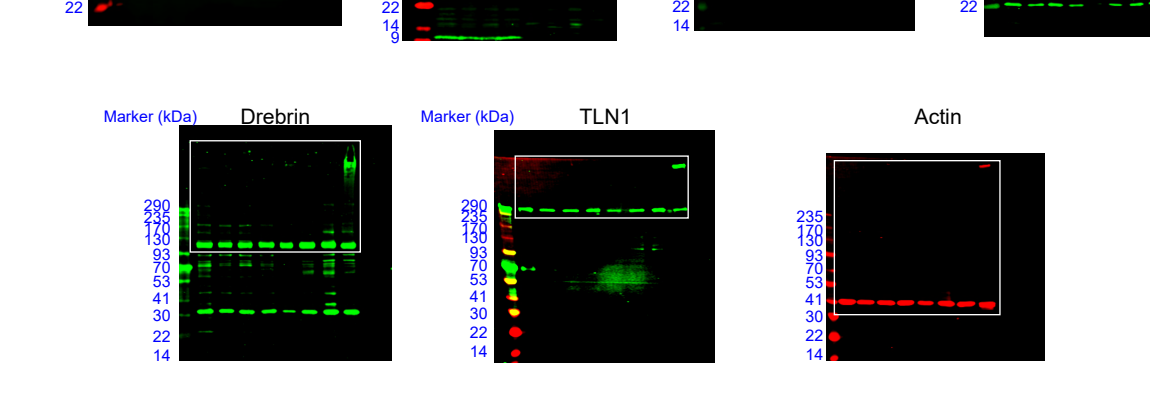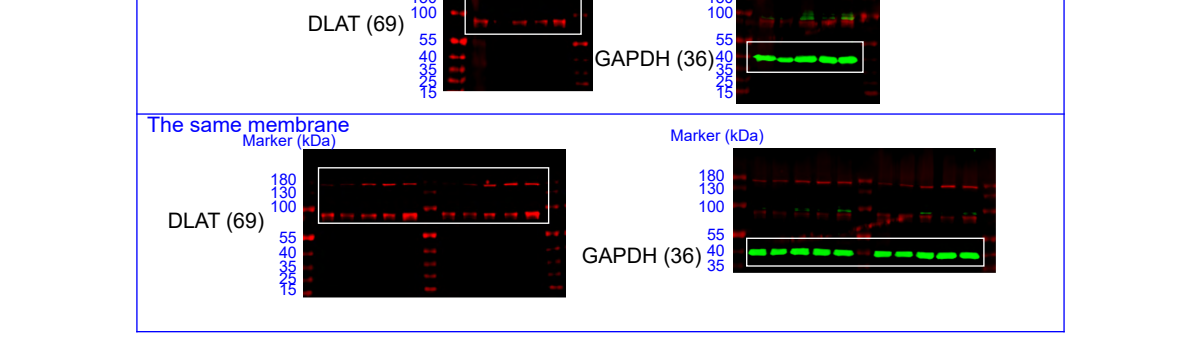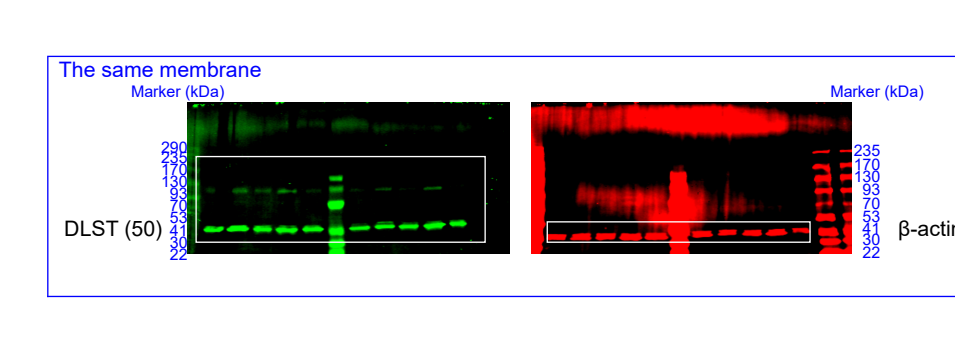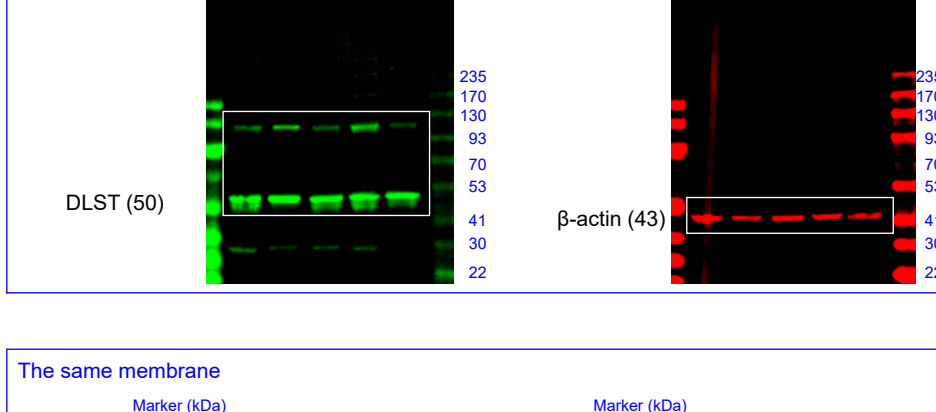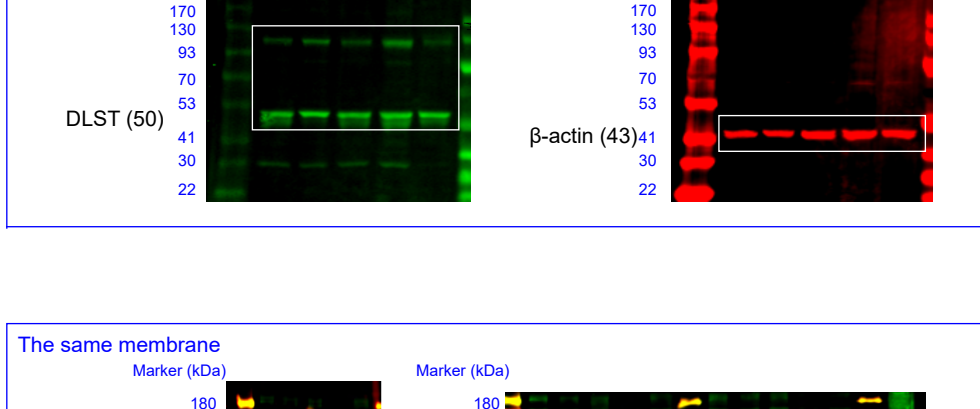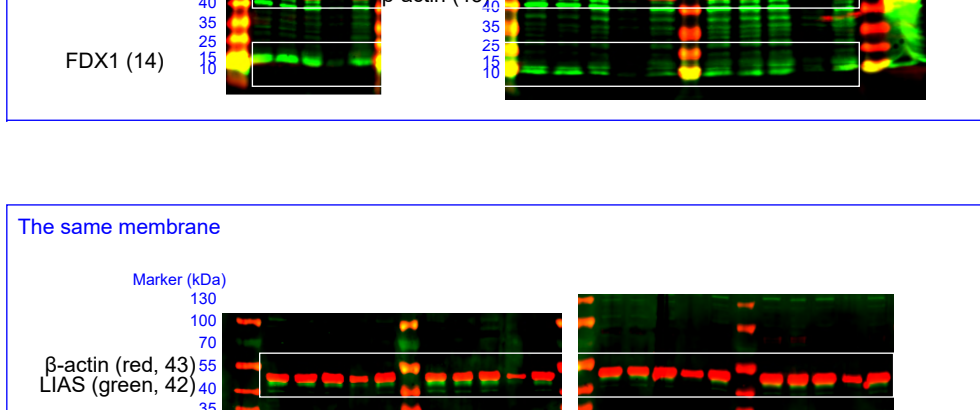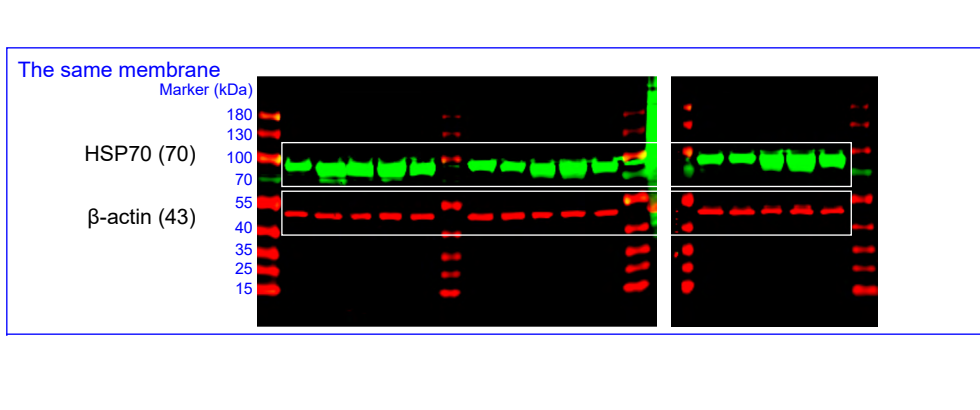

Supplement: nwag316_Supplemental_Files [file nwag316_supplemental_files.zip › nwag316-Supplementary_Material_2.pdf]
